# KinasePhos 3.0: Redesign and Expansion of the Prediction on Kinase-specific Phosphorylation Sites

**DOI:** 10.1016/j.gpb.2022.06.004

**Published:** 2022-07-01

**Authors:** Renfei Ma, Shangfu Li, Wenshuo Li, Lantian Yao, Hsien-Da Huang, Tzong-Yi Lee

**Affiliations:** 1 Warshel Institute for Computational Biology, School of Medicine, The Chinese University of Hong Kong, Shenzhen 518172, China; 2 School of Life Sciences, University of Science and Technology of China, Hefei 230027, China; 3 School of Science and Engineering, The Chinese University of Hong Kong, Shenzhen 518172, China; 4 School of Life and Health Sciences, School of Medicine, The Chinese University of Hong Kong, Shenzhen 518172, China

**Keywords:** Kinase-specific phosphorylation, Phosphorylation site prediction, Phosphorylation, SHAP feature importance, Kinase

## Abstract

The purpose of this work is to enhance KinasePhos, a machine learning-based **kinase-specific phosphorylation site prediction** tool. Experimentally verified kinase-specific phosphorylation data were collected from PhosphoSitePlus, UniProtKB, the GPS 5.0, and Phospho.ELM. In total, 41,421 experimentally verified kinase-specific phosphorylation sites were identified. A total of 1380 unique kinases were identified, including 753 with existing classification information from KinBase and the remaining 627 annotated by building a phylogenetic tree. Based on this kinase classification, a total of 771 predictive models were built at the individual, family, and group levels, using at least 15 experimentally verified substrate sites in positive training datasets. The improved models demonstrated their effectiveness compared with other prediction tools. For example, the prediction of sites phosphorylated by the protein kinase B, casein kinase 2, and protein kinase A families had accuracies of 94.5%, 92.5%, and 90.0%, respectively. The average prediction accuracy for all 771 models was 87.2%. For enhancing interpretability, the SHapley Additive exPlanations (SHAP) method was employed to assess feature importance. The web interface of KinasePhos 3.0 has been redesigned to provide comprehensive annotations of kinase-specific phosphorylation sites on multiple proteins. Additionally, considering the large scale of phosphoproteomic data, a downloadable prediction tool is available at https://awi.cuhk.edu.cn/KinasePhos/download.html or https://github.com/tom-209/KinasePhos-3.0-executable-file.

## Introduction

Protein phosphorylation is an important eukaryotic post-translational modification [1]. It involves the transfer of a phosphate group from adenosine triphosphate (ATP) to specific amino-acid residues in the substrate. Phosphorylation is catalyzed by a number of protein kinases, which regulate a variety of signaling pathways and biological functions important in deoxyribonucleic acid (DNA) repair, transcriptional regulation, apoptosis, immune response, signaling, metabolism, proliferation, and differentiation [2], [3], [4], [5], [6], [7]. Dysregulation of intracellular phosphorylation networks contributes to the occurrence and development of multiple multifactorial diseases, including cancer, cardiovascular disease, obesity, and others [8], [9], [10]. Therefore, regulating phosphorylation networks by mediating kinase activity has become an attractive therapeutic strategy [11] with kinases being one of the most important drug targets [12], [13]. Thus, linking dysregulated phosphorylation sites to candidate kinase targets is critical, both for the study of disease mechanisms and the development of therapeutic kinase inhibitors [14], [15].

Due to the development of mass spectrometry and new enrichment methods, more and more experimentally detected post-translation modifications were confirmed and many related databases emerged [16], [17], [18]. The number of experimentally detected phosphorylated sites has increased dramatically in recent years [19]. For example, deep phosphoproteome analysis of *Schistosoma mansoni* detected 15,844 unique phosphopeptides mapping to 3176 proteins [20]. Phosphoproteomics can provide important information about protein phosphorylation sites, but the responsible kinases cannot be directly derived from such data. In fact, the kinases for a vast majority of phosphorylation sites are still unknown due to a lack of adequate evidence [21]. To address this problem, many tools have been developed to predict kinase-specific phosphorylation sites in proteins, as computational prediction tools developed for other types of post-translation modifications [22], [23], [24]. For example, PhosphoPredict was developed to predict kinase-specific substrates and their associated phosphorylation sites for 12 human kinases and their families by combining protein sequences and functional features [25]. Neural networks were applied by NetPhos 3.1 to predict phosphorylation sites in eukaryotic proteins for 17 kinases [26]. Quokka was introduced to predict kinase family-specific phosphorylation sites at the proteomic scale in a high-throughput and cost-effective manner [27]. Musite provided a unique method that trained models with a bootstrap aggregating procedure, as well as integrated sequence cluster information around phosphorylation sites, protein disorder scores, and amino acid frequencies to predict general and kinase-specific phosphorylation sites [28]. The Group-based Prediction System (GPS) 5.0 tool employed two novel methods, position weight determination (PWD) and scoring matrix optimization (SMO), to replace the motif length selection (MLS) method for refining the prediction of kinase-specific phosphorylation sites [29]. In addition, the conditional random field (CRF) model (CRPhos) [30] and support vector machines (SVM) model (PredPhospho) have been employed to predict the phosphorylation sites [31]. These tools have made outstanding progress in protein phosphorylation studies.

In 2005, our group developed KinasePhos 1.0 to identify protein kinase-specific phosphorylation sites [32]. This tool constructed models from kinase-specific groups of phosphorylation sites based on the profile hidden Markov model (HMM). Subsequently, SVM with the protein sequence profiles and protein coupling patterns was applied to update the tool to version 2.0 [33]. The datasets available for training are constantly expanding owing to the rapid development of phosphorylation-related research. Therefore, in this study, we introduced KinasePhos 3.0, with improved kinase-specific phosphorylation site prediction. We collected experimental identifications of kinase-specific phosphorylation sites from the PhosphoSitePlus [34], UniProtKB [35], GPS 5.0 [29], and Phospho.ELM [36] databases. Redundant data were removed after translating the kinase and substrate names into unique UniProtKB identifiers (IDs). Finally, 41,421 empirically determined, kinase-specific phosphorylation sites were obtained for use as the training data set, which was a great improvement over the training of version 2.0, which involved 16,543 kinase-specific phosphorylation sites. We also assigned kinases to groups, families, or subfamilies according to sequence similarity and the classification method of KinBase [37]. Then, according to these classifications, we used both SVM and eXtreme Gradient Boosting (XGBoost) algorithms to construct 771 prediction models at the kinase group, kinase family, and individual kinase levels, in contrast to 60 predictive models at the individual kinase level in version 2.0. Using these models, specific phosphorylation sites for ten groups, 81 families, and 302 kinases were identified. We also plotted the SHapley Additive exPlanations (SHAP) values of feature groups for each prediction result, which makes the tool more interpretable than version 2.0, as well as other tools in this field. Using SHAP values, users can subdivide the prediction to show the impact of each feature group (*i.e.*, features related to specific residues in this study) on the results. Additionally, a standalone version of KinasePhos 3.0 was developed, making it more convenient for users with larger phosphoproteomic datasets than KinasePhos 2.0.

## Method

### Schematic of the proposed KinasePhos 3.0

**Figure 1:**
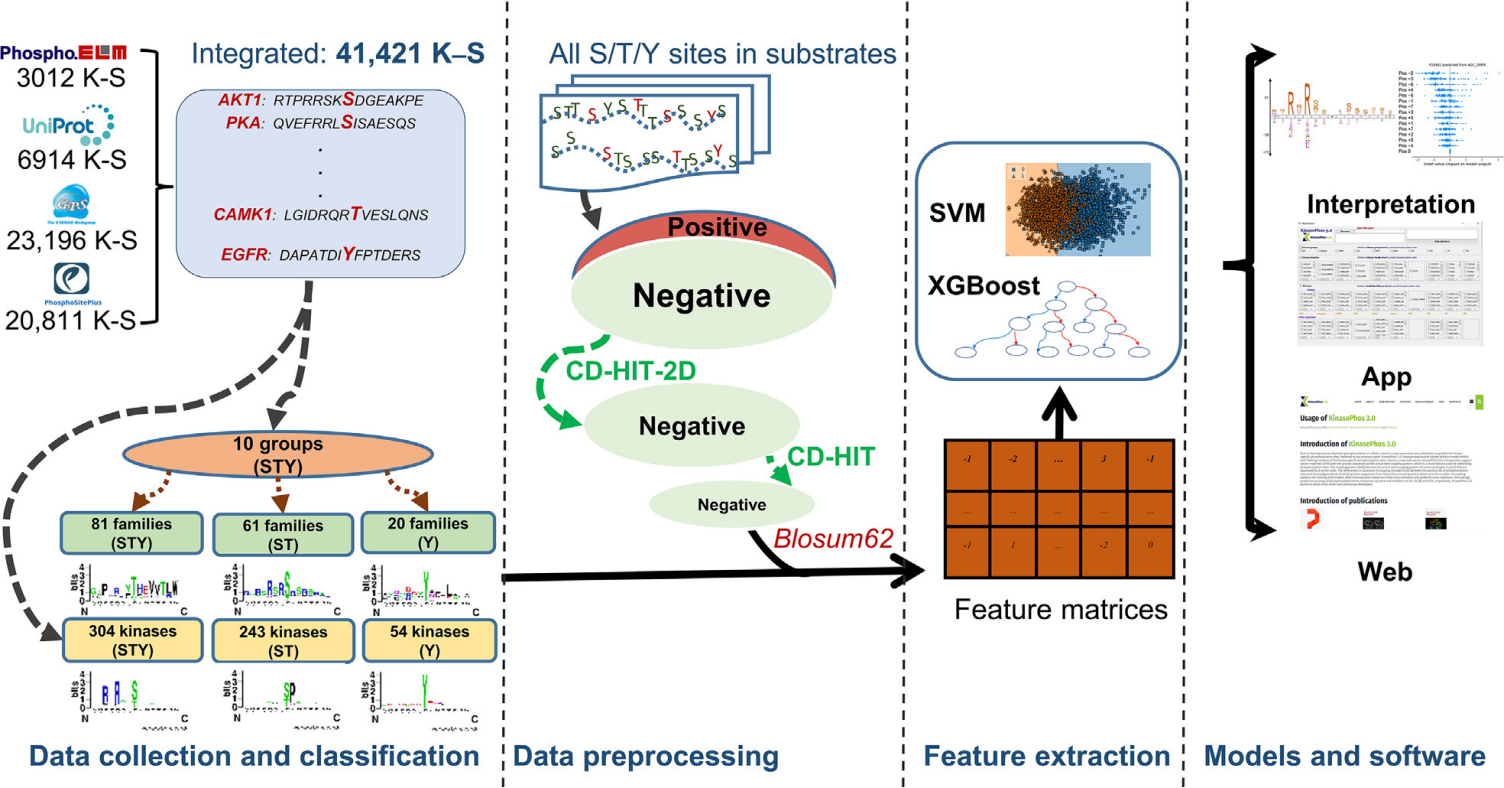
**Schematic of KinasePhos 3.0 development** The procedures include data collection, processing, modeling, and website function development. K–S, kinase-substrate; SVM, support vector machine; XGBoost, eXtreme Gradient Boosting.

Figure 1 depicts a schematic of this study that includes kinase-specific phosphorylation site data collection, kinase group and family classifications, feature extractions, machine learning-based kinase-specific phosphorylation site prediction model development, and presentation of results. The novelties of this study are reflected in three perspectives. First, to our knowledge, the experimentally verified kinase-specific phosphorylation-site data used in this study are, to date, the most comprehensive compared to all existing kinase-specific phosphorylation site prediction tools, such as GPS 5.0 and KinasePhos 2.0. Second, we obtained 771 prediction models, with at least 15 kinase-specific phosphorylation sites considered in each. Thus, the minimum number of positive sites for a single model was greater than that of some other tools. For example, GPS 5.0 includes prediction models for clusters with no less than three positive sites. Third, to increase the feature interpretability of these prediction models, SHAP was integrated into KinasePhos 3.0.

### Kinase–substrate data collection

The experimentally verified kinase-specific phosphorylation sites used in this study were collected from four phosphorylation-associated resources: GPS 5.0 [29], Phospho.ELM [36], PhosphoSitePlus [34], and UniProtKB [35]. Although GPS 5.0, Phospho.ELM, and PhosphoSitePlus provided downloadable, experimentally verified, and kinase-specific phosphorylation sites, their data is not frequently updated to reflect the increase in experimentally verified phosphorylated sites. In contrast, UniProtKB has a standard 8-week release cycle [35]. Therefore, we additionally curated experimentally verified, kinase-specific phosphorylation sites from UniProtKB to assemble the most comprehensive database. As depicted in Figure 1, 23,196, 3012, 20,811, and 6914 experimentally verified kinase-specific phosphorylation sites were retrieved from GPS 5.0, Phospho.ELM, PhosphoSitePlus, and UniProtKB, respectively. After eliminating redundancies, 41,421 sites remained, of which, the kinases for 28,369 had UniProtKB IDs. In contrast, the kinases for the remaining 13,052 sites lack UniProtKB IDs, primarily because only their kinase family types, instead of kinase names, are provided.

We converted all the kinase names in our kinase–substrate dataset into UniProtKB entry names. Then, we used the classification annotations of kinomes and their sequence information from KinBase as the annotated dataset [37]. By searching the UniProtKB database, gene names were converted to UniProtKB IDs. The collected and annotated human kinome datasets were merged and converted to FASTA format. Multiple sequence alignments were performed using the Multiple Alignment using Fast Fourier Transform (MAFFT) program [38]. FastTree was then employed to infer kinetic maximum likelihood phylogenetic trees from the kinase sequence alignments [39]. We assumed that homologous proteins have consistent domains represented by closer distances in the phylogenetic tree. Therefore, based on the classification data from KinBase and the generated tree, kinases could be annotated to different clusters at the group, family, and subfamily levels [40]. In addition, we obtained kinase domain data from protein families (PFAM) and simple modular architecture research tool (SMART) databases to confirm the results of our classification annotation [41], [42]. TreeGraph 2 and the Interactive Tree Of Life (iTOL) were used to visualize the annotations [43], [40].

### Model development

The classical BLOSUM62 substitute matrix has been widely employed to encode sequence data [29], [33], [44], [45] and was used in this study. For GPS 5.0, the SVM showed higher performance in kinase-specific phosphorylation site predictive models compared to the random forest (RF) and k-nearest neighbor (KNN) [29] methods. Additionally, XGBoost [46], an efficient implementation of gradient boosted decision trees, is suitable for web server applications for a faster response owing to its model performance and execution speed. Therefore, SVM and XGBoost were used to train the prediction models. The development, testing, and validation of these algorithms were implemented using Python 3.8.

The performance of the kinase-specific phosphorylation site prediction models was assessed via classification accuracy and two other metrics, precision and recall, as indicators of reliability. The F1_score, a more comprehensive quantifier of model reliability, and the area under the receiver operating characteristic (ROC) curve (AUC) were also computed. These performance measures are defined as:

(1) $$\mathrm{accuracy}=\frac{\mathrm{TP}+TN}{\mathrm{TP}+FP+FN+TN}$$

(2) $$\mathrm{recall}=\frac{\mathrm{TP}}{\mathrm{TP}+FN}$$

(3) $$\mathrm{precision}=\frac{\mathrm{TP}}{\mathrm{TP}+FP}$$

(4) $$F1\_score=\frac{2\times precision\times recall}{\mathrm{precision}+recall}$$

where TP, TN, FP, and FN represent true positives, true negatives, false positives, and false negatives, respectively. Weighted accuracy, weighted recall, weighted precision, and weighted F1_score are the weighted mean of accuracy, recall, precision, and F1_score with weights equal to the class probability, respectively.

### Feature interpretation with SHAP

Because explainable machine learning offers the potential to provide more insights into model behavior, the interpretability of machine learning models has received significant attention, along with the popularity of machine learning algorithms. Several feature importance methods have been developed, including permutation feature importance, which is based on the decrease in model performance and SHAP values [47], which are based on the magnitudes of feature attributions. To increase the interpretability of our prediction models, SHAP was employed to integrate feature importance. SHAP is a game theory approach and a local explanation to depict the feature’s importance. It has been adopted in some studies [48], [49], [50] to interpret machine learning models. The explanation model can be illustrated by the following equation [47]:

(5) $$g\left(z^{\prime }\right)=\emptyset_{0}+\sum_{i=1}^{M}\emptyset_{i}z_{i}^{\prime }$$

where $z^{\prime }\in {\left\{0,1\right\}}^{M}$, with 0 and 1 indicating the absence and presence of a feature, respectively. M represents the number of simplified input features. The Shapley value $\emptyset_{i}$, namely the contribution of feature i, is calculated as:

(6) $$\emptyset_{i}=\sum_{z^{\prime }\subseteq x^{\prime }}\frac{\left|z^{\prime }\right|!\left(M-\left|z^{\prime }\right|-i\right)!}{M!}\left[f_{x}\left(z^{\prime }\right)-f_{x}\left(z^{\prime }\backslash i\right)\right]$$

where $\left|z^{\prime }\right|$ is the number of non-zeros in $z^{\prime }$, $z^{\prime }\backslash i$ means $z^{\prime }$ without feature i, $f_{x}$ is the output of the model, and $x^{\prime }$ represents simplified inputs.

The SHAP typically evaluates each feature individually; however, in some cases, quantifying the effect of a group of features may be more informative. As mentioned above, the data are 15-residue sequences. In the feature extraction process, the residue at each position was encoded by a 20-dimensional BLOSUM log-odds vector [51]. After being encoded by the BLOSUM62 substitution matrix, the sequences were converted into 300-dimensional (15 × 20) vectors, with each element in a vector representing a feature. Because the amino acid residue at each position was encoded by a 20-dimensional vector, representing 20 features, these features were clustered as a feature group when performing SHAP analysis, representing a group of features related to specific residues. As a result, 15 feature groups were obtained, corresponding to each position of a 15-residue sequence.

## Results

### Kinase–substrate data collection and kinase classification at group and family levels

**Figure 2:**
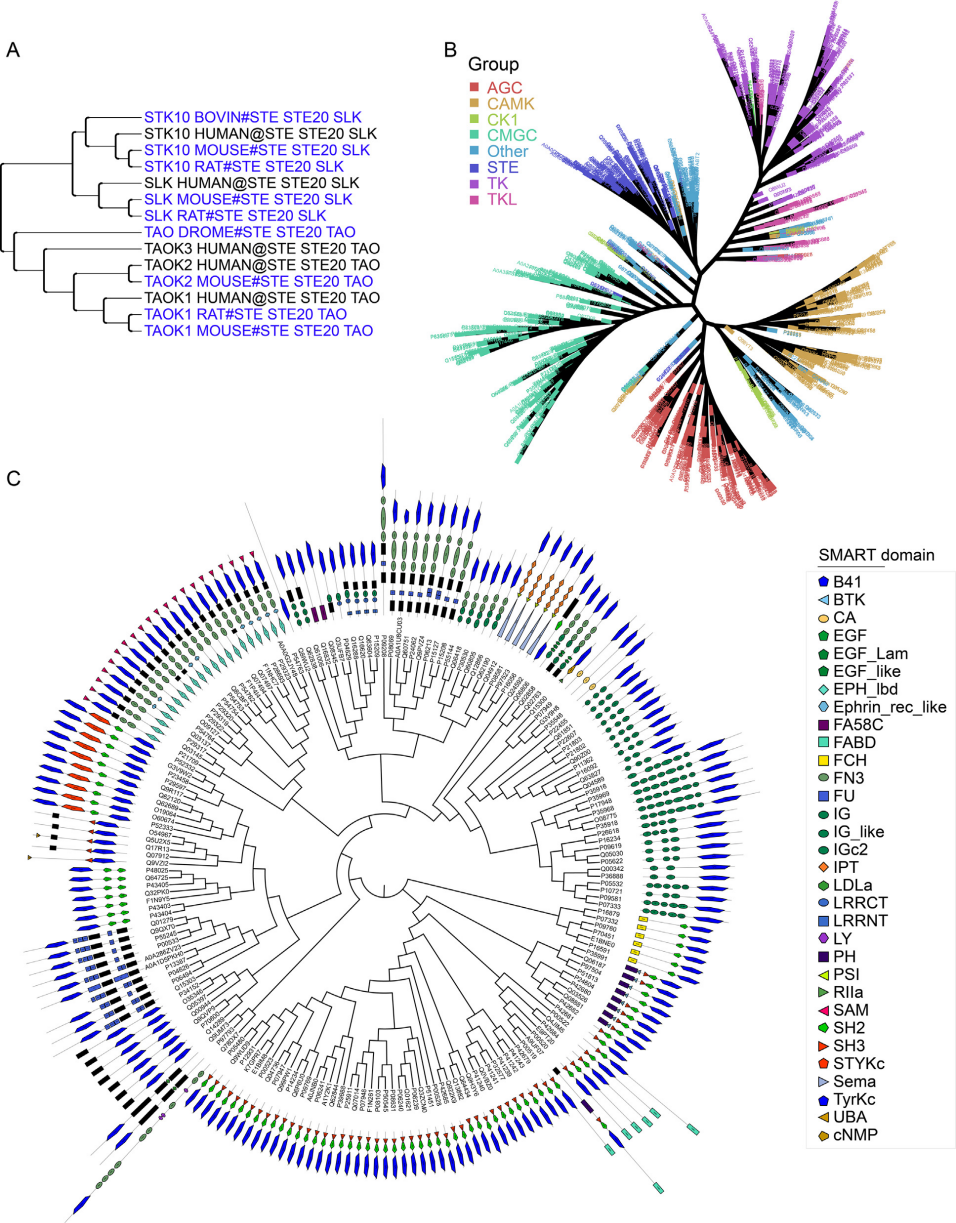
**Phylogenetic tree of the kinases of the SLK and TAO subfamilies in the STE20 family of the STE group** **A.** Phylogenetic tree of the kinases of the SLK and TAO, both of that are subfamilies in the STE20 family of the STE group. **B.** Kinome tree is composed of several major groups. **C.** Domain annotation of kinases in the TK group. SLK, STE20-like kinase; TAO, thousand-and-one; CAMK, calcium and calmodulin-regulated kinases; CK1, cell kinase 1; STE, sterile serine/threonine kinase; TK, thymidine kinase; TKL, tyrosine kinase-like; CDK, cyclin-dependent kinase; MAPK, mitogen-activated protein kinase; GSK, glycogen synthase kinase; CLK, cell division cycle-like kinase. AGC is named after the protein kinase A, G, and C familie kinases; CMGC is named after the initials of CDK, MAPK, GSK and CLK.

In total, we obtained 1380 unique kinases from the kinase–substrate dataset. Of these, 753 were included in the KinBase database, which includes classification information. In contrast, the remaining kinases needed to be annotated by other classification methods. Merging these kinases with the annotated dataset of the human kinome and classifying them by building an evolutionary phylogenetic tree (Figure S1) showed that proteins that are homologous or with consistent domains clustered tightly in smaller branches (Figure 2A), such as STK10_BOVIN, STK10_HUAMN, STK10_MOUSE, and STK10_RAT. Since STK10_HUAMN belongs to the STE20-like kinase (SLK) subfamily of the STE20 family of the sterile serine/threonine kinase (STE) group, we inferred that the other three kinases also belong to that subfamily. Different subfamilies of kinases can form different clusters. For example, for TAO_DROME, TAOK3_HUMAN, TAOK2_HUAMN, TAOK2_MOUSE, TAOK1_HUAMN, TAOK1_RAT, and TAOK1_MOUSE, although they also belong to the STE20 family of the STE group, the difference in the domain amino acid sequence from the SLK subfamily placed them on another branch belonging to the thousand-and-one (TAO) subfamily. Based on this process, we annotated the collected kinases to groups, families, and subfamilies. Figure 2B shows a kinome tree for several major groups. Analysis of each group separately showed that kinases in the same group contained similar domains (Figure 2C). This indicated that our annotation of the collected kinases was reliable.

Finally, these kinases were classified into 12 kinase groups and 116 kinase families. When we developed our predictive models, only groups or family clusters with at least 15 experimental phosphorylation sites were considered. As a result, ten groups and 81 families were retained. As serine/threonine (S/T) and tyrosine (Y) kinases modify different residues, we developed prediction models for both types separately in family clusters. Similarly, only group or family clusters with at least 15 related sites were considered. Since most substrate residues in the tyrosine kinase (TK) group were Y, while most substrate residues in the other nine groups were S/T, they were not separately considered when creating group prediction models. Moreover, we developed prediction models at the individual kinase level for clusters with more than 15 phosphorylation sites, with 11 types of organisms retained. While the majority are *Homo sapiens* (human), *Mus musculus* (mice), and *Rattus norvegicus* (rat), others include *Arabidopsis thaliana* (arath), *Bos taurus* (bovine), *Gallus gallus* (chicken), *Sus scrofa* (pig), *Pongo abelii* (ponab), *Schizosaccharomyces pombe* (schpo), *Xenopus laevis* (xenla), and yeast. Again, phosphoserine/phosphothreonine and phosphotyrosine sites were considered separately if their number in substrates of a particular kinase was no less than 15. In practice, 15-residue sequences (−7 to +7) surrounding kinase-specific phosphorylation sites were extracted as positive data. After removing redundant sites within each cluster, numbers of clusters and the number ranges for the positive data in each are summarized in Table 1. We obtained ten models for the ten group clusters; 81, 61, and 20 models were built for family clusters considering S/T and Y sites, S/T sites, and Y sites, respectively; 302, 243, and 54 models were developed for kinase-specific clusters considering S/T and Y sites, S/T sites, and Y sites, respectively. A total of 771 prediction models were created. In the group clusters, the numbers of positive sites ranged from 204 to 5737. In the family clusters, the numbers ranged from 15 to 2050, and the numbers of kinase clusters ranged from 15 to 930. Although clusters with positive sites of less than 15 were not considered when developing models, the data for these clusters are included in Table S1 for those who might be interested in them.

**Table 1:** **Number of prediction models and range of positive sites in each prediction model**

**Cluster**	**Model number**	**Number range of positive sites**
Group	10	204–5737
Family_all	81	15–2050
Family_ST	61	15–2046
Family_Y	20	18–1310
Kinase_all	302	15–930
Kinase_ST	243	15–929
Kinase_Y	54	15–652

*Note*: all indicate the Ser/Thr and Tyr sites, ST means Ser/Thr sites, and Y refers to Tyr sites.

### Pretreatment of negative datasets and characteristics of the positive 15-residue sequences with sequence logos

In each cluster, all the same types of residues in the phosphorylated substrate proteins, except those known to be positive phosphorylation sites, were regarded as negative data. For example, in family clusters considering all phosphorylated residues (model type Family_all listed in Table 1), all S/T and Y sites in all substrate proteins in a cluster were obtained. After eliminating the positive data (*i.e.*, experimentally verified phosphorylation sites), the remaining sites were taken as the negative data of that cluster. Similarly, in family clusters considering S/T residues (model type Family_ST in Table 1), the negative data are all S and T sites except those sites in the positive data for that cluster. CD-HIT [52] has been widely used to reduce sequence similarity in the literature [24], [25]. Because the number of negative sites obtained via this method is much greater than the number of positive sites, for balance we first used the CD-HIT-2D [52] to reduce the similarity of negative data to positive data with a similarity threshold of 0.4, the minimum threshold in the CD-HIT-2D suite. Furthermore, CD-HIT [52] was employed to further reduce the similarity between the negative data in each cluster. After experimentally applying different threshold values, we found that the number of negative sites is sometimes much greater than the number of positive sites, even though the minimum threshold of 0.4 in the CD-HIT suite was adopted. Suppose the number of negative sites is more than five times greater than the number of positive sites after applying CD-HIT-2D and CD-HIT. In this case, we applied the random undersampling technique from the imbalanced-learn library in Python to keep the number difference within five-fold to reduce the imbalance between positive and negative data when developing the predictive models.

**Figure 3:**
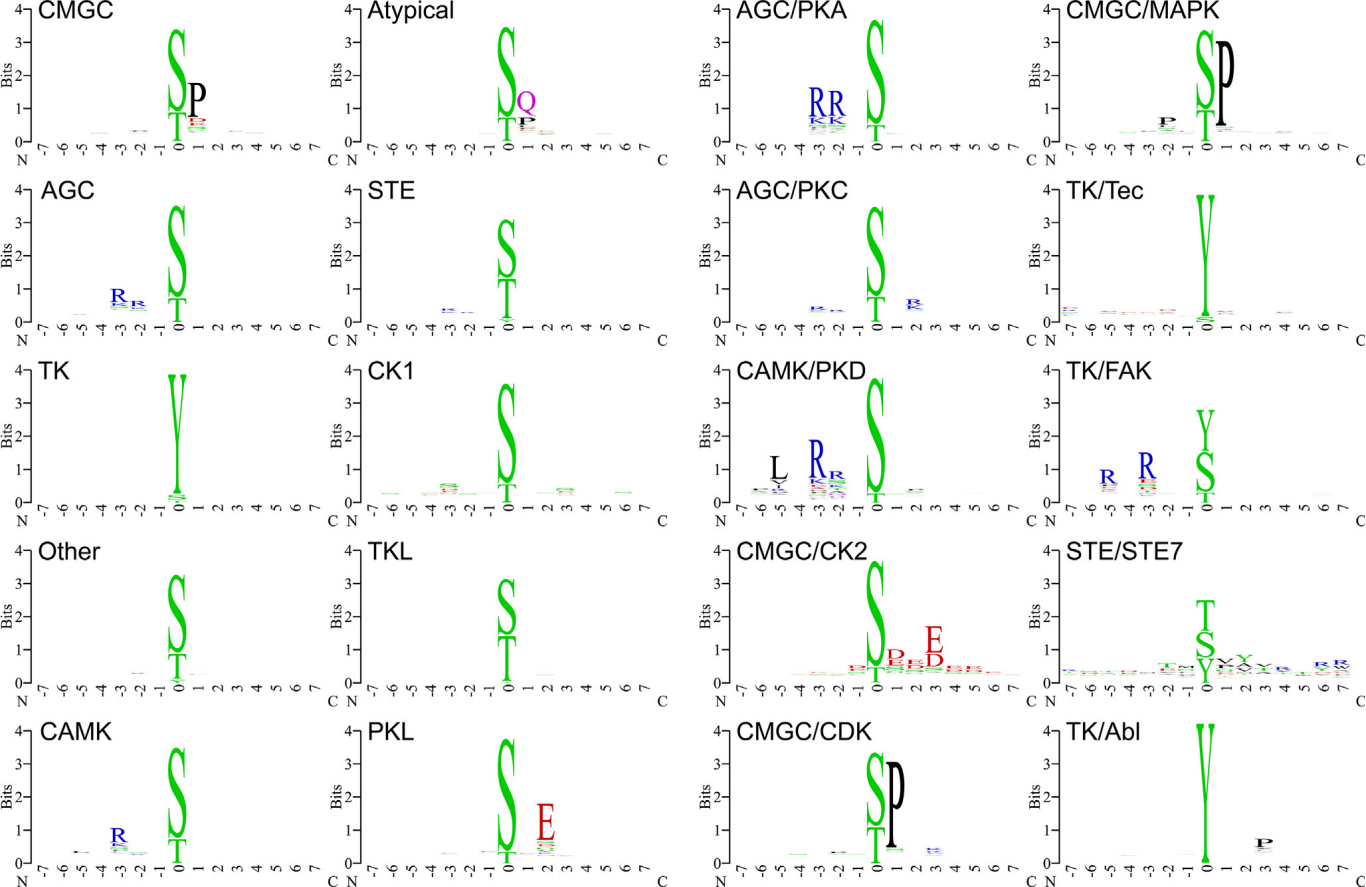
**Sequence logos of site clusters of different kinase groups and family clusters** Sequence logos of substrate site clusters phosphorylated by kinases from the CMGC, AGC, TK, Other, CAMK, Atypical, STE, CK1, TKL, and PKL, are shown in the left two columns. Those phosphorylated by kinases of the PKA, PKC, PKD, CK2, CDK, MAPK, Tec, FAK, STE7, and Abl, are shown in the right two columns. Atypical, atypical protein kinases; PKL, protein kinase-like; PKA, protein kinase A; PKC, protein kinase C; PKD, protein kinase D; CK2, casein kinase 2; CDK, cyclin-dependent kinase; MAPK, mitogen-activated protein kinase; Tec, tyrosine kinase; FAK, focal adhesion kinase; STE7, serine/threonine-protein kinase STE7; Abl, Abelson kinase.

To investigate the characteristics of amino acid composition in the aforementioned positive 15-residue sequences and provide a graphical representation, we obtained sequence logos of positive sequence clusters for all models using the WebLogo tool (https://weblogo.berkeley.edu/). Some representative logos are shown in Figure 3, which correspond to the ten groups (left two columns) and to some representative families (right two columns). In the common kinase family protein kinase A (PKA), protein kinase C (PKC), protein kinase D (PKD), casein kinase 2 (CK2), cyclin-dependent kinase (CDK), and mitogen-activated protein kinase (MAPK), the majority of phosphorylated sites are S/T residues, as shown in Figure 3. Kinases of some families, such as the focal adhesion kinase (FAK) and serine/threonine-protein kinase STE7 (STE7) families, can phosphorylate both S/T and Y residues. The Abelson kinase (Abl) family and tyrosine kinase (Tec) family clusters mainly correspond to Y sites. More sequence logos of these families and individual kinases are provided in Table S2.

### Performance of KinasePhos 3.0 and comparison with other tools to justify the effectiveness

As there are a total of 771 prediction models, to conveniently present their overall performance, average values of accuracy, weighted F1_score, weighted precision, weighted recall, and AUC for models in each of the seven types of clusters (that is, Group, Family_all, Family_ST, Family_Y, Kinase_all, Kinase_ST, and Kinase_Y, as presented in Table 1) were calculated (Table 2). The performance of each model is shown in Table S2. It is worth noting that the accuracy, weighted F1_score, weighted precision, weighted recall, and AUC were generally slightly higher with XGBoost than with SVM. Thus, the XGBoost algorithm was adopted for training our models to develop the website prediction function.

**Table 2:** **Performance comparison of KinasePhos 3.0 with SVM and XGBoost on selected metrics**

**Cluster**	**Model type**	**Accuracy**	**Weighted F1_score**	**Weighted precision**	**Weighted recall**	**AUC**
Group	SVM	0.847	0.832	0.850	0.847	0.888
	XGBoost	0.856	0.849	0.852	0.856	0.891
Family_all	SVM	0.873	0.833	0.827	0.873	0.828
	XGBoost	0.881	0.862	0.862	0.881	0.819
Family_ST	SVM	0.873	0.836	0.831	0.873	0.839
	XGBoost	0.883	0.866	0.866	0.883	0.836
Family_Y	SVM	0.832	0.791	0.803	0.832	0.826
	XGBoost	0.830	0.812	0.817	0.830	0.809
Kinase_all	SVM	0.857	0.602	0.774	0.857	0.808
	XGBoost	0.873	0.851	0.845	0.873	0.807
Kinase_ST	SVM	0.860	0.807	0.782	0.860	0.832
	XGBoost	0.881	0.863	0.860	0.881	0.830
Kinase_Y	SVM	0.816	0.747	0.711	0.816	0.746
	XGBoost	0.809	0.776	0.763	0.809	0.716

*Note*: The classification metrics listed here are the average of measures for all models belonging to that cluster. SVM, support vector machine; XGBoost, eXtreme Gradient Boosting algorithm; AUC, area under the receiver operating characteristic curve.

To examine these models in more detail, the classification performance of the kinase group models and the numbers of positive and negative sites used to train them are presented in Table 3.

**Table 3:** **Performance of kinase group XGBoost models with 10-fold cross-validation**

**Kinase** **group**	**No. of positive sites**	**No. of negative sites**	**Accuracy**	**Weighted F1_score**	**Weighted precision**	**Weighted recall**	**AUC**
CMGC	5737	1470	0.943	0.943	0.944	0.943	0.982
AGC	4602	1632	0.901	0.901	0.901	0.901	0.958
TK	2680	1688	0.808	0.807	0.808	0.808	0.884
Other	2068	1523	0.790	0.790	0.792	0.790	0.875
CAMK	1892	1920	0.852	0.852	0.854	0.852	0.928
Atypical	1037	2004	0.886	0.88	0.888	0.886	0.935
STE	625	1595	0.837	0.826	0.833	0.837	0.851
CK1	508	1402	0.857	0.849	0.854	0.857	0.888
TKL	360	1222	0.802	0.769	0.775	0.802	0.744
PKL	204	1400	0.882	0.875	0.876	0.882	0.862

*Note*: TK, tyrosine kinase; CAMK, calcium and calmodulin-regulated kinases; Atypical, atypical protein kinases; STE, sterile serine/threonine kinase; CK1, cell kinase 1; TKL, tyrosine kinase-like; PKL, protein kinase-like; CDK, cyclin-dependent kinase, MAPK, mitogen-activated protein kinase, GSK, glycogen synthase kinase, CLK, cell division cycle-like kinase; CMGC is named after the initials of CDK, MAPK, GSK and CLK; AGC, is named after the protein kinase A, G, C familie kinases.

**Figure 4:**
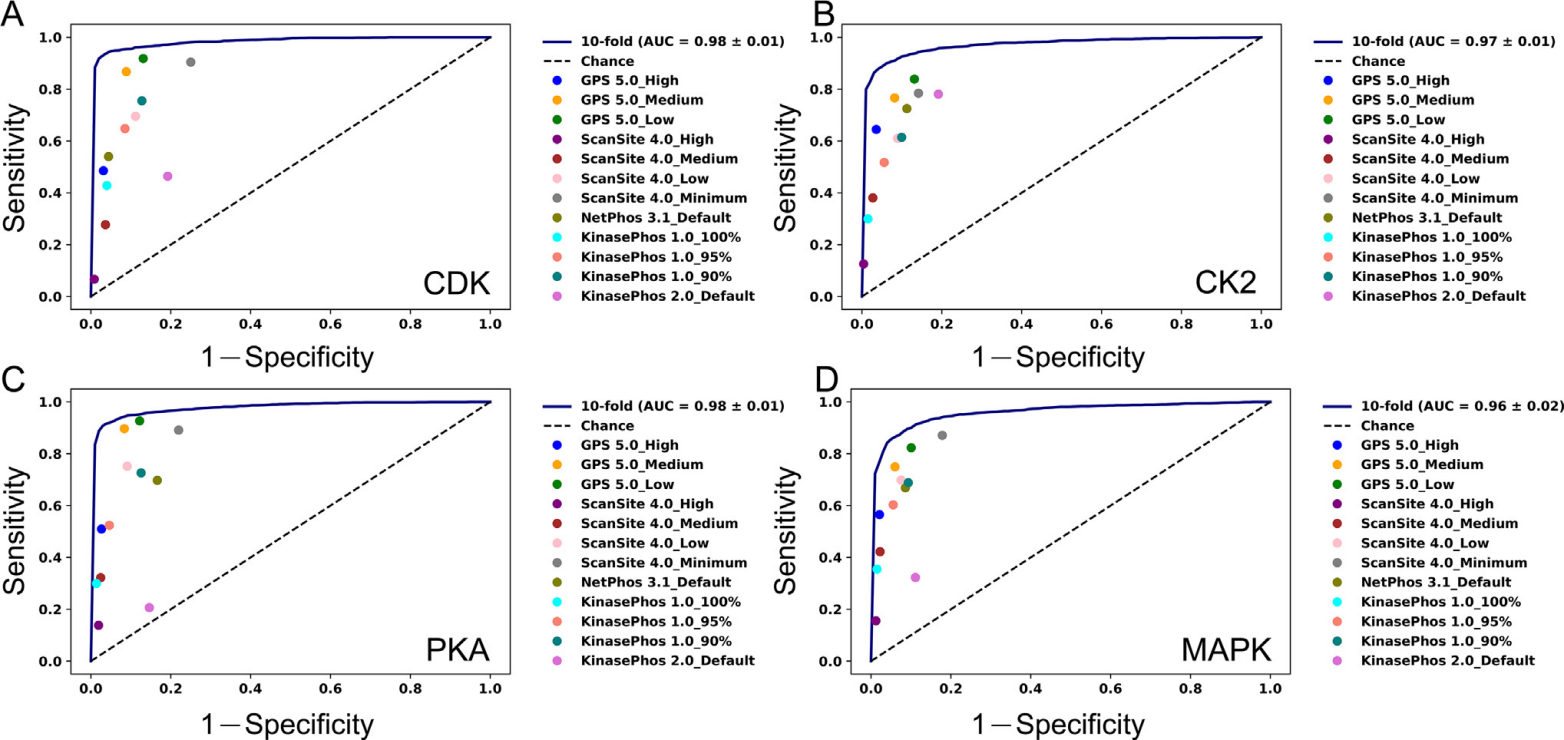
**Performance comparisons between KinasePhos 3.0 and existing tools** **A.** The ROC curve of the CDK model and its comparison to some existing tools. **B.** The ROC curve of the CK2 model and its comparison to some existing tools. **C.** The ROC curve of the PKA model and its comparison to some existing tools. **D.** The ROC curve of the MAPK model and its comparison to some existing tools. Existing tools include GPS 5.0 (blue, orange, and green dots), Scansite 4.0 (purple, brown, pink, and grey dots), NetPhos 3.1 (olive dot), KinasePhos 1.0 (cyan, salmon, and teal dots), and KinasePhos 2.0 (orchid dot). ROC, receiver operating characteristic curve; AUC, area under the ROC curve; GPS 5.0, the Group-based Prediction System (GPS) 5.0.

The new KinasePhos 3.0 was compared with other predictive models, namely KinasePhos 1.0 [32], KinasePhos 2.0 [33], GPS 5.0 [29], Scansite 4.0 [53], and NetPhos 3.1 [26], using four typical kinase families (CDK, CK2, PKA, and MAPK). We found that KinasePhos 3.0 is competitive (Figure 4). ROC curves produced by 10-fold cross-validation of KinasePhos 3.0 are presented, with the sensitivity (Sn) and 1−Specificity (Sp) values for the other tools shown as dots with different colors in the plots.

**Figure 5:**
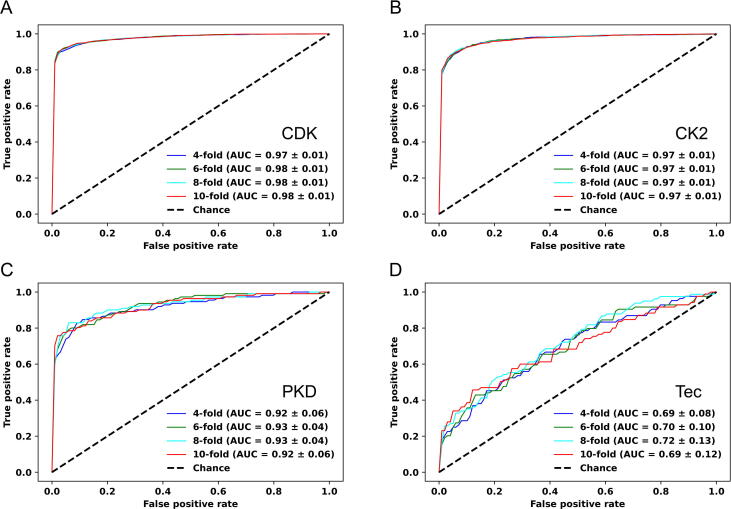
**Performance comparisons between KinasePhos 3.0 at different levels of cross-validation** **A**. Performance comparison of CDK family model resulting from 4-, 6-, 8-, and 10-fold cross-validations. **B**. Performance comparison of CK2 family model resulting from 4-, 6-, 8-, and 10-fold cross-validations. **C**. Performance comparison of PKD family model resulting from 4-, 6-, 8-, and 10-fold cross-validations. **D**. Performance comparison of Tec family model resulting from 4-, 6-, 8-, and 10-fold cross-validations.

When *k*-fold cross-validation was applied, an optimization investigation of *k* for cross-validation with *k* = 4, 6, 8, and 10 was performed (Figure 5), which includes the CDK, CK2, PKD, and Tec families and compares the performance with ROC curves and AUC values. We found that the selection of k did not have a significant impact on performance; thus, the commonly used 10-fold cross-validation was adopted for presenting performance.

Human Beclin-1 (UniProtKB ID: Q14457) has been used as a test protein in GPS 5.0 to predict kinase-specific phosphorylation sites. For comparison, we used it to investigate the predictions made by KinasePhos 3.0. Models in the kinase group named after the protein kinase A, G, C families (AGC) were selected as representative models. GPS 5.0 predicted 38, 49, and 56 phosphorylation sites with high, medium, and low thresholds, respectively, while KinasePhos 3.0 obtained 33 phosphorylation sites. It should be noted that all these 33 phosphorylation sites lie within the 56 phosphorylation sites predicted by GPS 5.0 with a low threshold. Of these 33 phosphorylation sites, 30 belong to the 49 phosphorylation sites predicted by GPS 5.0 with a medium threshold, and 25 of these 33 phosphorylation sites fall among the 38 phosphorylation sites predicted by GPS 5.0 with a high threshold. Therefore, the prediction results from KinasePhos 3.0 are reasonably consistent with GPS 5.0.

### Interpretation of model features with SHAP values to link the potential roles of surrounding residues in phosphorylation

We used MAPK1 (UniProtKB ID P28482) of *Homo sapiens* to test the protein kinase B (Akt) family prediction model. MAPK1 is a serine/threonine kinase that plays an essential role in the MAPK signal transduction pathway. Notably, residues 29, 185, 187, 190, 246, 248, and 284 in MAPK1 can be phosphorylated [35]. To further investigate the importance of feature groups to amino acid characteristics of these 15-residue sequences, iceLogo [54] (https://iomics.ugent.be/icelogoserver/create), which is a web-based service capable of visualizing conserved patterns in protein and nucleotide sequences with probability theory, was used to obtain sequence logos to compare the difference between positive and negative data belonging to the same clusters.

**Figure 6:**
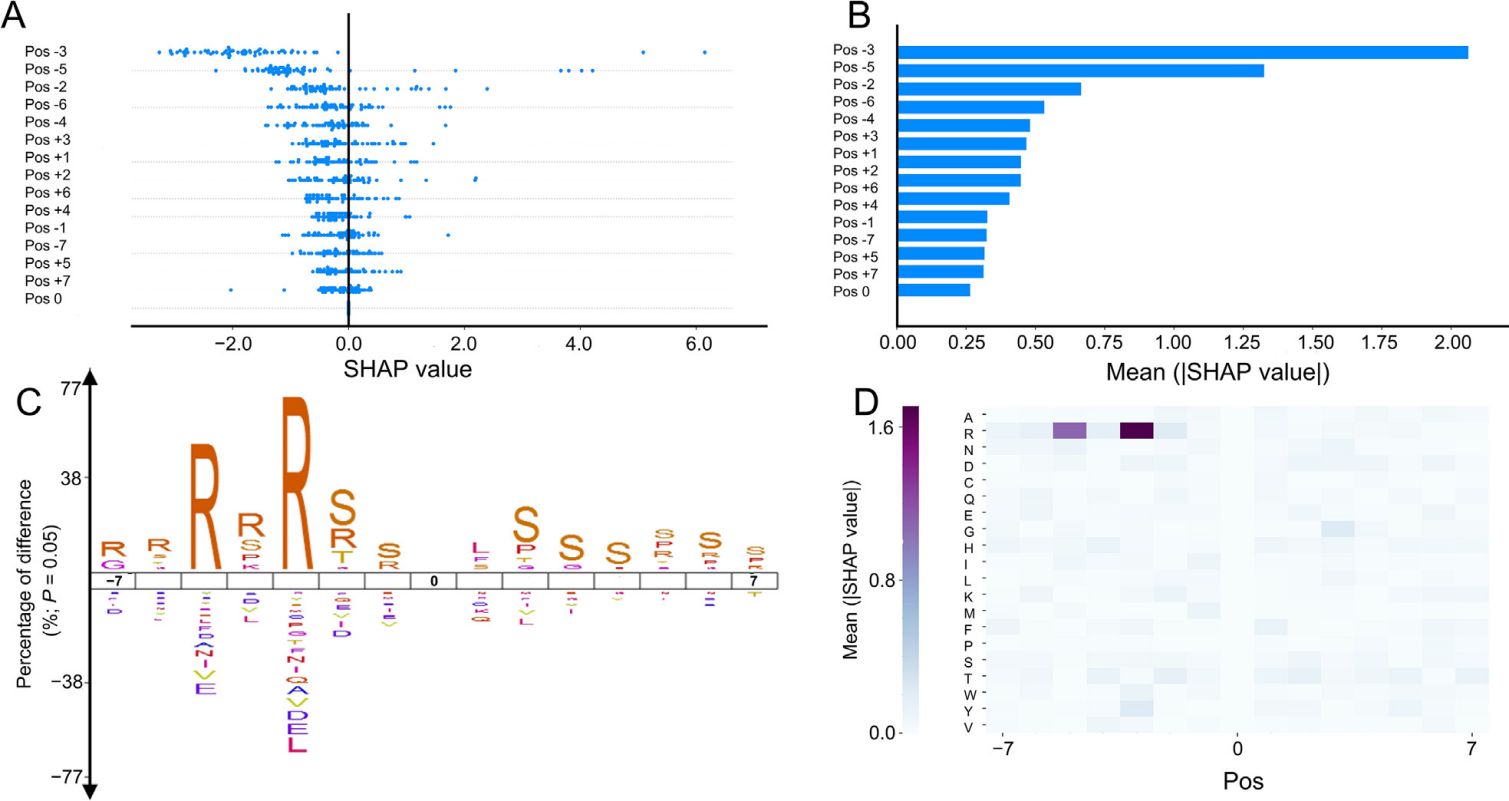
**Feature explained by SHAP values** **A.** SHAP values showing the impacts of feature groups on model output. **B.** Mean absolute SHAP values demonstrating the average impact of feature groups on model output magnitude. **C.** iceLogo of Akt family positive phosphorylation sites contrasted with its negative sites. **D.** Heatmap of mean absolute SHAP values. Results are derived from using MAPK1 protein to test the Akt family prediction model. Akt, protein kinase B; SHAP, SHapley Additive exPlanations. Pos, position.

Figure 6A and B represent the impact of feature groups on model output, while Figure 6C shows the iceLgo of the positive phosphorylation sites of the Akt family in contrast to the negative data. Figure 6D shows a heat map of the mean absolute SHAP values to show the impact of the features on the model output magnitude. It can be observed that the third position (pos−3) and fifth position (pos−5) before the phosphorylated sites have a relatively significant negative impact on the model prediction results. The results computed from SHAP are consistent with the iceLogo sequence and also with the position weight values computed for the Akt family at positions −5 and −3 in GPS 5.0, which were 0.85 and 1.00, respectively [22].

### Web interface and downloadable tool for the prediction of kinase-specific phosphorylation sites

The KinasePhos3.0 prediction service can be accessed via a web interface and a standalone prediction tool, the usages of which are presented in this section. MAPK1 (UniProtKB ID: P28482) and human Beclin-1 (UniProtKB ID: Q14457) were selected to illustrate the prediction of kinase-specific phosphorylation sites.

#### Web interface

**Supplementary Figure S2:**
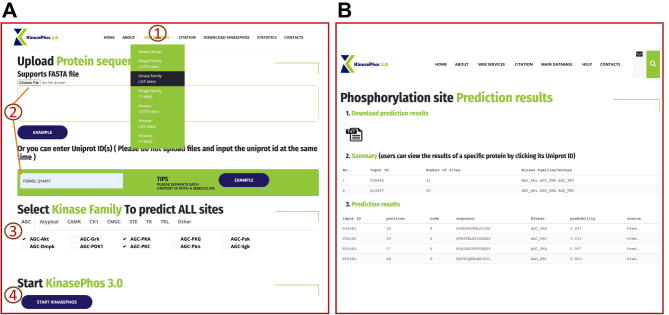
**The web interface of KinasePhos 3.0 A.** The web interface for users to select model types, upload or enter their data, and choose prediction models. **B.** The overview of predicted results.

The core parts of the web interface allowing users to upload data, choose kinase models, and view predictions are presented in Figure S2A and B. The navigation bar “WEB SERVICES” (① in Figure S2A) allows users to choose models of a specific type for the seven cluster types: group clusters considering S/T and Y sites; family clusters considering S/T and Y sites, S/T sites, and Y sites; and kinase-specific clusters considering S/T and Y sites, S/T sites, and Y sites. After choosing the model type, users can upload their FASTA format sequence data by clicking the “Choose File” button (② in Figure S2A). Alternatively, users can enter protein UniProtKB IDs in the box (② in Figure S2A) separated by a semicolon. Users can then choose models by ticking checkboxes (③ in Figure S2A). It should be noted that these models are sub-clustered into ten kinase groups: “AGC”, “atypical protein kinases (Atypical)”, “calcium and calmodulin-regulated kinases (CAMK)”, “cell kinase 1 (CK1)”, “kinase group named after the initials of CDK, MAPK, glycogen synthase kinase (GSK) and cell division cycle-like kinase (CLK) (CMGC)”, “other kinases (Other)”, “protein kinase-like (PKL)”, “STE”, “TK“, and “tyrosine kinase-like (TKL)”, so users can click a specific kinase group first and then check the models belonging to that group. Subsequently, the users can click the “START KINASEPHOS” button (④ in Figure S2A) to run the prediction, following which, the result page, shown in Figure S2B, will finally appear.

**Figure 7:**
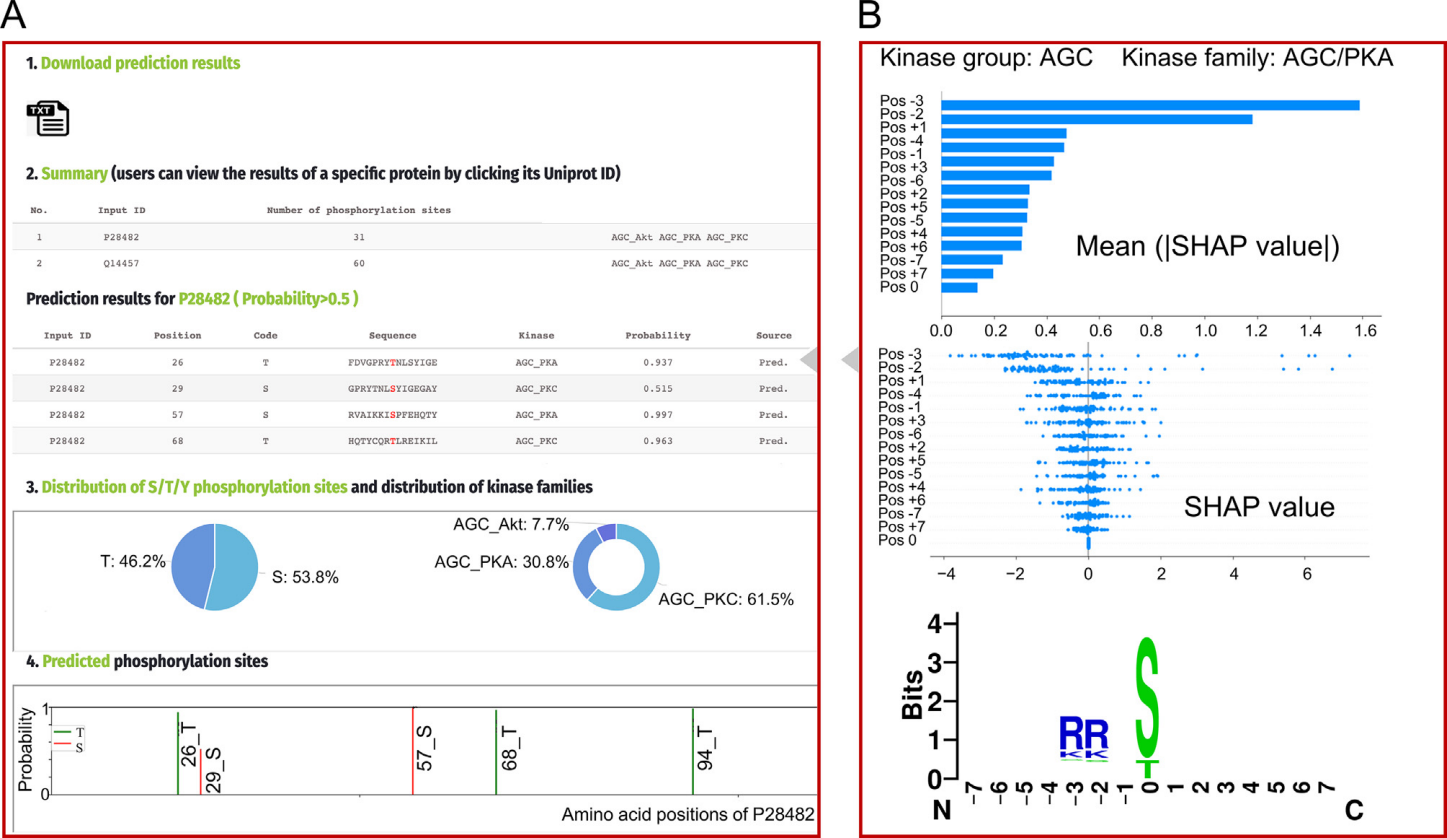
**The web interface showing the results related to a specific protein** **A.** Detailed predictions for a specific protein with predicted phosphorylation sites listed and depicted. **B.** SHAP showing the impacts of feature groups on model output and sequence logo of the corresponding model.

As shown in section 1 of Figure S2B, users can download prediction results in TXT format by clicking the download button. Section 2 summarizes the proteins uploaded or entered by users, along with the numbers of predicted phosphorylation sites for each protein and the models chosen by users. If a UniProtKB ID entered by users does not match any IDs in the UniProtKB database, it will be ignored. Section 3 lists the predicted sites. Similar to GPS 5.0, a column called “Source” is used to indicate whether the phosphorylation site has been experimentally verified (Exp.) or merely predicted (Pred.). To view the details for a specific protein, users can click the UniProtKB ID in column “Input ID” of section 2, and the page shown in Figure 7 will appear.

Section 2 in Figure 7A lists the predicted sites for a particular protein (P28482 was clicked in this example). When users mouse over the rows of this table, the window shown by Figure 7B will be displayed on the right-hand side, showing the impact of feature groups on model output, (Figure 6A and B), and the sequence logo of the corresponding model. The distribution of S/T/Y phosphorylation sites and the distribution of the models are presented in the pie charts in Section 3. To provide a more intuitive view of the predicted phosphorylation sites, Section 4 displays predicted sites in a figure with probabilities, with S, T, and Y sites labeled in different colors. If users want to switch to predictions for another protein, they can click the protein’s ID in Section 2.

#### Downloadable prediction tool

**Supplementary Figure S3:**
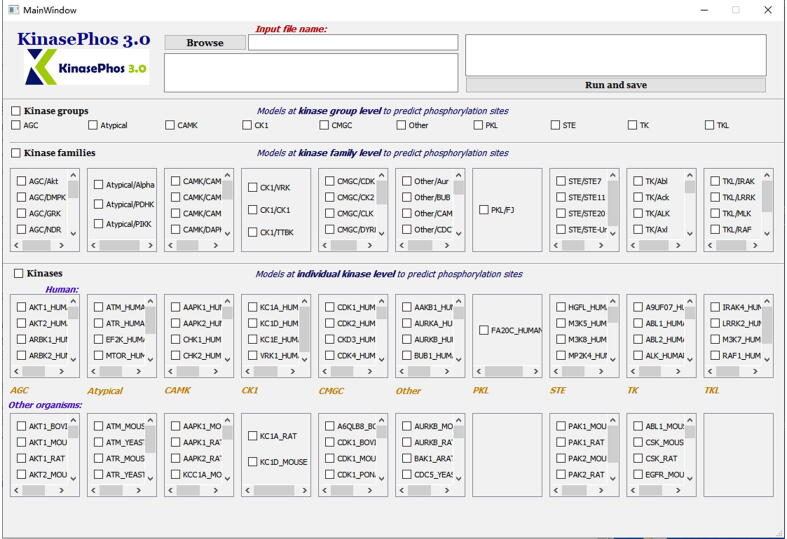
**The downloadable prediction tool** This is the standalone prediction tool of the KinasePhos3.0, which can predict S/T and Y phosphorylation sites at kinase group, kinase family, and individual kinase levels.

Considering the availability of large-scale phosphoproteomic data, a downloadable prediction tool (as shown in Figure S3) to predict all S/T and Y phosphorylation sites at kinase group, kinase family, and individual kinase levels is also provided at https://awi.cuhk.edu.cn/KinasePhos/download.html and https://github.com/tom-209/KinasePhos-3.0-executable-file. After downloading and starting KinasePhos3.exe, the “Browse” button is used to upload the data file, which should be a text file in FASTA format, as shown in the “Example Input.txt” file that is downloaded along with the tool. Users can then choose prediction models to test their data using checkboxes. If the “Kinase groups” are checked, all models at the group level will be executed. In addition, users can also choose group models separately by ticking the “AGC”, “Atypical”, “CAMK”, “CK1”, “CMGC”, “Other”, “PKL”, “STE”, “TK”, or “TKL” checkboxes based on their requirements. Similarly, users can test their data using all models at the family and individual kinase levels by checking the “Kinase families” and the “Kinases” checkboxes, respectively. Additionally, users can choose a specific family model or kinase model by clicking the corresponding checkboxes. It should be noted that these models at the kinase family level and individual kinase level are grouped into ten scroll areas corresponding to the ten kinase groups, while the models at the individual kinase level are further classified into human and other organisms for the convenience of testing data from humans and other species. With the models checked, users then click “Run and save” to run the prediction tool and save prediction results. It will take some time if users select many models and submit large-scale data before it produces a window that allows users to specify a location to save results as a comma-separated values (CSV) file. This downloadable prediction tool is recommended for users who want to test large-scale data using our predictive models.

## Discussion

Although advances in mass spectrometry and enrichment methods have led to a massive increase in high-throughput phosphoproteomic data, it is still difficult to determine the number of phosphorylation sites that can exist in a eukaryotic proteome [55]. Vlastaridis et al. estimated that there are 230,000, 156,000, and 40,000 phosphorylation sites in humans, mice, and yeast, respectively [55]. However, as noted above, we only identified 41,421 experimentally verified, kinase-specific phosphorylation sites from 135 organisms, even with data that are already more comprehensive than those included in previous tools. The numbers of experimentally verified, kinase-specific phosphorylation sites in humans, mice, and yeast identified in this study were 19,123, 4,618, and 332, respectively. Therefore, for most phosphorylation sites, the kinases that phosphorylate them are yet to be identified. Computational methods are viable solutions for kinase-specific phosphorylation prediction, as empirical methods are more time-consuming and expensive. Kinase-specific phosphorylation sites in the kinase family and individual kinase levels are divided into S/T and Y, S/T, and Y site clusters, a total of 771 clusters, with a prediction model created for each.

The performance of KinasePhos 3.0 is competitive with other existing kinase-specific phosphorylation site prediction tools, such as GPS 5.0 and Scansite 4.0. It should be highlighted that the kinase-specific phosphorylation sites employed to develop KinasePhos 3.0 are more comprehensive than those employed with the existing tools, which is illustrated by the number of sites presented. In addition to collecting data from other existing tools, we performed text mining for experimentally verified kinase-specific phosphorylation sites from the UniProtKB. The sample size is one of the most important parameters influencing model performance when developing machine learning-based classification models. We only used clusters with at least 15 experimentally verified phosphorylation sites when building models. This ensured that our sample size was comparable to those of some tools. For example, KinasePhos 2.0 used clusters with at least ten experimentally verified phosphorylation sites. In GPS 5.0, clusters with no less than three sites were considered, with 10-fold cross-validation and leave-one-out validation methods tested separately, to evaluate the predictors’ performance with 245 kinase categories with no less than 30 sites and 372 kinase categories with 3 to 30 sites, respectively.

KinasePhos 3.0 also offers SHAP feature importance when performing prediction tasks. Since features were grouped based on their positions in the peptides containing 15 amino acids, that is, features related to a specific position were regarded as a feature group. Feature group importance provides a more intuitive understanding of the implications of the surrounding residues on the phosphorylation of each peptide. The importance of the SHAP feature group is consistent with the sequence logo characteristics obtained from iceLogo, as illustrated above. The feature group importance is also consistent with the position weight computed using GPS 5.0. Instead of simply providing a prediction of whether a given residue can be phosphorylated by a specific kinase group, kinase family, or kinase, the inclusion of feature interpretation in the prediction models provides more insights into the potential roles of surrounding residues in phosphorylation.

Our study has several limitations. First, although we have collected a more comprehensive, experimentally verified kinase-specific phosphorylation site database than those used in other studies in this field, small numbers of these sites cannot be used to develop predictive models at the family or individual kinase level, as the number of sites is less than 15, below the threshold for creating a model, owing to data availability. However, these sites are included in the supplementary material for readers who might be interested in them. For example, the transfer learning technique adopted by Deznabi et al. [56] might be employed to predict phosphorylation sites for kinases with less than 15 known phosphorylation sites. Moreover, considering protein–protein interactions and structural characteristics of proteins might improve predictions for kinases with few known phosphorylation sites. Second, we did not investigate deep learning methods, some of which have been described in the literature, such as DeepPhos [57] and MusiteDeep [58], and have demonstrated effectiveness in predicting kinase-specific phosphorylation sites. Leveraging the power of deep learning, along with more features, will be a good strategy to explore in the future to further increase the prediction performance. Third, our models do not distinguish among organisms, although the majority of phosphorylation sites are from humans, mice, and rats. Tools that can separate species may better satisfy some users’ requirements. Fourth, there is room for improvement of the website. As part of future work, we will try to set different decision threshold values, allowing users to achieve higher precision or higher recall based on whether their requirements are precision-oriented or recall-oriented, respectively. Fifth, the standalone tool is available to the Windows system at this stage. However, we will try to make it available to other operating systems in the future.

## Conclusion

In conclusion, our updated KinasePhos 3.0 represents a significant improvement over versions 1.0 and 2.0. Notably, more comprehensive experimentally verified kinase-specific phosphorylation site data have been collected, and prediction models have been increased, with the potential to meet more specific requirements of the users. The prediction performance of this version is competitive with that of other existing tools, such as GPS 5.0. Importantly, we provide users with both web-based and downloadable tools, making it more user-friendly. In the future, KinasePhos 3.0 will be valuable for predicting unknown sites, judging if these sites can be phosphorylated by a specific kinase group, kinase family, or kinase, based on user requirements. These predictions will aid in empirical kinase and substrate characterization, reducing costs and saving time.

## Code availability

The source code is available on Github (https://github.com/tom-209/KinasePhos-3.0-executable-file) and BioCode (https://ngdc.cncb.ac.cn/biocode/tools/BT007291).

## Competing interests

The authors have declared no competing interests.

### CRediT authorship contribution statement

**Renfei Ma:** Conceptualization, Data curation, Methodology, Formal analysis, Investigation, Software, Visualization, Writing – original draft. **Shangfu Li:** Conceptualization, Data curation, Formal analysis, Writing – original draft. **Wenshuo Li:** Software, Visualization. **Lantian Yao:** Software. **Hsien-Da Huang:** Project administration, Conceptualization, Supervision, Funding acquisition, Writing – review & editing. **Tzong-Yi Lee:** Project administration, Conceptualization, Supervision, Funding acquisition, Writing – review & editing. All authors read and approved the final manuscript.

## Supplementary material

Supplementary Figure S1 **The evolutionary tree for all the collected kinases** The obtained evolutionary tree showed that homologous proteins or proteins with consistent domains clustered tightly in smaller branches.

Supplementary Table S1 **The experimentally identified kinase-specific phosphorylation sites used in KinasePhos3.0**

Supplementary Table S2 **The performance of the 771 models**
