# Supplementary material for: KinasePhos 3.0: Redesign and Expansion of the Prediction on Kinase-specific Phosphorylation Sites
Source: Genomics Proteomics Bioinformatics. 2022 Jul 1;21(1):228–41. doi: 10.1016/j.gpb.2022.06.004 (PMC10373160; doi:10.1016/j.gpb.2022.06.004)
Supplement: Supplementary Table S2 — The performance of the 771 models [file mmc3.docx]

**Table S2 The performance of the 771 models**

| **Clusters** | **No. of positive sites** | **Accuracy** | **Weighted F1 score** | **Weighted precision** | **Weighted recall** | **AUC** | **Logo** | **Mean \|SHAP value\|** | **SHAP value** |
| --- | --- | --- | --- | --- | --- | --- | --- | --- | --- |
| AGC (STY) | 4602 | 0.901 | 0.901 | 0.901 | 0.901 | 0.958 | 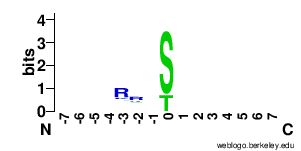 | 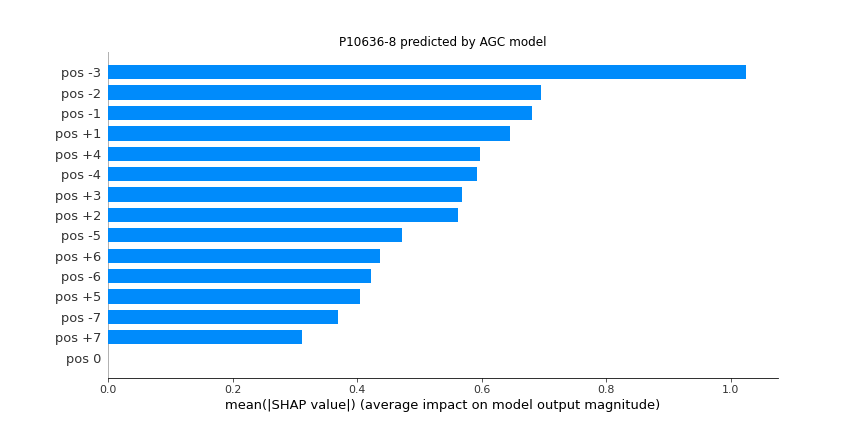 | 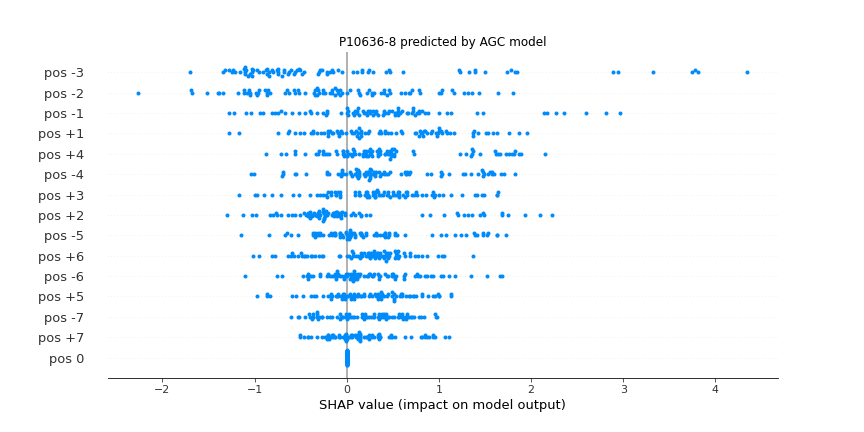 |
| Atypical (STY) | 1037 | 0.886 | 0.88 | 0.888 | 0.886 | 0.935 | 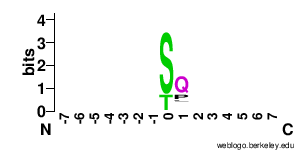 | 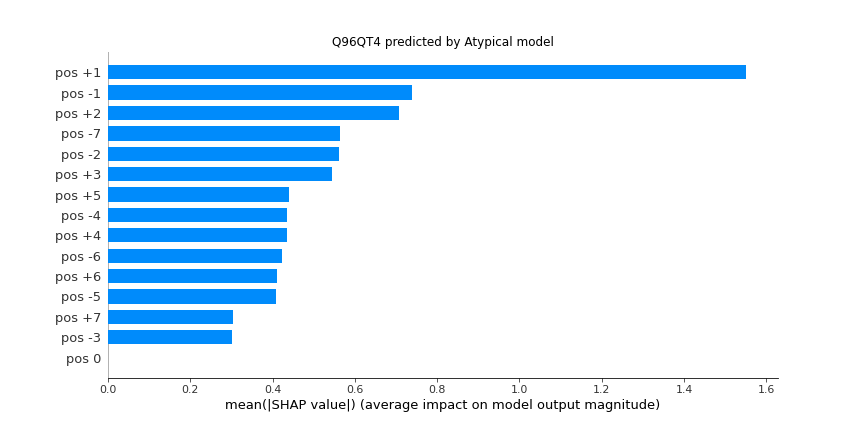 | 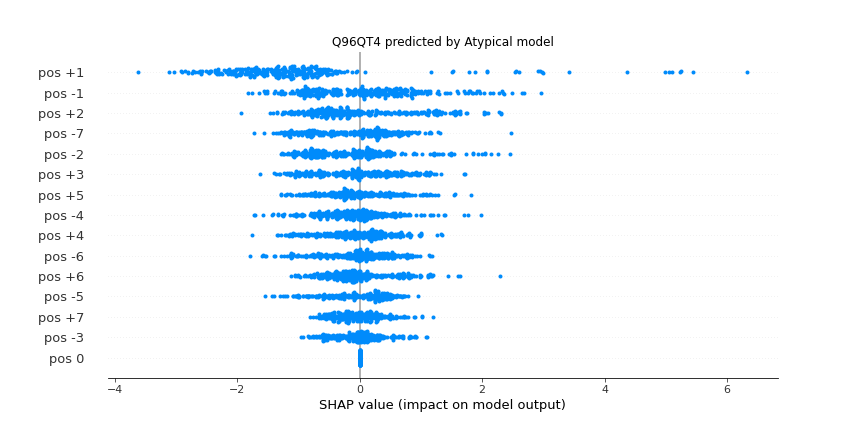 |
| CAMK (STY) | 1892 | 0.852 | 0.852 | 0.854 | 0.852 | 0.928 | 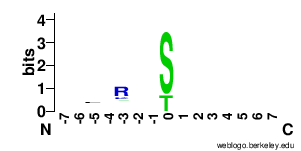 | 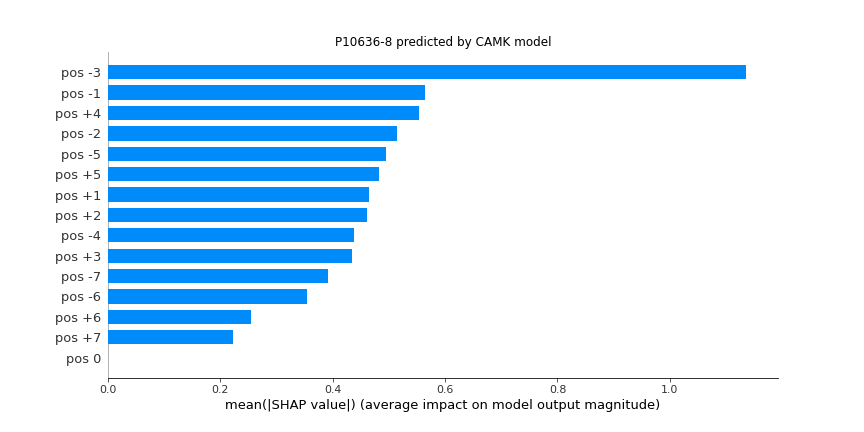 | 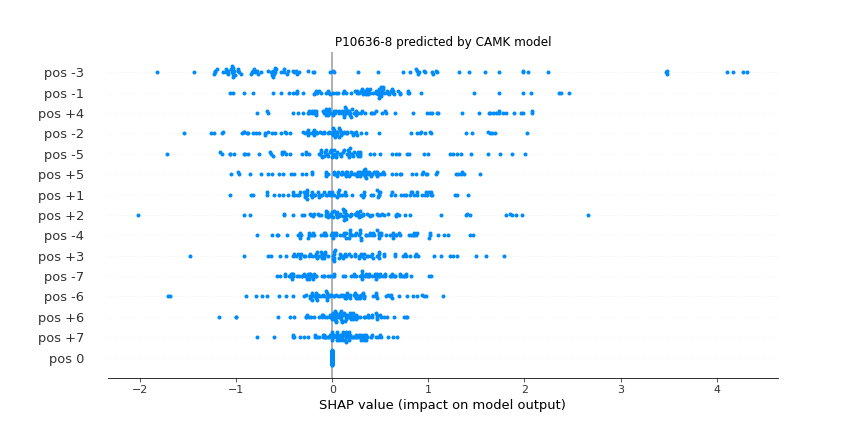 |
| CK1 (STY) | 508 | 0.857 | 0.849 | 0.854 | 0.857 | 0.888 | 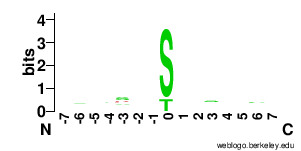 | 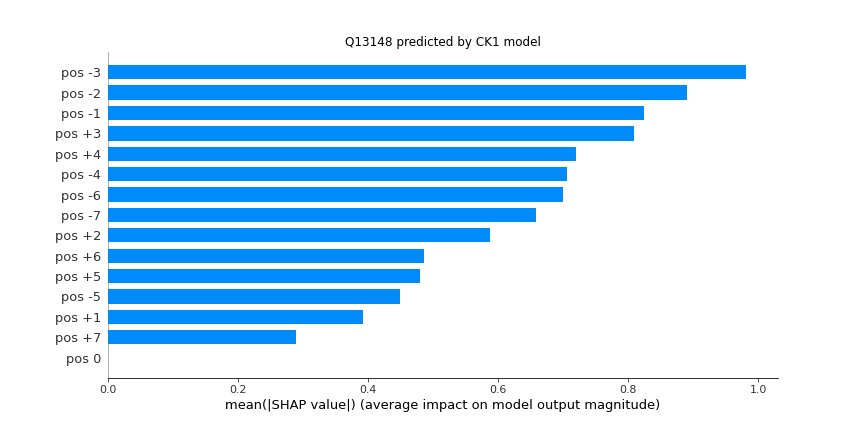 | 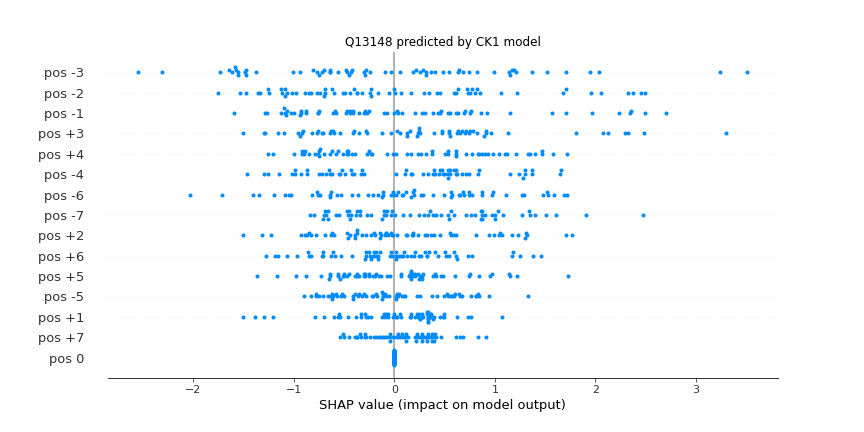 |
| CMGC (STY) | 5737 | 0.943 | 0.943 | 0.944 | 0.943 | 0.982 | 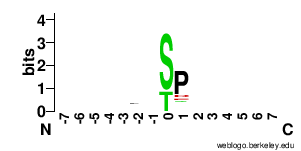 | 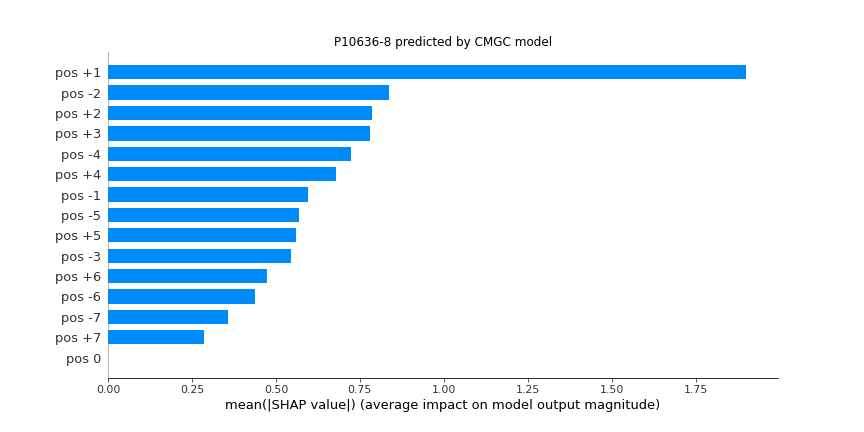 | 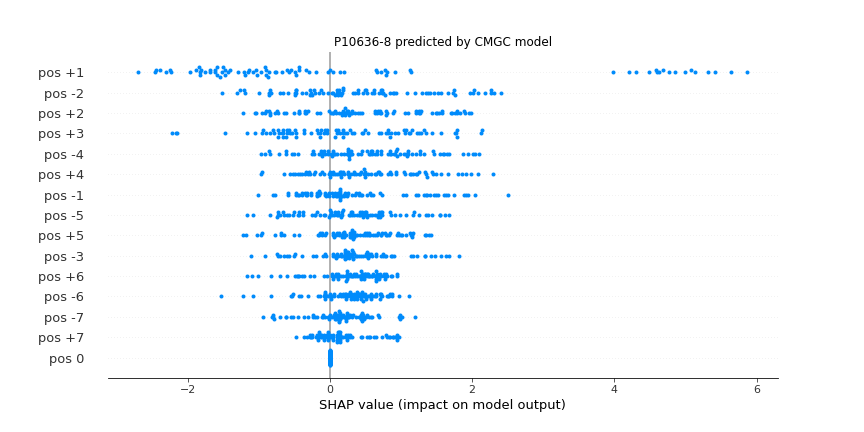 |
| Other (STY) | 2068 | 0.79 | 0.79 | 0.792 | 0.79 | 0.875 | 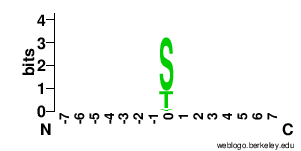 | 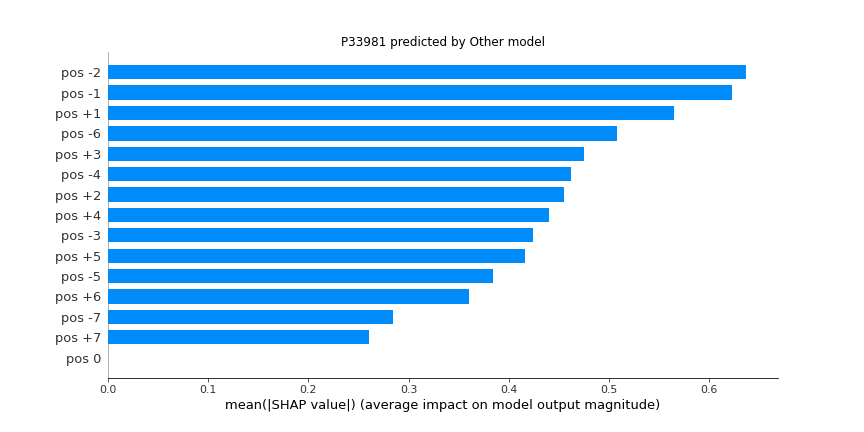 | 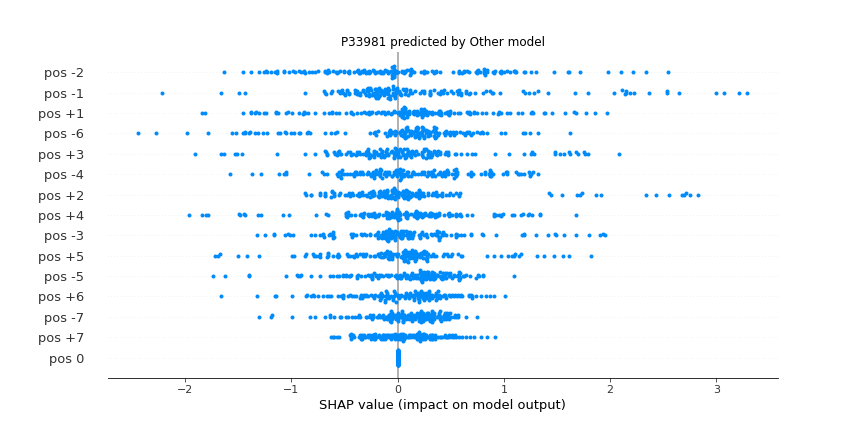 |
| PKL (STY) | 204 | 0.882 | 0.875 | 0.876 | 0.882 | 0.862 | 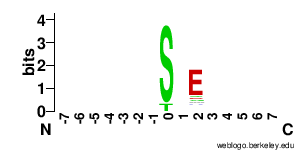 | 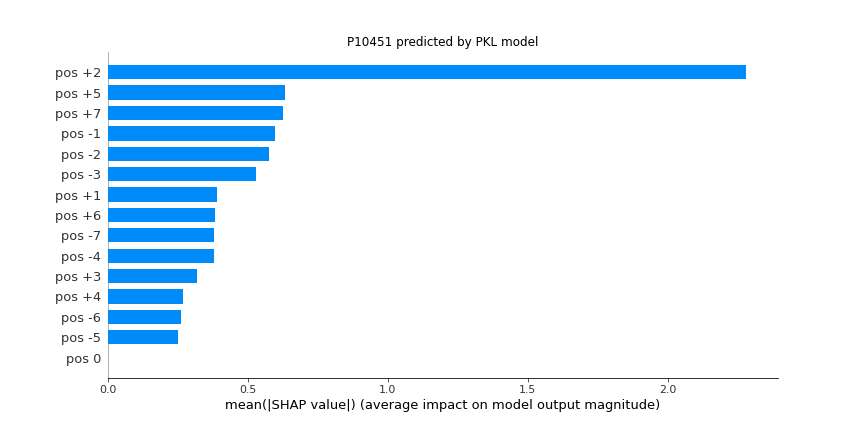 | 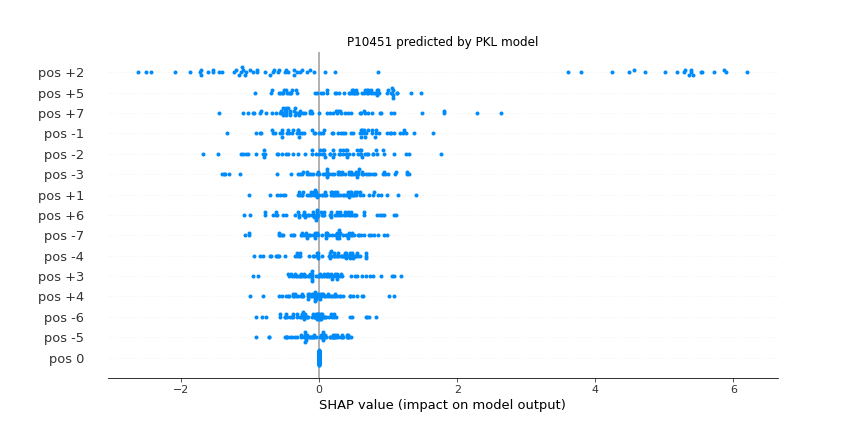 |
| STE (STY) | 625 | 0.837 | 0.826 | 0.833 | 0.837 | 0.851 | 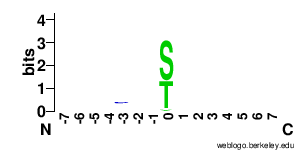 | 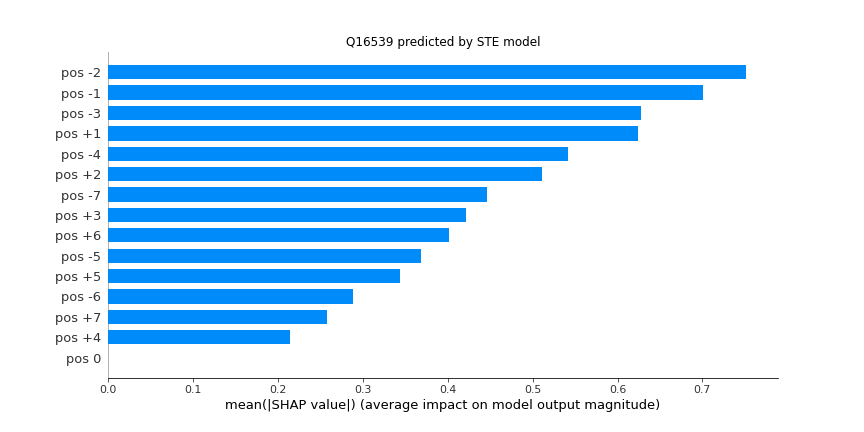 | 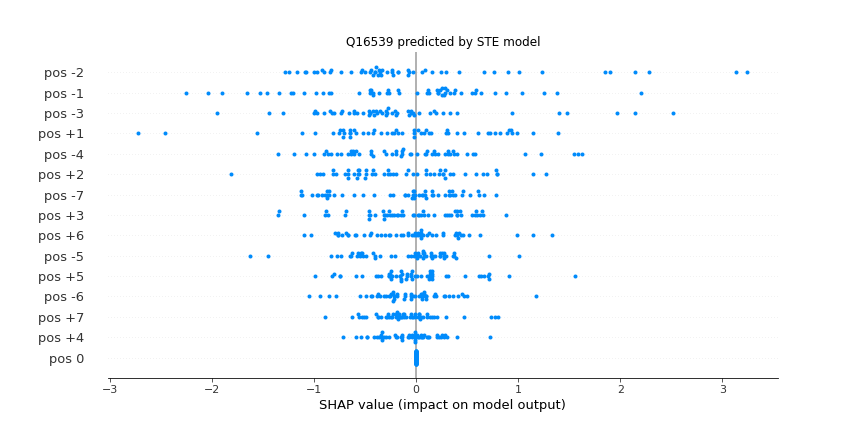 |
| TK (STY) | 2680 | 0.808 | 0.807 | 0.808 | 0.808 | 0.884 | 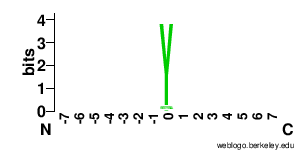 | 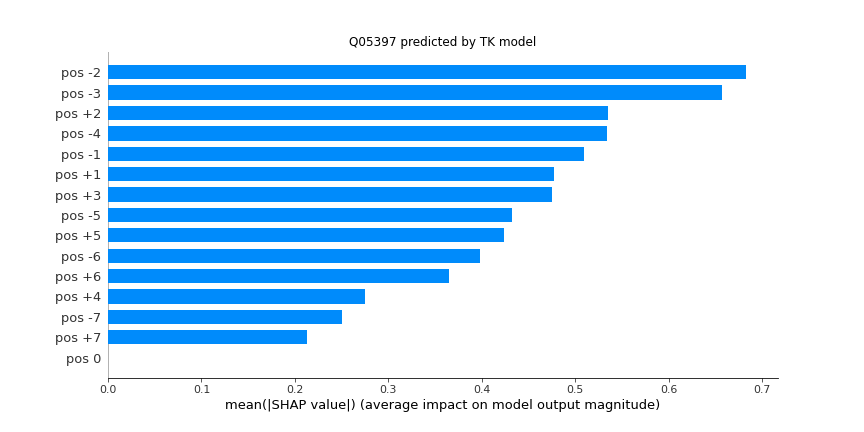 | 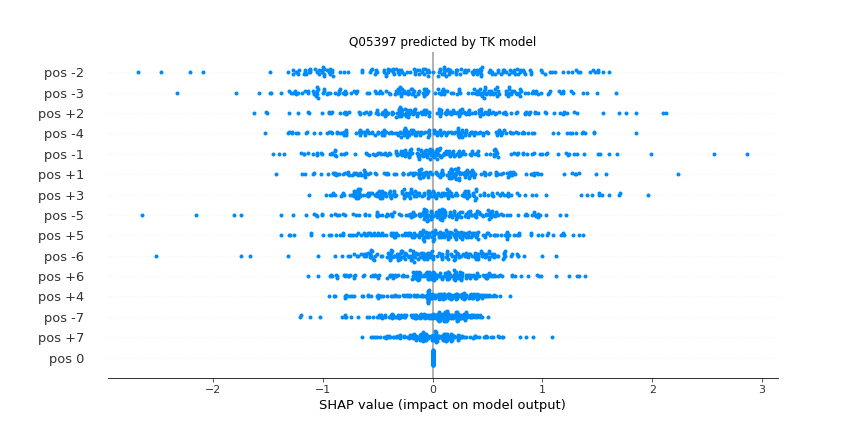 |
| TKL (STY) | 360 | 0.802 | 0.769 | 0.775 | 0.802 | 0.744 | 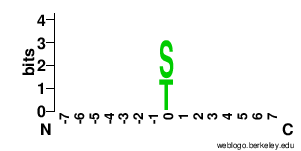 | 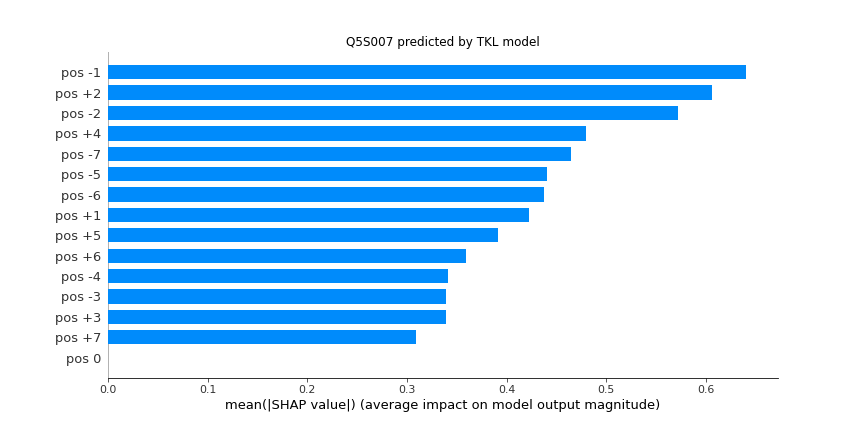 | 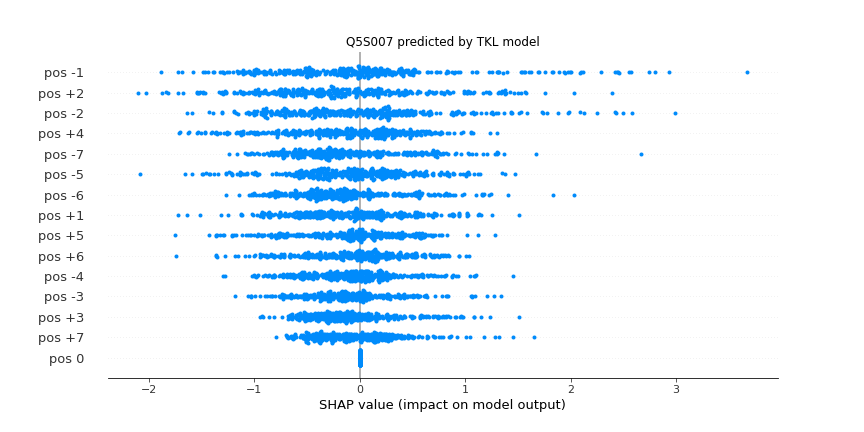 |
| AGC_Akt (STY) | 526 | 0.945 | 0.943 | 0.944 | 0.945 | 0.965 | 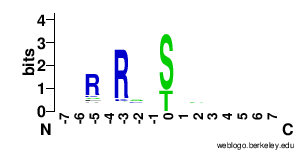 | 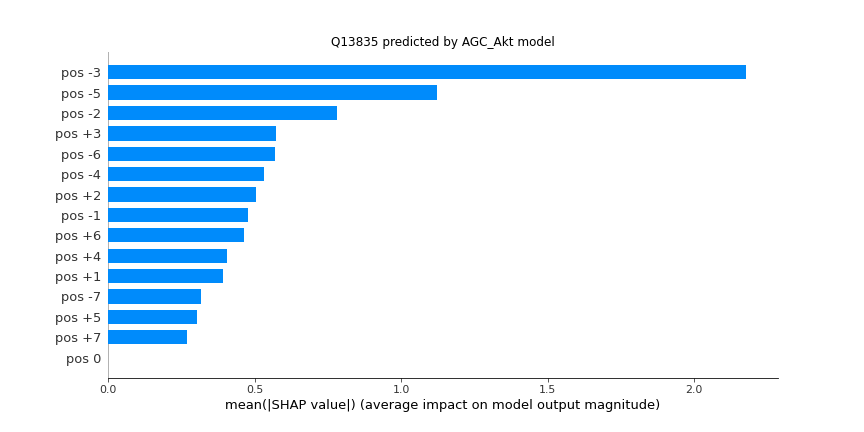 | 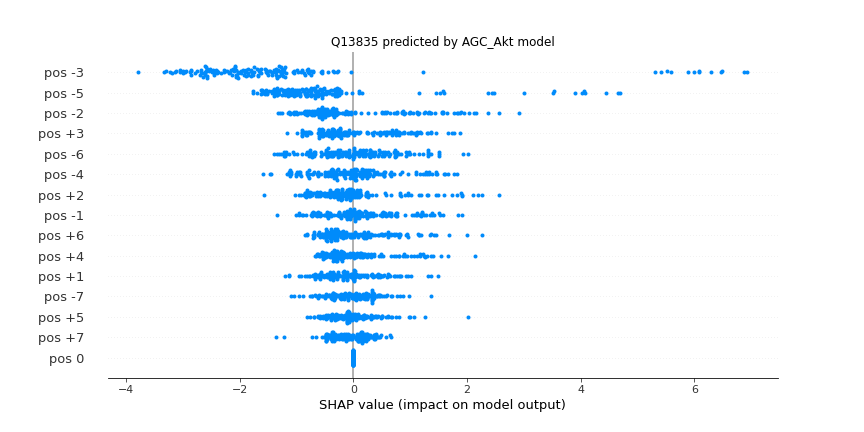 |
| AGC_DMPK (STY) | 154 | 0.899 | 0.889 | 0.895 | 0.899 | 0.87 | 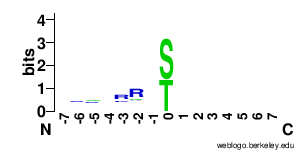 | 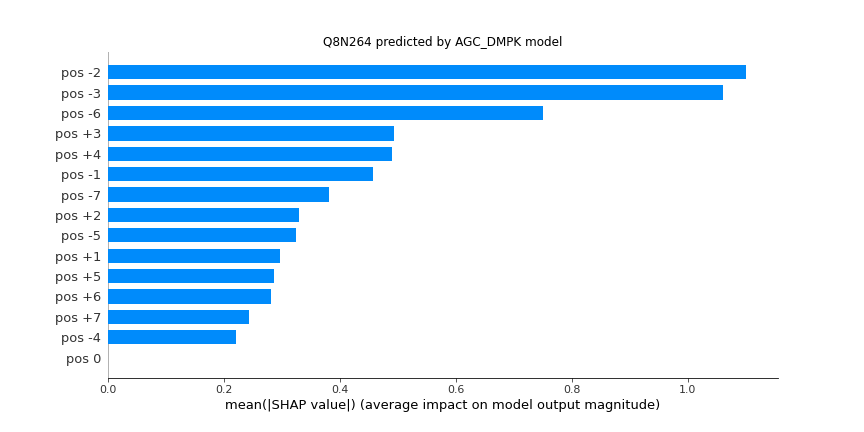 | 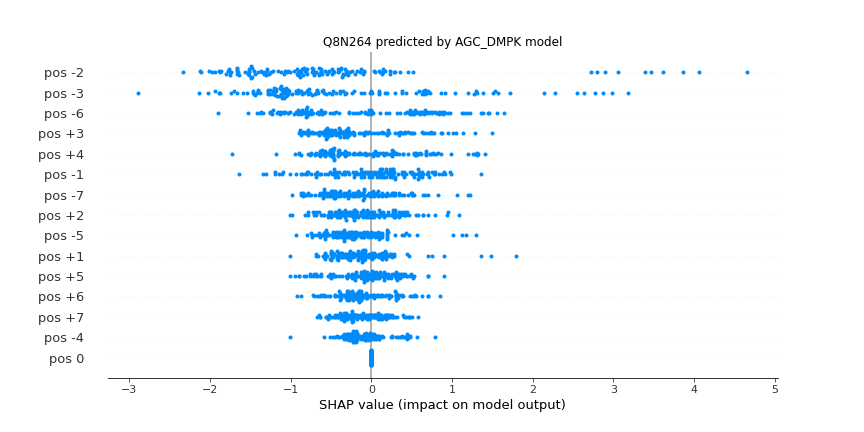 |
| AGC_GRK (STY) | 276 | 0.861 | 0.846 | 0.861 | 0.861 | 0.851 | 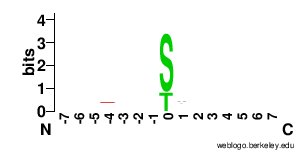 | 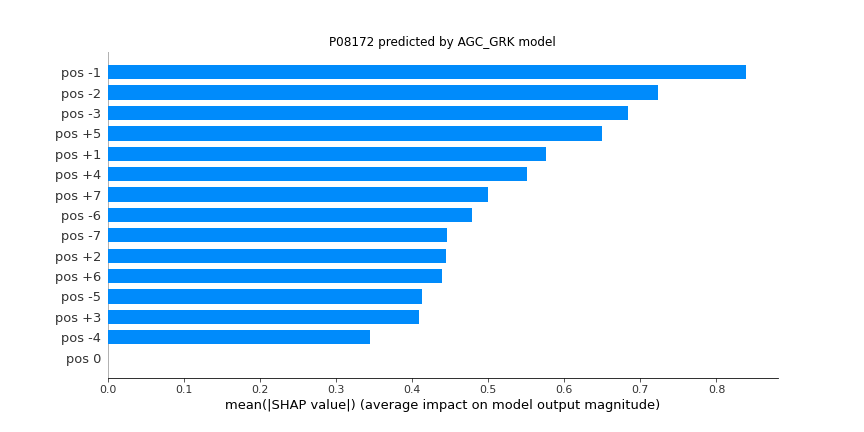 | 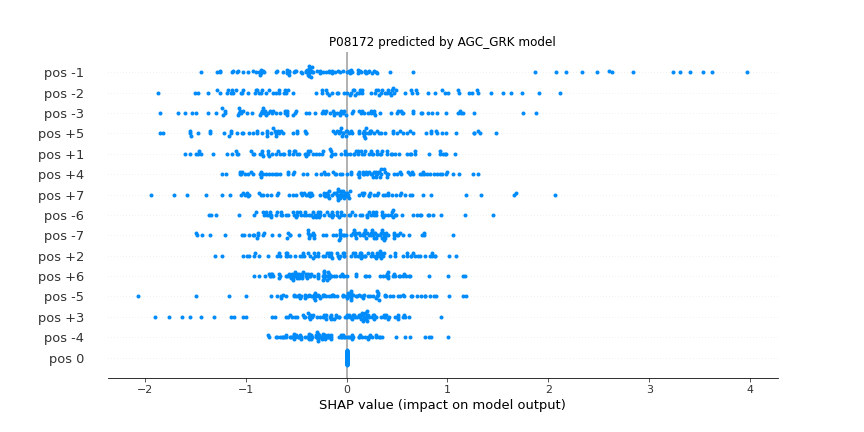 |
| AGC_NDR (STY) | 48 | 0.959 | 0.951 | 0.963 | 0.959 | 0.958 | 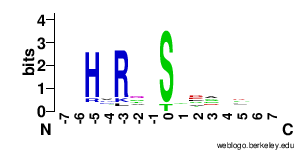 | 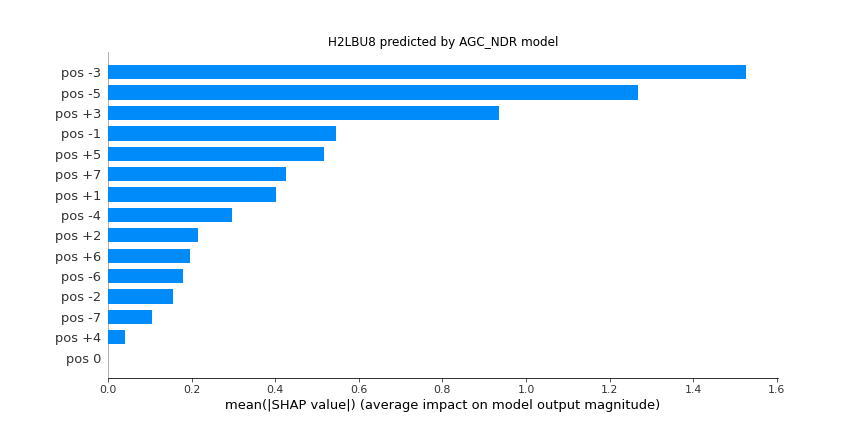 | 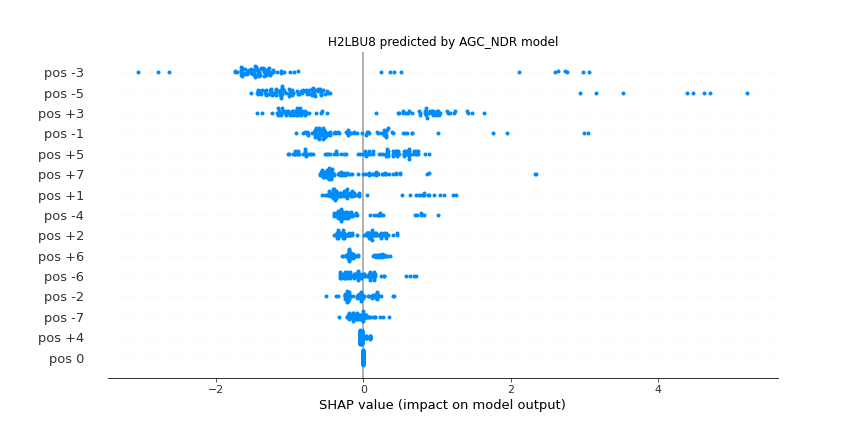 |
| AGC_PDK1 (STY) | 99 | 0.908 | 0.9 | 0.903 | 0.908 | 0.901 | 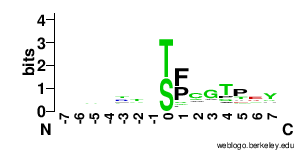 | 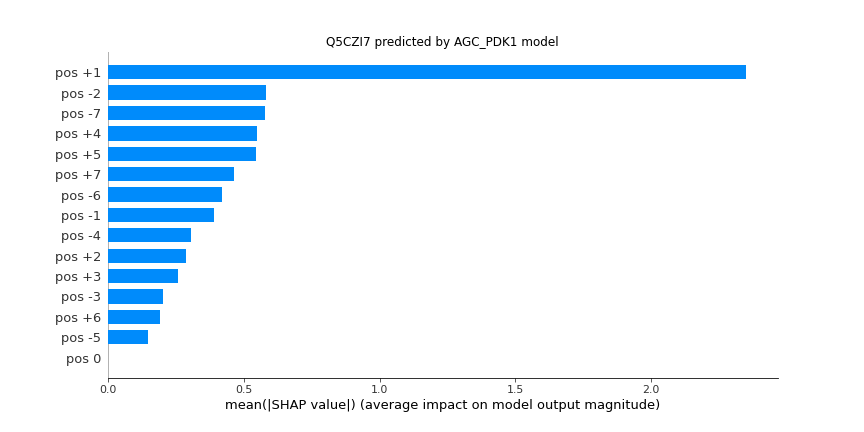 | 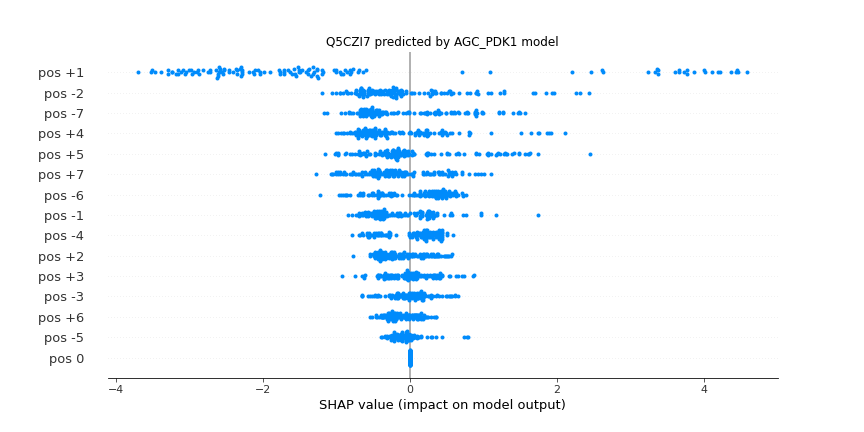 |
| AGC_PKA (STY) | 1769 | 0.909 | 0.908 | 0.911 | 0.909 | 0.961 | 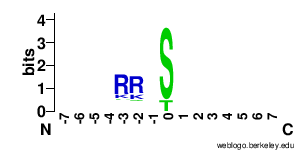 | 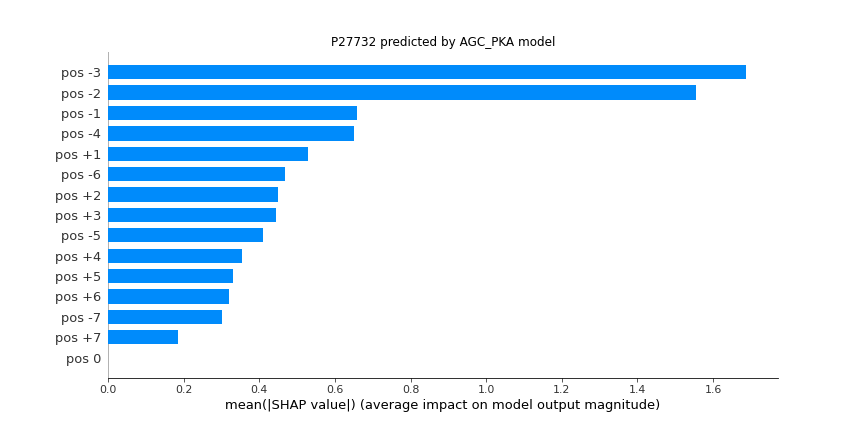 | 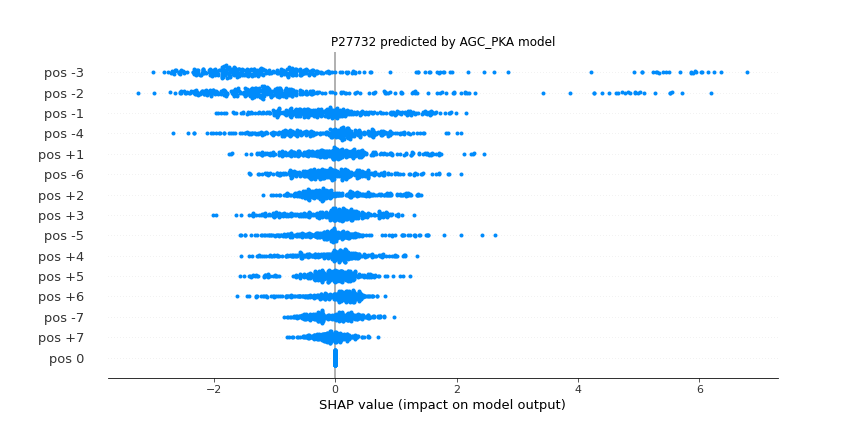 |
| AGC_PKC (STY) | 1641 | 0.852 | 0.851 | 0.853 | 0.852 | 0.92 | 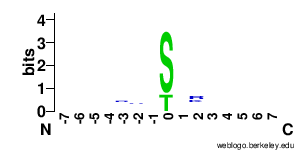 | 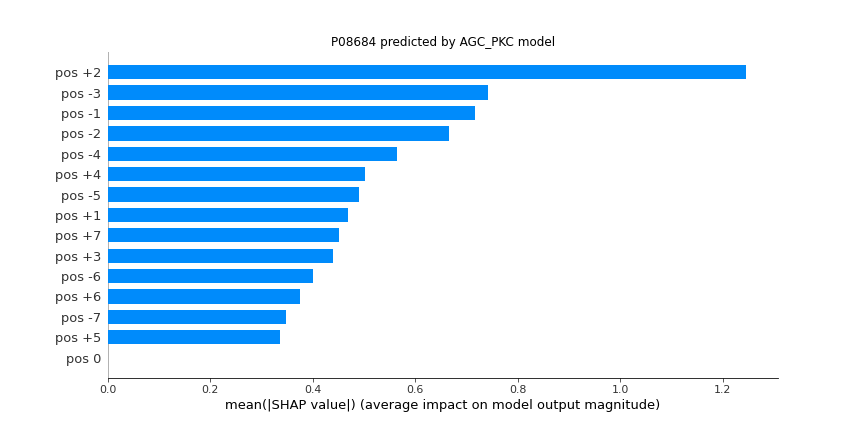 | 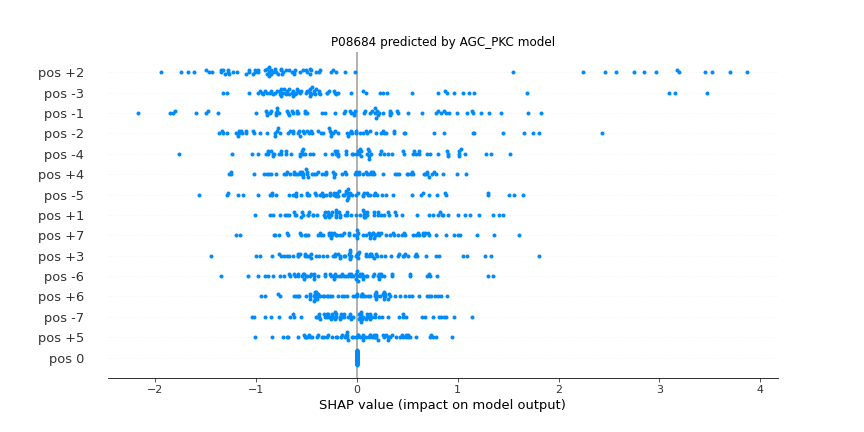 |
| AGC_PKG (STY) | 196 | 0.901 | 0.895 | 0.896 | 0.901 | 0.874 | 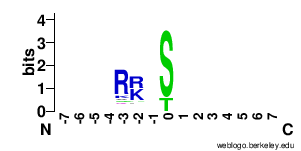 | 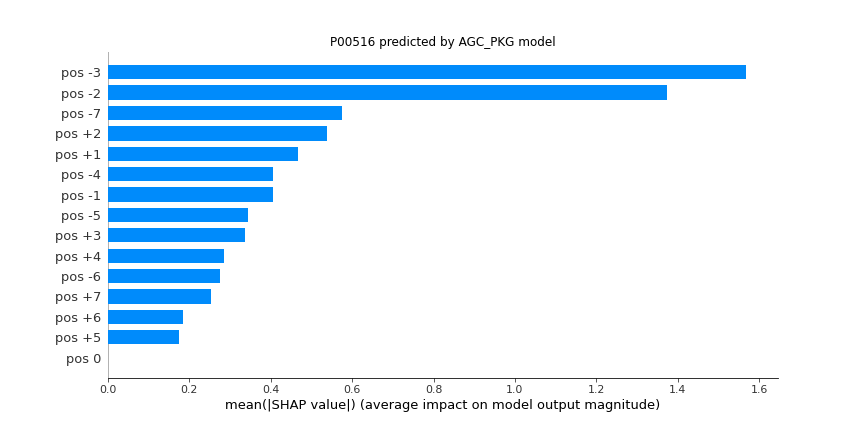 | 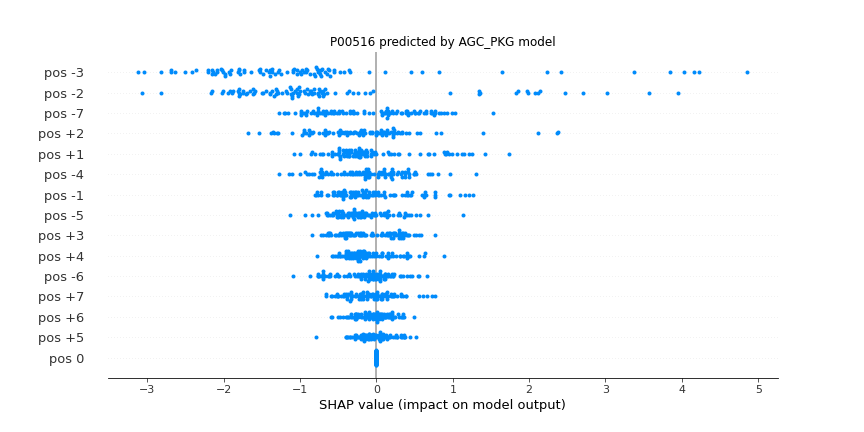 |
| AGC_PKN (STY) | 29 | 0.914 | 0.904 | 0.91 | 0.914 | 0.883 | 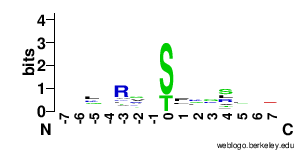 | 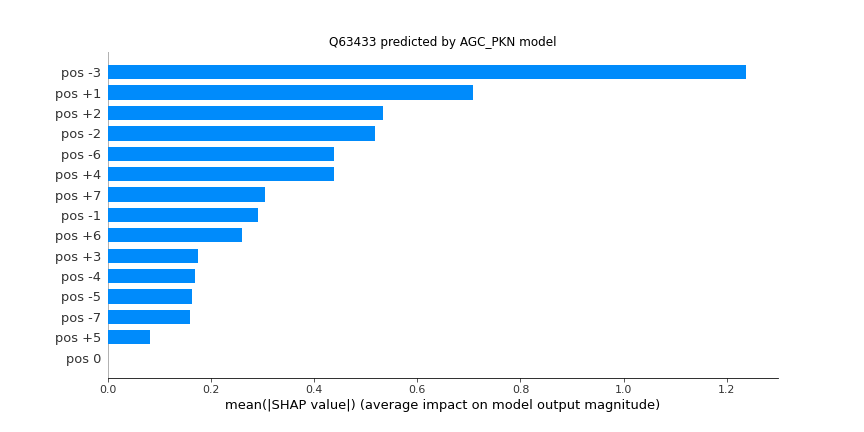 | 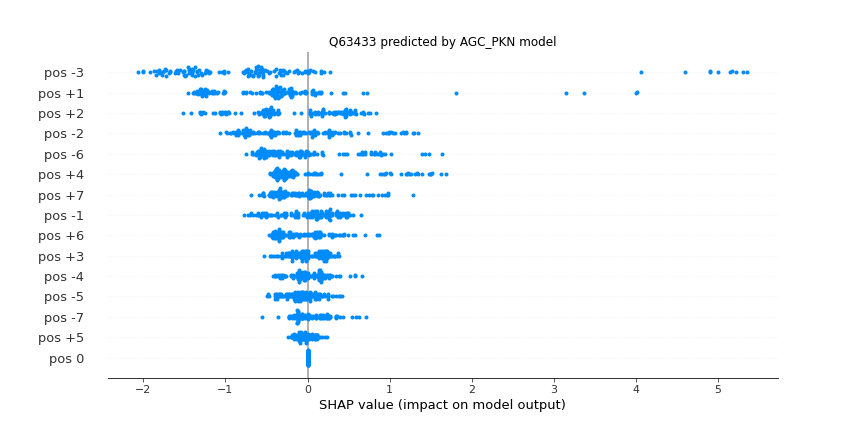 |
| AGC_RSK (STY) | 287 | 0.93 | 0.923 | 0.928 | 0.93 | 0.943 | 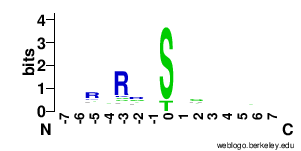 | 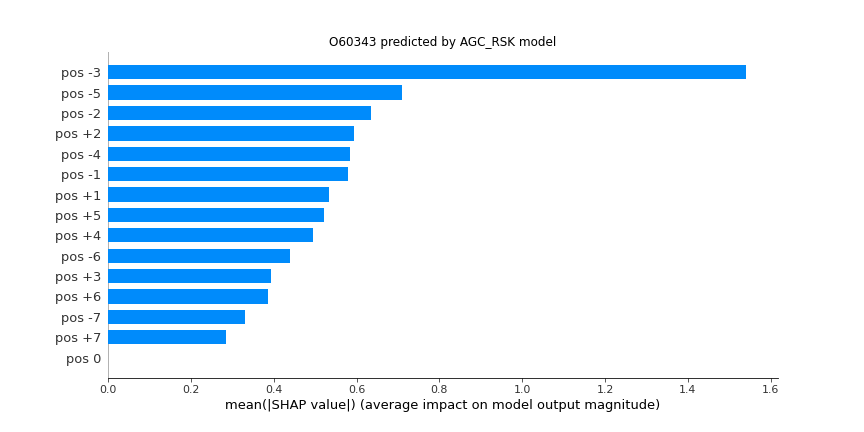 | 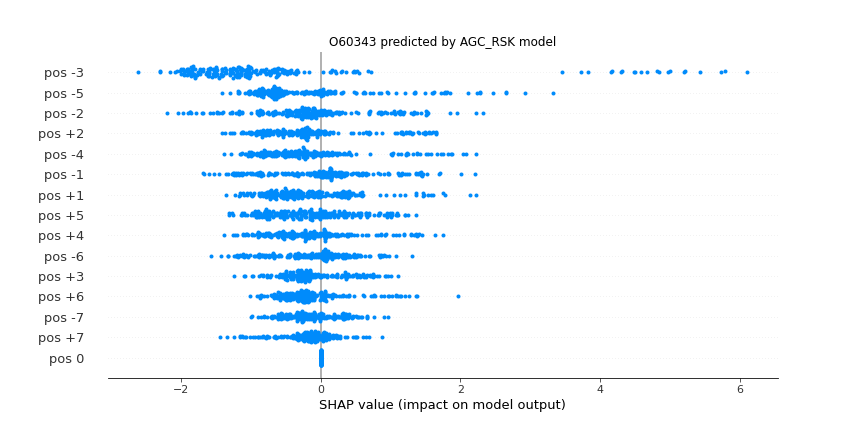 |
| AGC_SGK (STY) | 109 | 0.945 | 0.943 | 0.945 | 0.945 | 0.937 | 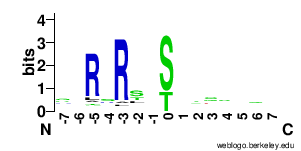 | 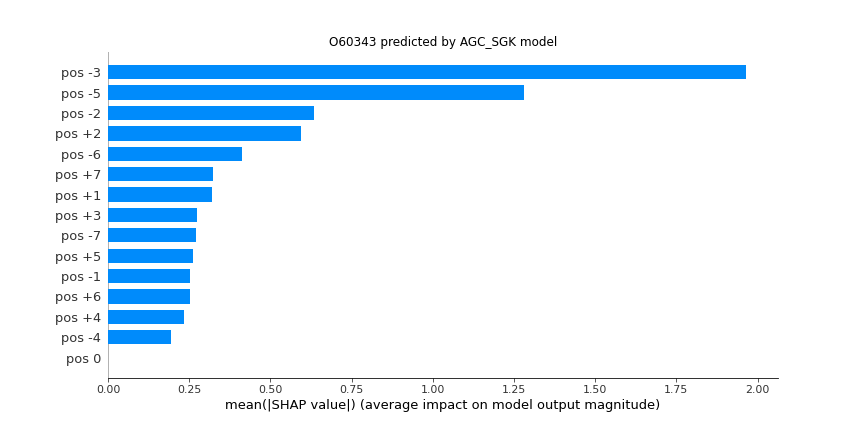 | 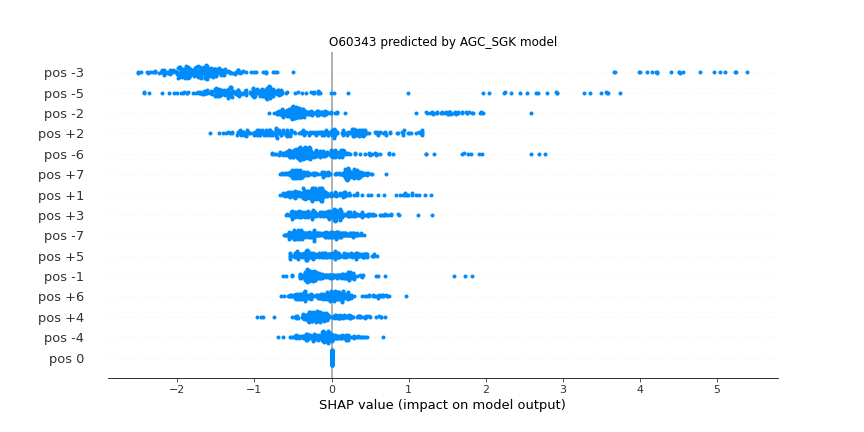 |
| Atypical_Alpha (STY) | 90 | 0.853 | 0.821 | 0.825 | 0.853 | 0.74 | 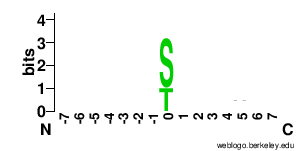 | 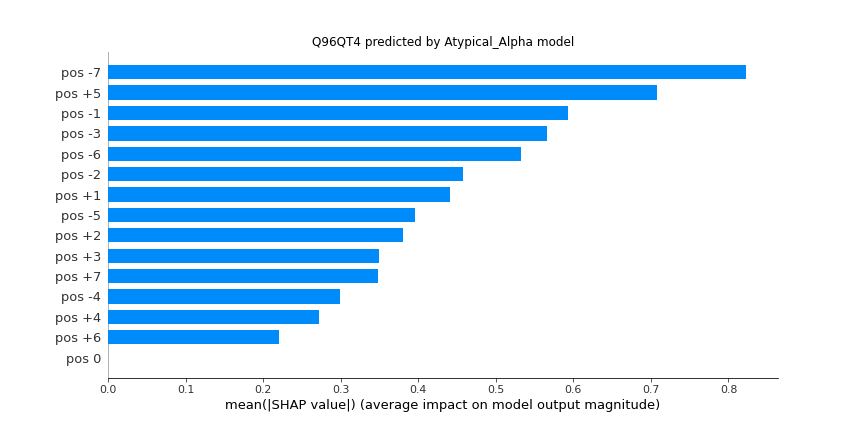 | 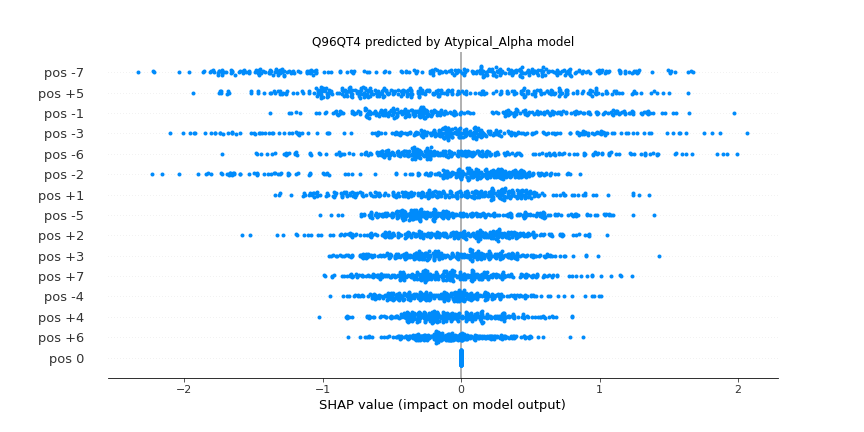 |
| Atypical_PDHK (STY) | 43 | 0.911 | 0.904 | 0.913 | 0.911 | 0.887 | 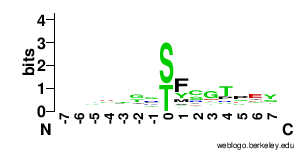 | 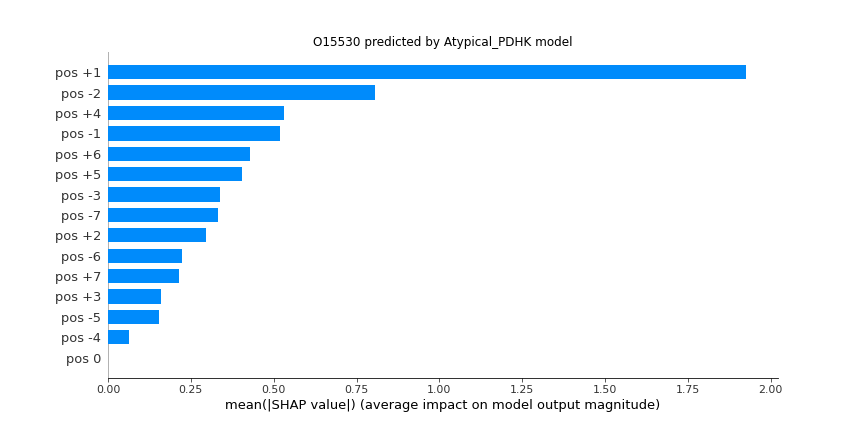 | 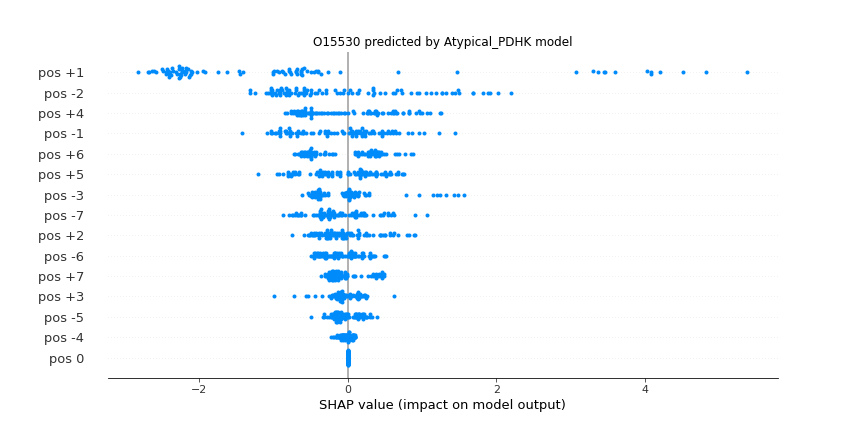 |
| Atypical_PIKK (STY) | 857 | 0.919 | 0.916 | 0.919 | 0.919 | 0.953 | 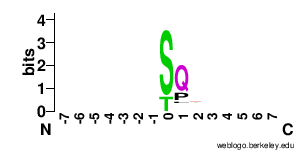 | 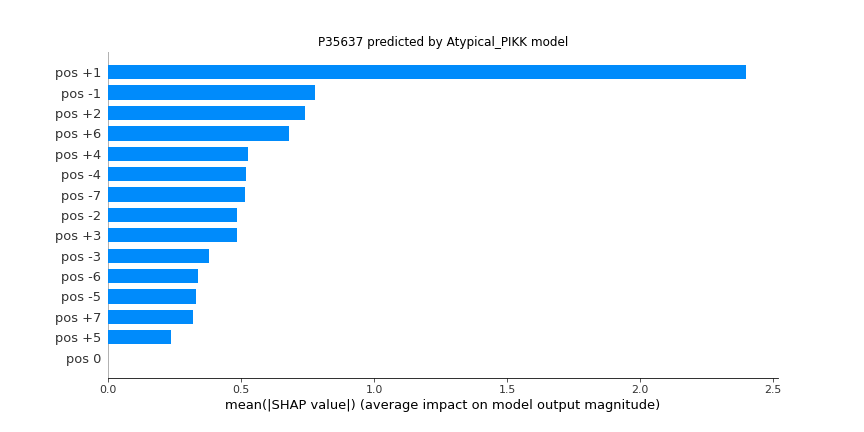 | 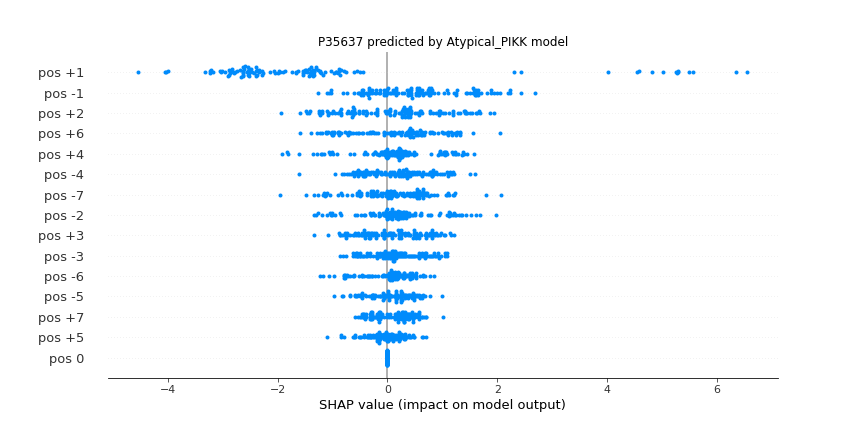 |
| CAMK_CAMK1 (STY) | 87 | 0.916 | 0.911 | 0.915 | 0.916 | 0.878 | 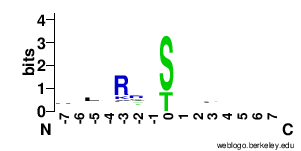 | 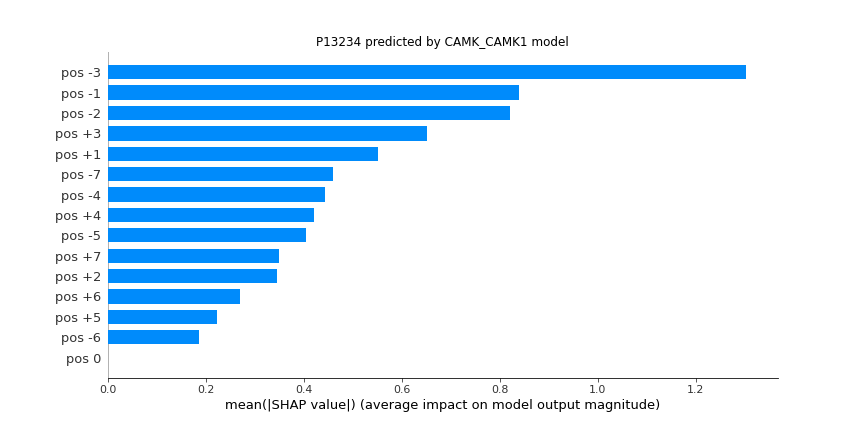 | 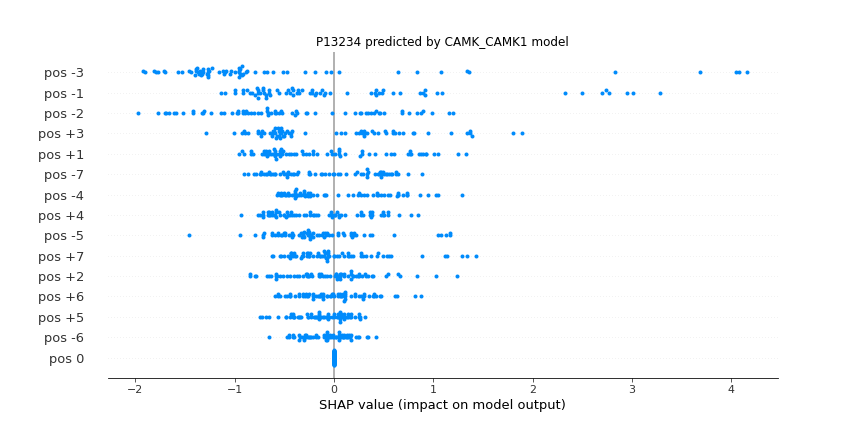 |
| CAMK_CAMK2 (STY) | 474 | 0.872 | 0.862 | 0.864 | 0.872 | 0.884 | 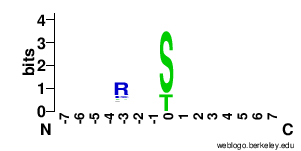 | 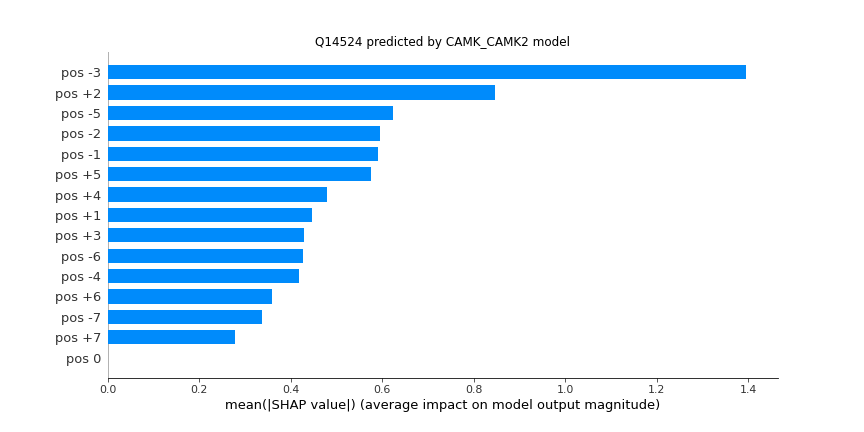 | 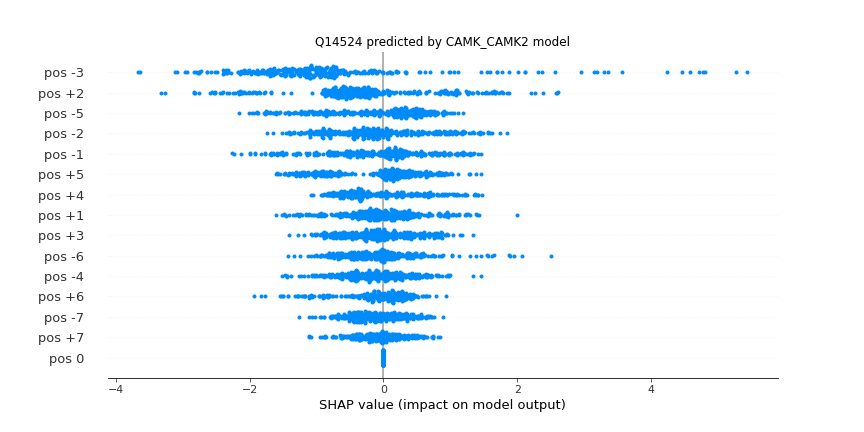 |
| CAMK_CAMKL (STY) | 754 | 0.876 | 0.87 | 0.875 | 0.876 | 0.893 | 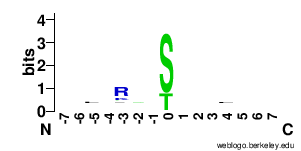 | 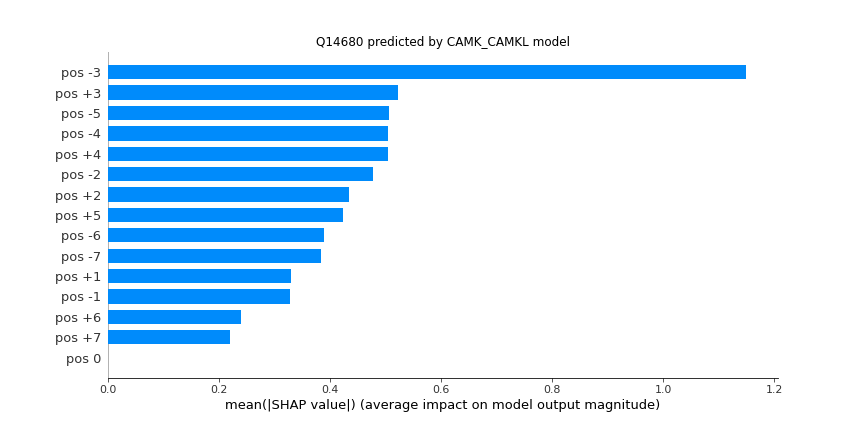 | 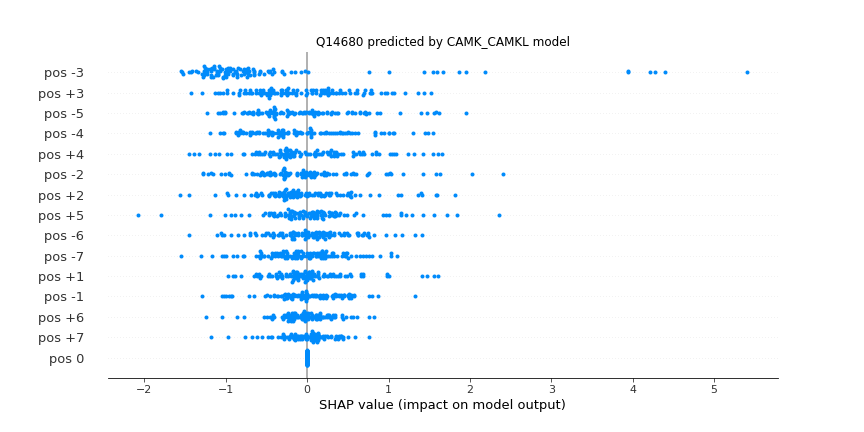 |
| CAMK_DAPK (STY) | 69 | 0.867 | 0.848 | 0.85 | 0.867 | 0.785 | 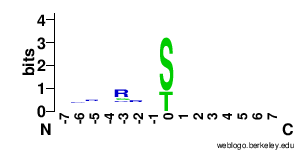 | 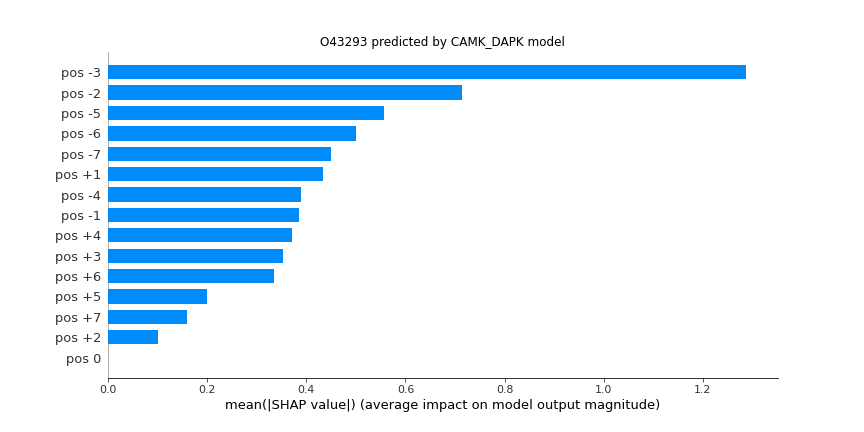 | 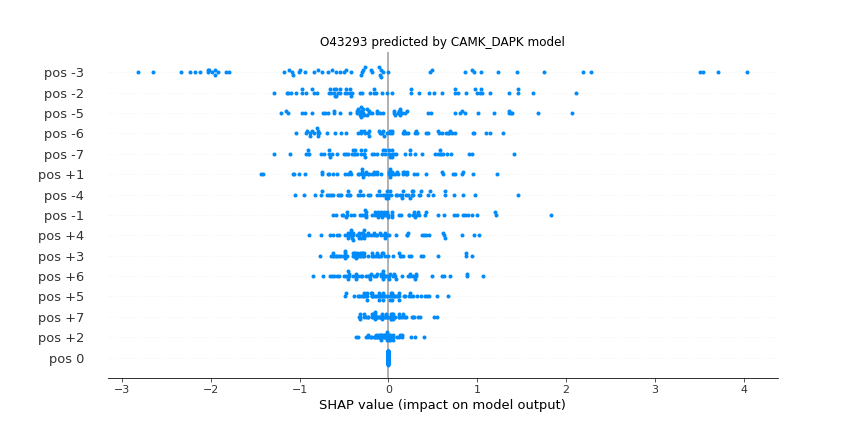 |
| CAMK_MAPKAPK (STY) | 184 | 0.902 | 0.895 | 0.897 | 0.902 | 0.895 | 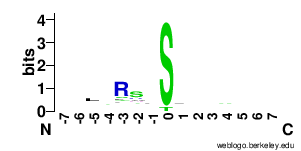 | 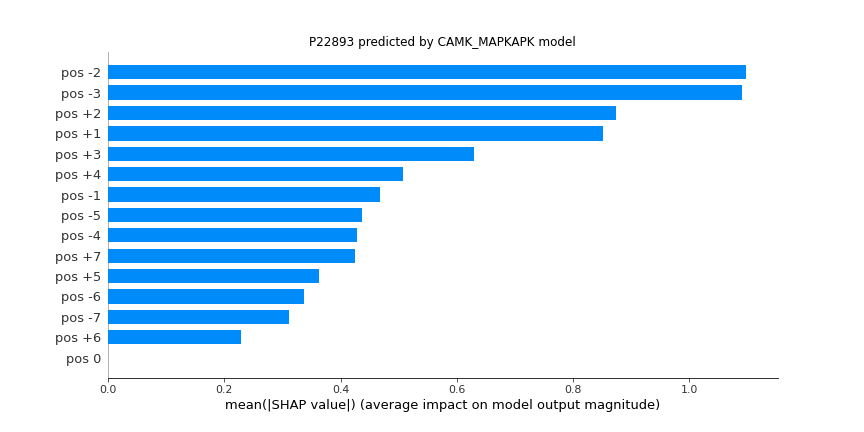 | 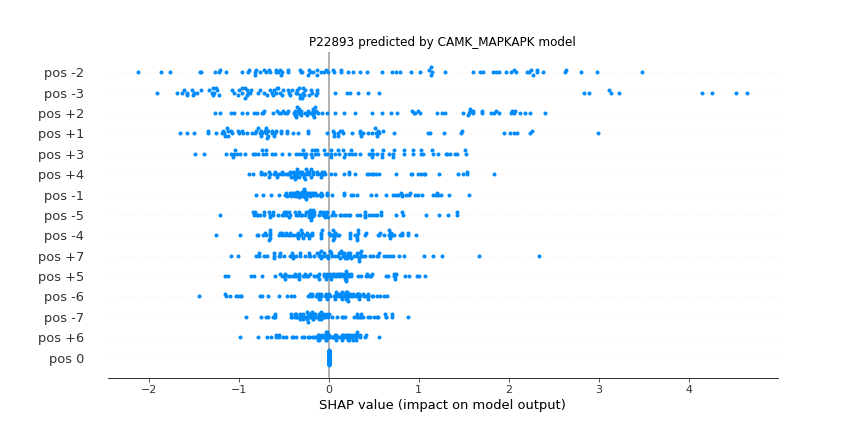 |
| CAMK_MLCK (STY) | 34 | 0.884 | 0.854 | 0.846 | 0.884 | 0.73 | 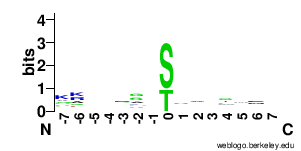 | 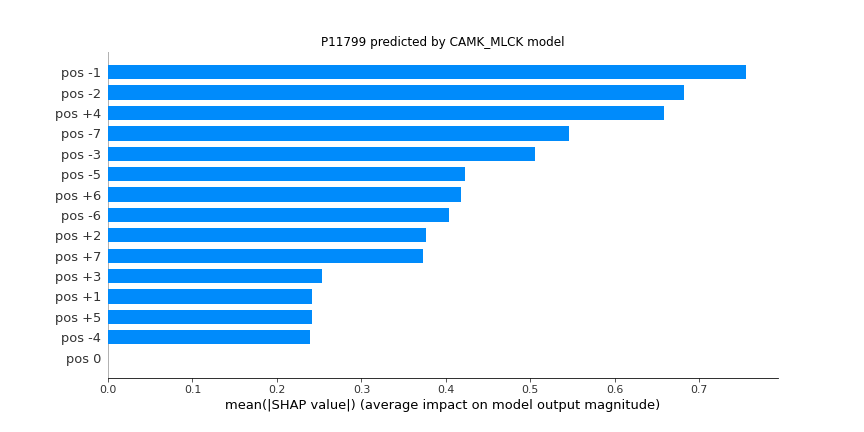 | 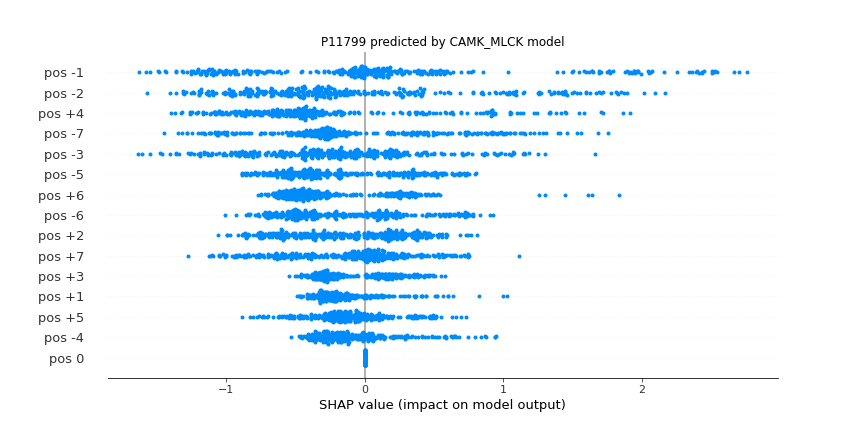 |
| CAMK_PHK (STY) | 30 | 0.85 | 0.825 | 0.823 | 0.85 | 0.647 | 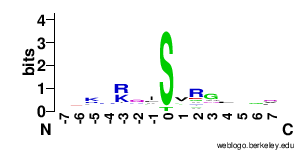 | 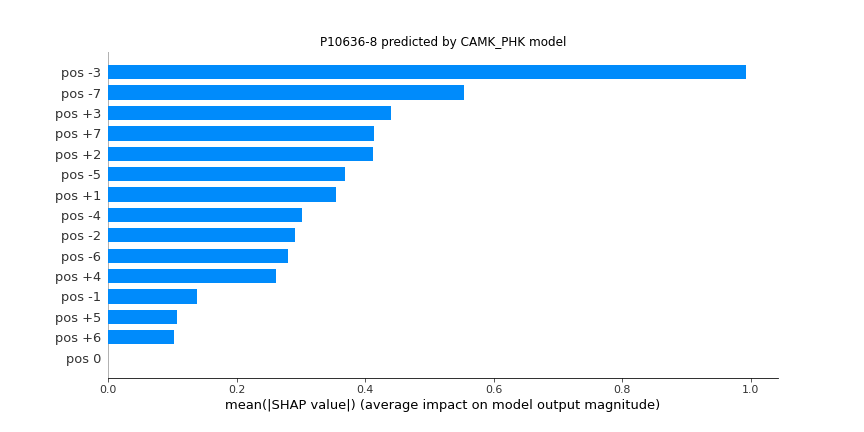 | 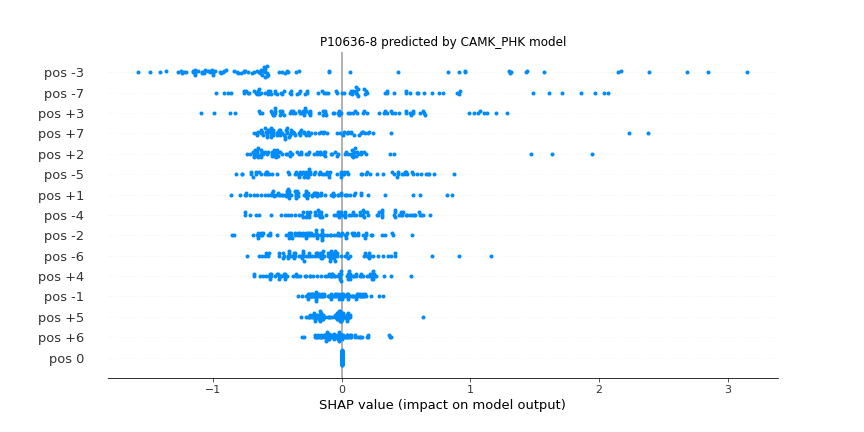 |
| CAMK_PIM (STY) | 84 | 0.893 | 0.879 | 0.889 | 0.893 | 0.84 | 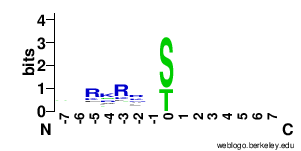 | 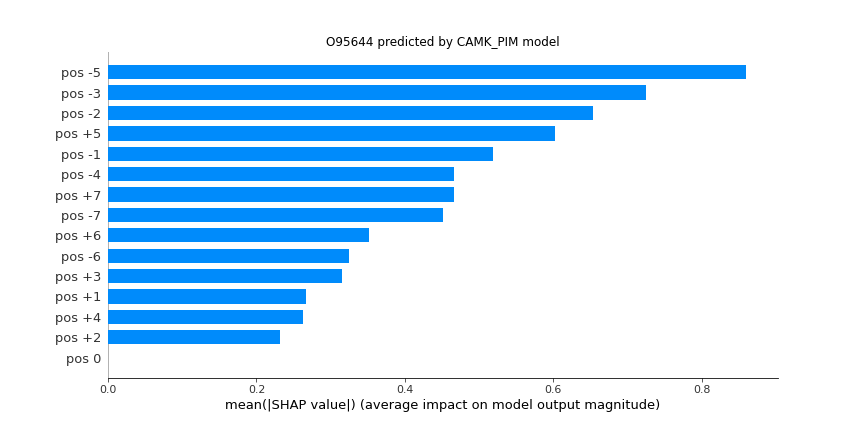 | 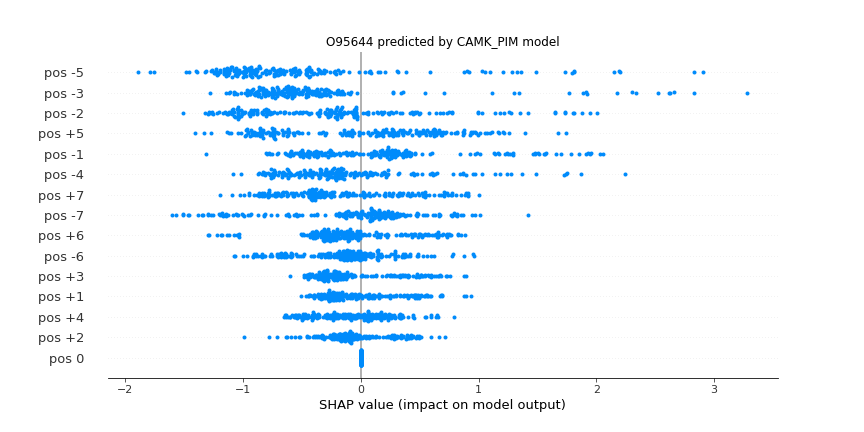 |
| CAMK_PKD (STY) | 111 | 0.933 | 0.926 | 0.932 | 0.933 | 0.908 | 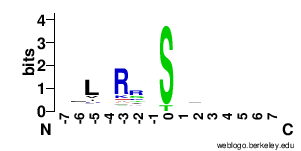 | 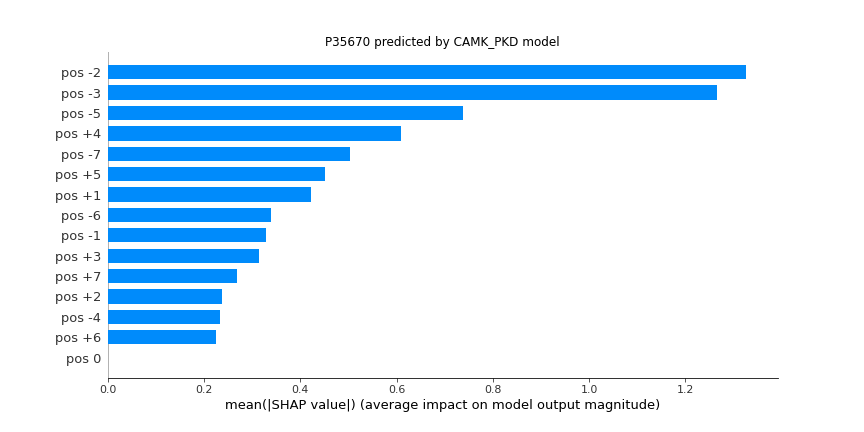 | 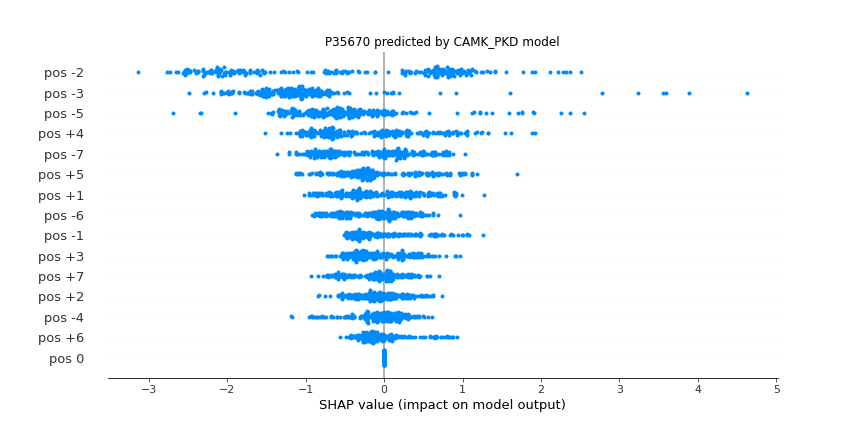 |
| CAMK_RAD53 (STY) | 111 | 0.889 | 0.875 | 0.884 | 0.889 | 0.842 | 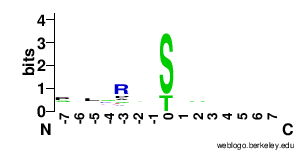 |  |  |
| CK1_CK1 (STY) | 464 | 0.855 | 0.847 | 0.851 | 0.855 | 0.873 |  |  |  |
| CK1_TTBK (STY) | 19 | 0.826 | 0.784 | 0.754 | 0.826 | 0.693 |  |  |  |
| CK1_VRK (STY) | 30 | 0.878 | 0.85 | 0.839 | 0.878 | 0.724 |  |  |  |
| CMGC_CDK (STY) | 2050 | 0.937 | 0.936 | 0.941 | 0.937 | 0.98 |  |  |  |
| CMGC_CK2 (STY) | 1337 | 0.925 | 0.924 | 0.926 | 0.925 | 0.971 |  |  |  |
| CMGC_CLK (STY) | 34 | 0.834 | 0.808 | 0.795 | 0.834 | 0.806 |  |  |  |
| CMGC_DYRK (STY) | 244 | 0.935 | 0.933 | 0.935 | 0.935 | 0.937 |  |  |  |
| CMGC_GSK (STY) | 572 | 0.923 | 0.921 | 0.922 | 0.923 | 0.952 |  |  |  |
| CMGC_MAPK (STY) | 1724 | 0.947 | 0.947 | 0.949 | 0.947 | 0.984 |  |  |  |
| CMGC_RCK (STY) | 21 | 0.856 | 0.818 | 0.793 | 0.856 | 0.831 |  |  |  |
| CMGC_SRPK (STY) | 34 | 0.951 | 0.947 | 0.951 | 0.951 | 0.944 |  |  |  |
| Other_Aur (STY) | 426 | 0.911 | 0.906 | 0.911 | 0.911 | 0.913 |  |  |  |
| Other_BUB (STY) | 28 | 0.75 | 0.713 | 0.69 | 0.75 | 0.694 |  |  |  |
| Other_CAMKK (STY) | 29 | 0.884 | 0.855 | 0.845 | 0.884 | 0.859 |  |  |  |
| Other_CDC7 (STY) | 50 | 0.853 | 0.821 | 0.83 | 0.853 | 0.752 |  |  |  |
| Other_IKK (STY) | 326 | 0.839 | 0.819 | 0.826 | 0.839 | 0.816 |  |  |  |
| Other_KIS (STY) | 15 | 0.911 | 0.898 | 0.896 | 0.911 | 0.895 |  |  |  |
| Other_NAK (STY) | 15 | 0.822 | 0.762 | 0.716 | 0.822 | 0.648 |  |  |  |
| Other_NEK (STY) | 103 | 0.861 | 0.833 | 0.837 | 0.861 | 0.799 |  |  |  |
| Other_NKF2 (STY) | 21 | 0.906 | 0.867 | 0.843 | 0.906 | 0.693 |  |  |  |
| Other_PEK (STY) | 37 | 0.833 | 0.794 | 0.773 | 0.833 | 0.717 |  |  |  |
| Other_PLK (STY) | 526 | 0.848 | 0.836 | 0.841 | 0.848 | 0.859 |  |  |  |
| Other_TTK (STY) | 87 | 0.824 | 0.772 | 0.763 | 0.824 | 0.613 |  |  |  |
| Other_ULK (STY) | 94 | 0.846 | 0.819 | 0.83 | 0.846 | 0.682 |  |  |  |
| Other_WNK (STY) | 26 | 0.841 | 0.816 | 0.813 | 0.841 | 0.64 |  |  |  |
| PKL_FJ (STY) | 204 | 0.897 | 0.89 | 0.892 | 0.897 | 0.876 |  |  |  |
| STE_STE-Unique (STY) | 38 | 0.846 | 0.823 | 0.828 | 0.846 | 0.607 |  |  |  |
| STE_STE11 (STY) | 71 | 0.857 | 0.829 | 0.852 | 0.857 | 0.783 |  |  |  |
| STE_STE20 (STY) | 457 | 0.854 | 0.839 | 0.851 | 0.854 | 0.838 |  |  |  |
| STE_STE7 (STY) | 66 | 0.895 | 0.869 | 0.855 | 0.895 | 0.836 |  |  |  |
| TKL_IRAK (STY) | 47 | 0.808 | 0.773 | 0.775 | 0.808 | 0.668 |  |  |  |
| TKL_LRRK (STY) | 108 | 0.858 | 0.817 | 0.808 | 0.858 | 0.723 |  |  |  |
| TKL_MLK (STY) | 92 | 0.864 | 0.838 | 0.84 | 0.864 | 0.732 |  |  |  |
| TKL_RAF (STY) | 34 | 0.809 | 0.766 | 0.732 | 0.809 | 0.623 |  |  |  |
| TKL_RIPK (STY) | 34 | 0.853 | 0.825 | 0.833 | 0.853 | 0.705 |  |  |  |
| TKL_STKR (STY) | 41 | 0.865 | 0.84 | 0.842 | 0.865 | 0.828 |  |  |  |
| TK_ALK (STY) | 15 | 0.8 | 0.741 | 0.691 | 0.8 | 0.523 |  |  |  |
| TK_Abl (STY) | 312 | 0.876 | 0.859 | 0.87 | 0.876 | 0.829 |  |  |  |
| TK_Ack (STY) | 22 | 0.941 | 0.932 | 0.95 | 0.941 | 0.959 |  |  |  |
| TK_Axl (STY) | 20 | 0.883 | 0.846 | 0.823 | 0.883 | 0.71 |  |  |  |
| TK_Csk (STY) | 91 | 0.952 | 0.948 | 0.954 | 0.952 | 0.918 |  |  |  |
| TK_DDR (STY) | 33 | 0.965 | 0.958 | 0.955 | 0.965 | 0.918 |  |  |  |
| TK_EGFR (STY) | 179 | 0.866 | 0.843 | 0.847 | 0.866 | 0.829 |  |  |  |
| TK_Eph (STY) | 56 | 0.89 | 0.863 | 0.864 | 0.89 | 0.722 |  |  |  |
| TK_FAK (STY) | 156 | 0.919 | 0.908 | 0.917 | 0.919 | 0.895 |  |  |  |
| TK_FGFR (STY) | 81 | 0.873 | 0.843 | 0.836 | 0.873 | 0.743 |  |  |  |
| TK_Fer (STY) | 42 | 0.878 | 0.859 | 0.847 | 0.878 | 0.807 |  |  |  |
| TK_InsR (STY) | 142 | 0.901 | 0.886 | 0.899 | 0.901 | 0.867 |  |  |  |
| TK_Jak (STY) | 174 | 0.898 | 0.881 | 0.893 | 0.898 | 0.784 |  |  |  |
| TK_Met (STY) | 47 | 0.89 | 0.865 | 0.864 | 0.89 | 0.798 |  |  |  |
| TK_PDGFR (STY) | 111 | 0.898 | 0.883 | 0.896 | 0.898 | 0.849 |  |  |  |
| TK_Ret (STY) | 36 | 0.867 | 0.843 | 0.844 | 0.867 | 0.775 |  |  |  |
| TK_Src (STY) | 1321 | 0.795 | 0.793 | 0.795 | 0.795 | 0.86 |  |  |  |
| TK_Syk (STY) | 159 | 0.902 | 0.89 | 0.898 | 0.902 | 0.877 |  |  |  |
| TK_Tec (STY) | 84 | 0.863 | 0.829 | 0.856 | 0.863 | 0.729 |  |  |  |
| TK_Trk (STY) | 32 | 0.87 | 0.841 | 0.833 | 0.87 | 0.83 |  |  |  |
| TK_VEGFR (STY) | 33 | 0.879 | 0.854 | 0.858 | 0.879 | 0.693 |  |  |  |
| AGC_Akt (ST) | 526 | 0.947 | 0.946 | 0.947 | 0.947 | 0.971 |  |  |  |
| AGC_DMPK (ST) | 154 | 0.905 | 0.893 | 0.901 | 0.905 | 0.878 |  |  |  |
| AGC_GRK (ST) | 276 | 0.85 | 0.837 | 0.849 | 0.85 | 0.859 |  |  |  |
| AGC_NDR (ST) | 48 | 0.952 | 0.945 | 0.957 | 0.952 | 0.939 |  |  |  |
| AGC_PDK1 (ST) | 99 | 0.934 | 0.928 | 0.936 | 0.934 | 0.931 |  |  |  |
| AGC_PKA (ST) | 1767 | 0.921 | 0.921 | 0.923 | 0.921 | 0.969 |  |  |  |
| AGC_PKC (ST) | 1635 | 0.862 | 0.862 | 0.865 | 0.862 | 0.935 |  |  |  |
| AGC_PKG (ST) | 196 | 0.929 | 0.924 | 0.928 | 0.929 | 0.895 |  |  |  |
| AGC_PKN (ST) | 29 | 0.885 | 0.87 | 0.863 | 0.885 | 0.843 |  |  |  |
| AGC_RSK (ST) | 286 | 0.928 | 0.922 | 0.925 | 0.928 | 0.936 |  |  |  |
| AGC_SGK (ST) | 109 | 0.951 | 0.948 | 0.952 | 0.951 | 0.954 |  |  |  |
| Atypical_Alpha (ST) | 90 | 0.821 | 0.795 | 0.801 | 0.821 | 0.702 |  |  |  |
| Atypical_PDHK (ST) | 43 | 0.915 | 0.908 | 0.915 | 0.915 | 0.88 |  |  |  |
| Atypical_PIKK (ST) | 855 | 0.927 | 0.926 | 0.929 | 0.927 | 0.962 |  |  |  |
| CAMK_CAMK1 (ST) | 87 | 0.923 | 0.917 | 0.925 | 0.923 | 0.882 |  |  |  |
| CAMK_CAMK2 (ST) | 474 | 0.851 | 0.844 | 0.846 | 0.851 | 0.882 |  |  |  |
| CAMK_CAMKL (ST) | 747 | 0.882 | 0.878 | 0.884 | 0.882 | 0.908 |  |  |  |
| CAMK_DAPK (ST) | 69 | 0.87 | 0.858 | 0.861 | 0.87 | 0.798 |  |  |  |
| CAMK_MAPKAPK (ST) | 184 | 0.91 | 0.902 | 0.904 | 0.91 | 0.916 |  |  |  |
| CAMK_MLCK (ST) | 34 | 0.864 | 0.831 | 0.813 | 0.864 | 0.756 |  |  |  |
| CAMK_PHK (ST) | 30 | 0.872 | 0.848 | 0.853 | 0.872 | 0.716 |  |  |  |
| CAMK_PIM (ST) | 84 | 0.887 | 0.876 | 0.884 | 0.887 | 0.833 |  |  |  |
| CAMK_PKD (ST) | 111 | 0.943 | 0.938 | 0.942 | 0.943 | 0.932 |  |  |  |
| CAMK_RAD53 (ST) | 111 | 0.889 | 0.873 | 0.881 | 0.889 | 0.846 |  |  |  |
| CK1_CK1 (ST) | 463 | 0.855 | 0.851 | 0.854 | 0.855 | 0.892 |  |  |  |
| CK1_TTBK (ST) | 18 | 0.835 | 0.792 | 0.762 | 0.835 | 0.689 |  |  |  |
| CK1_VRK (ST) | 30 | 0.889 | 0.864 | 0.87 | 0.889 | 0.691 |  |  |  |
| CMGC_CDK (ST) | 2046 | 0.946 | 0.946 | 0.95 | 0.946 | 0.985 |  |  |  |
| CMGC_CK2 (ST) | 1332 | 0.924 | 0.924 | 0.925 | 0.924 | 0.974 |  |  |  |
| CMGC_CLK (ST) | 32 | 0.863 | 0.829 | 0.822 | 0.863 | 0.896 |  |  |  |
| CMGC_DYRK (ST) | 234 | 0.943 | 0.941 | 0.943 | 0.943 | 0.953 |  |  |  |
| CMGC_GSK (ST) | 571 | 0.92 | 0.919 | 0.92 | 0.92 | 0.96 |  |  |  |
| CMGC_MAPK (ST) | 1715 | 0.953 | 0.953 | 0.956 | 0.953 | 0.988 |  |  |  |
| CMGC_RCK (ST) | 18 | 0.863 | 0.833 | 0.813 | 0.863 | 0.833 |  |  |  |
| CMGC_SRPK (ST) | 34 | 0.911 | 0.906 | 0.91 | 0.911 | 0.896 |  |  |  |
| Other_Aur (ST) | 424 | 0.907 | 0.902 | 0.91 | 0.907 | 0.925 |  |  |  |
| Other_BUB (ST) | 28 | 0.743 | 0.703 | 0.69 | 0.743 | 0.6 |  |  |  |
| Other_CAMKK (ST) | 29 | 0.884 | 0.859 | 0.845 | 0.884 | 0.835 |  |  |  |
| Other_CDC7 (ST) | 50 | 0.857 | 0.828 | 0.823 | 0.857 | 0.779 |  |  |  |
| Other_IKK (ST) | 326 | 0.841 | 0.829 | 0.834 | 0.841 | 0.851 |  |  |  |
| Other_KIS (ST) | 15 | 0.878 | 0.863 | 0.873 | 0.878 | 0.889 |  |  |  |
| Other_NEK (ST) | 100 | 0.845 | 0.821 | 0.823 | 0.845 | 0.777 |  |  |  |
| Other_NKF2 (ST) | 21 | 0.874 | 0.842 | 0.821 | 0.874 | 0.683 |  |  |  |
| Other_PEK (ST) | 30 | 0.839 | 0.789 | 0.76 | 0.839 | 0.613 |  |  |  |
| Other_PLK (ST) | 525 | 0.829 | 0.821 | 0.824 | 0.829 | 0.867 |  |  |  |
| Other_TTK (ST) | 86 | 0.829 | 0.787 | 0.791 | 0.829 | 0.7 |  |  |  |
| Other_ULK (ST) | 94 | 0.826 | 0.791 | 0.778 | 0.826 | 0.644 |  |  |  |
| Other_WNK (ST) | 26 | 0.855 | 0.826 | 0.818 | 0.855 | 0.691 |  |  |  |
| PKL_FJ (ST) | 204 | 0.893 | 0.886 | 0.888 | 0.893 | 0.884 |  |  |  |
| STE_STE-Unique (ST) | 37 | 0.874 | 0.852 | 0.858 | 0.874 | 0.715 |  |  |  |
| STE_STE11 (ST) | 69 | 0.848 | 0.819 | 0.83 | 0.848 | 0.785 |  |  |  |
| STE_STE20 (ST) | 457 | 0.833 | 0.822 | 0.828 | 0.833 | 0.829 |  |  |  |
| STE_STE7 (ST) | 46 | 0.9 | 0.876 | 0.887 | 0.9 | 0.834 |  |  |  |
| TKL_IRAK (ST) | 47 | 0.796 | 0.748 | 0.735 | 0.796 | 0.68 |  |  |  |
| TKL_LRRK (ST) | 105 | 0.845 | 0.811 | 0.814 | 0.845 | 0.737 |  |  |  |
| TKL_MLK (ST) | 92 | 0.862 | 0.838 | 0.852 | 0.862 | 0.727 |  |  |  |
| TKL_RAF (ST) | 34 | 0.839 | 0.797 | 0.782 | 0.839 | 0.612 |  |  |  |
| TKL_RIPK (ST) | 33 | 0.828 | 0.794 | 0.777 | 0.828 | 0.739 |  |  |  |
| TKL_STKR (ST) | 38 | 0.873 | 0.843 | 0.838 | 0.873 | 0.821 |  |  |  |
| TK_DDR (ST) | 30 | 0.978 | 0.976 | 0.976 | 0.978 | 0.969 |  |  |  |
| TK_FAK (ST) | 86 | 0.957 | 0.955 | 0.958 | 0.957 | 0.94 |  |  |  |
| STE_STE7 (Y) | 20 | 0.874 | 0.855 | 0.857 | 0.874 | 0.84 |  |  |  |
| TK_Abl (Y) | 311 | 0.767 | 0.764 | 0.768 | 0.767 | 0.827 |  |  |  |
| TK_Ack (Y) | 21 | 0.9 | 0.886 | 0.891 | 0.9 | 0.879 |  |  |  |
| TK_Axl (Y) | 18 | 0.861 | 0.837 | 0.824 | 0.861 | 0.706 |  |  |  |
| TK_Csk (Y) | 91 | 0.859 | 0.855 | 0.87 | 0.859 | 0.927 |  |  |  |
| TK_EGFR (Y) | 178 | 0.807 | 0.796 | 0.804 | 0.807 | 0.836 |  |  |  |
| TK_Eph (Y) | 56 | 0.784 | 0.761 | 0.758 | 0.784 | 0.735 |  |  |  |
| TK_FAK (Y) | 70 | 0.844 | 0.827 | 0.831 | 0.844 | 0.787 |  |  |  |
| TK_FGFR (Y) | 81 | 0.801 | 0.771 | 0.762 | 0.801 | 0.752 |  |  |  |
| TK_Fer (Y) | 42 | 0.861 | 0.844 | 0.853 | 0.861 | 0.838 |  |  |  |
| TK_InsR (Y) | 139 | 0.804 | 0.789 | 0.808 | 0.804 | 0.838 |  |  |  |
| TK_Jak (Y) | 172 | 0.788 | 0.778 | 0.792 | 0.788 | 0.802 |  |  |  |
| TK_Met (Y) | 47 | 0.829 | 0.814 | 0.83 | 0.829 | 0.78 |  |  |  |
| TK_PDGFR (Y) | 111 | 0.78 | 0.772 | 0.781 | 0.78 | 0.822 |  |  |  |
| TK_Ret (Y) | 32 | 0.843 | 0.798 | 0.779 | 0.843 | 0.772 |  |  |  |
| TK_Src (Y) | 1310 | 0.853 | 0.844 | 0.848 | 0.853 | 0.903 |  |  |  |
| TK_Syk (Y) | 154 | 0.838 | 0.833 | 0.846 | 0.838 | 0.877 |  |  |  |
| TK_Tec (Y) | 80 | 0.801 | 0.776 | 0.791 | 0.801 | 0.691 |  |  |  |
| TK_Trk (Y) | 32 | 0.876 | 0.851 | 0.853 | 0.876 | 0.814 |  |  |  |
| TK_VEGFR (Y) | 33 | 0.824 | 0.794 | 0.804 | 0.824 | 0.756 |  |  |  |
| A6QLB8 (STY) | 16 | 0.78 | 0.752 | 0.781 | 0.78 | 0.833 |  |  |  |
| A9UF07 (STY) | 18 | 0.797 | 0.751 | 0.715 | 0.797 | 0.578 |  |  |  |
| G3N1T2 (STY) | 24 | 0.855 | 0.82 | 0.809 | 0.855 | 0.828 |  |  |  |
| O00141 (STY) | 76 | 0.952 | 0.951 | 0.955 | 0.952 | 0.955 |  |  |  |
| O00311 (STY) | 48 | 0.837 | 0.809 | 0.797 | 0.837 | 0.71 |  |  |  |
| O00418 (STY) | 16 | 0.806 | 0.746 | 0.702 | 0.806 | 0.699 |  |  |  |
| O00444 (STY) | 15 | 0.767 | 0.723 | 0.686 | 0.767 | 0.614 |  |  |  |
| O14757 (STY) | 205 | 0.885 | 0.876 | 0.879 | 0.885 | 0.86 |  |  |  |
| O14920 (STY) | 96 | 0.832 | 0.792 | 0.792 | 0.832 | 0.74 |  |  |  |
| O14965 (STY) | 145 | 0.891 | 0.88 | 0.884 | 0.891 | 0.835 |  |  |  |
| O15111 (STY) | 56 | 0.824 | 0.782 | 0.77 | 0.824 | 0.683 |  |  |  |
| O15264 (STY) | 38 | 0.922 | 0.915 | 0.916 | 0.922 | 0.893 |  |  |  |
| O15530 (STY) | 76 | 0.917 | 0.91 | 0.913 | 0.917 | 0.918 |  |  |  |
| O43293 (STY) | 24 | 0.889 | 0.868 | 0.869 | 0.889 | 0.742 |  |  |  |
| O43318 (STY) | 43 | 0.861 | 0.832 | 0.835 | 0.861 | 0.747 |  |  |  |
| O43683 (STY) | 27 | 0.788 | 0.756 | 0.757 | 0.788 | 0.647 |  |  |  |
| O55099 (STY) | 18 | 0.87 | 0.861 | 0.876 | 0.87 | 0.811 |  |  |  |
| O55173 (STY) | 31 | 0.935 | 0.919 | 0.927 | 0.935 | 0.911 |  |  |  |
| O60674 (STY) | 61 | 0.82 | 0.769 | 0.743 | 0.82 | 0.65 |  |  |  |
| O70126 (STY) | 34 | 0.883 | 0.87 | 0.869 | 0.883 | 0.862 |  |  |  |
| O70405 (STY) | 17 | 0.795 | 0.75 | 0.717 | 0.795 | 0.495 |  |  |  |
| O75116 (STY) | 48 | 0.861 | 0.83 | 0.846 | 0.861 | 0.758 |  |  |  |
| O75385 (STY) | 80 | 0.812 | 0.778 | 0.762 | 0.812 | 0.739 |  |  |  |
| O75582 (STY) | 33 | 0.899 | 0.887 | 0.889 | 0.899 | 0.857 |  |  |  |
| O88351 (STY) | 39 | 0.811 | 0.775 | 0.76 | 0.811 | 0.638 |  |  |  |
| O88643 (STY) | 22 | 0.848 | 0.813 | 0.793 | 0.848 | 0.797 |  |  |  |
| O95747 (STY) | 15 | 0.933 | 0.917 | 0.908 | 0.933 | 0.879 |  |  |  |
| O95835 (STY) | 17 | 0.952 | 0.94 | 0.939 | 0.952 | 0.904 |  |  |  |
| O96013 (STY) | 21 | 0.849 | 0.818 | 0.806 | 0.849 | 0.806 |  |  |  |
| O96017 (STY) | 74 | 0.878 | 0.858 | 0.874 | 0.878 | 0.85 |  |  |  |
| P00516 (STY) | 54 | 0.919 | 0.907 | 0.929 | 0.919 | 0.873 |  |  |  |
| P00517 (STY) | 216 | 0.91 | 0.907 | 0.907 | 0.91 | 0.929 |  |  |  |
| P00519 (STY) | 226 | 0.86 | 0.841 | 0.846 | 0.86 | 0.782 |  |  |  |
| P00520 (STY) | 82 | 0.843 | 0.817 | 0.828 | 0.843 | 0.775 |  |  |  |
| P00523 (STY) | 47 | 0.848 | 0.825 | 0.814 | 0.848 | 0.785 |  |  |  |
| P00533 (STY) | 117 | 0.833 | 0.794 | 0.776 | 0.833 | 0.726 |  |  |  |
| P00546 (STY) | 93 | 0.871 | 0.854 | 0.862 | 0.871 | 0.779 |  |  |  |
| P04049 (STY) | 20 | 0.875 | 0.852 | 0.843 | 0.875 | 0.77 |  |  |  |
| P04409 (STY) | 59 | 0.878 | 0.864 | 0.883 | 0.878 | 0.892 |  |  |  |
| P04551 (STY) | 38 | 0.965 | 0.965 | 0.968 | 0.965 | 0.993 |  |  |  |
| P04626 (STY) | 17 | 0.853 | 0.798 | 0.763 | 0.853 | 0.708 |  |  |  |
| P04629 (STY) | 15 | 0.811 | 0.76 | 0.724 | 0.811 | 0.564 |  |  |  |
| P05129 (STY) | 56 | 0.828 | 0.797 | 0.774 | 0.828 | 0.773 |  |  |  |
| P05132 (STY) | 194 | 0.93 | 0.928 | 0.929 | 0.93 | 0.94 |  |  |  |
| P05480 (STY) | 159 | 0.868 | 0.85 | 0.855 | 0.868 | 0.855 |  |  |  |
| P05696 (STY) | 187 | 0.881 | 0.87 | 0.87 | 0.881 | 0.858 |  |  |  |
| P05771 (STY) | 101 | 0.866 | 0.843 | 0.852 | 0.866 | 0.764 |  |  |  |
| P06213 (STY) | 73 | 0.838 | 0.802 | 0.797 | 0.838 | 0.739 |  |  |  |
| P06239 (STY) | 134 | 0.866 | 0.842 | 0.857 | 0.866 | 0.811 |  |  |  |
| P06240 (STY) | 34 | 0.873 | 0.857 | 0.879 | 0.873 | 0.809 |  |  |  |
| P06241 (STY) | 207 | 0.86 | 0.839 | 0.843 | 0.86 | 0.808 |  |  |  |
| P06493 (STY) | 760 | 0.959 | 0.959 | 0.959 | 0.959 | 0.971 |  |  |  |
| P07947 (STY) | 25 | 0.813 | 0.764 | 0.73 | 0.813 | 0.611 |  |  |  |
| P07948 (STY) | 143 | 0.859 | 0.833 | 0.839 | 0.859 | 0.759 |  |  |  |
| P07949 (STY) | 31 | 0.844 | 0.801 | 0.772 | 0.844 | 0.762 |  |  |  |
| P08069 (STY) | 29 | 0.799 | 0.767 | 0.745 | 0.799 | 0.655 |  |  |  |
| P08103 (STY) | 15 | 0.811 | 0.754 | 0.71 | 0.811 | 0.804 |  |  |  |
| P08413 (STY) | 64 | 0.841 | 0.809 | 0.793 | 0.841 | 0.769 |  |  |  |
| P08581 (STY) | 36 | 0.857 | 0.835 | 0.835 | 0.857 | 0.808 |  |  |  |
| P08631 (STY) | 36 | 0.88 | 0.865 | 0.883 | 0.88 | 0.792 |  |  |  |
| P09215 (STY) | 23 | 0.855 | 0.824 | 0.816 | 0.855 | 0.715 |  |  |  |
| P09216 (STY) | 15 | 0.811 | 0.779 | 0.767 | 0.811 | 0.712 |  |  |  |
| P09217 (STY) | 22 | 0.826 | 0.777 | 0.736 | 0.826 | 0.78 |  |  |  |
| P09619 (STY) | 34 | 0.829 | 0.807 | 0.809 | 0.829 | 0.75 |  |  |  |
| P09769 (STY) | 19 | 0.823 | 0.786 | 0.763 | 0.823 | 0.75 |  |  |  |
| P0C605 (STY) | 26 | 0.886 | 0.874 | 0.882 | 0.886 | 0.922 |  |  |  |
| P11275 (STY) | 104 | 0.875 | 0.863 | 0.862 | 0.875 | 0.864 |  |  |  |
| P11309 (STY) | 64 | 0.888 | 0.874 | 0.882 | 0.888 | 0.812 |  |  |  |
| P11362 (STY) | 46 | 0.83 | 0.78 | 0.75 | 0.83 | 0.572 |  |  |  |
| P11440 (STY) | 136 | 0.957 | 0.957 | 0.96 | 0.957 | 0.954 |  |  |  |
| P11798 (STY) | 73 | 0.888 | 0.876 | 0.886 | 0.888 | 0.846 |  |  |  |
| P11802 (STY) | 78 | 0.94 | 0.942 | 0.949 | 0.94 | 0.959 |  |  |  |
| P12931 (STY) | 658 | 0.813 | 0.798 | 0.801 | 0.813 | 0.803 |  |  |  |
| P13234 (STY) | 17 | 0.834 | 0.814 | 0.809 | 0.834 | 0.922 |  |  |  |
| P15127 (STY) | 27 | 0.827 | 0.787 | 0.768 | 0.827 | 0.78 |  |  |  |
| P15208 (STY) | 26 | 0.897 | 0.868 | 0.856 | 0.897 | 0.814 |  |  |  |
| P16054 (STY) | 28 | 0.845 | 0.817 | 0.805 | 0.845 | 0.818 |  |  |  |
| P16092 (STY) | 18 | 0.835 | 0.796 | 0.771 | 0.835 | 0.667 |  |  |  |
| P16591 (STY) | 21 | 0.794 | 0.749 | 0.713 | 0.794 | 0.578 |  |  |  |
| P17157 (STY) | 20 | 0.958 | 0.96 | 0.973 | 0.958 | 0.97 |  |  |  |
| P17252 (STY) | 718 | 0.868 | 0.864 | 0.864 | 0.868 | 0.903 |  |  |  |
| P17612 (STY) | 930 | 0.909 | 0.907 | 0.908 | 0.909 | 0.949 |  |  |  |
| P18265 (STY) | 20 | 0.9 | 0.874 | 0.863 | 0.9 | 0.925 |  |  |  |
| P18266 (STY) | 62 | 0.911 | 0.901 | 0.898 | 0.911 | 0.896 |  |  |  |
| P18653 (STY) | 36 | 0.922 | 0.919 | 0.927 | 0.922 | 0.931 |  |  |  |
| P18654 (STY) | 25 | 0.86 | 0.844 | 0.835 | 0.86 | 0.84 |  |  |  |
| P19139 (STY) | 75 | 0.909 | 0.899 | 0.906 | 0.909 | 0.893 |  |  |  |
| P19525 (STY) | 25 | 0.82 | 0.767 | 0.725 | 0.82 | 0.61 |  |  |  |
| P19784 (STY) | 21 | 0.824 | 0.799 | 0.791 | 0.824 | 0.866 |  |  |  |
| P20444 (STY) | 103 | 0.871 | 0.853 | 0.865 | 0.871 | 0.857 |  |  |  |
| P21708 (STY) | 67 | 0.955 | 0.956 | 0.959 | 0.955 | 0.952 |  |  |  |
| P23443 (STY) | 59 | 0.912 | 0.906 | 0.91 | 0.912 | 0.934 |  |  |  |
| P23458 (STY) | 17 | 0.843 | 0.808 | 0.789 | 0.843 | 0.761 |  |  |  |
| P24723 (STY) | 18 | 0.835 | 0.804 | 0.787 | 0.835 | 0.683 |  |  |  |
| P24941 (STY) | 465 | 0.971 | 0.971 | 0.971 | 0.971 | 0.979 |  |  |  |
| P25098 (STY) | 118 | 0.863 | 0.843 | 0.856 | 0.863 | 0.837 |  |  |  |
| P25911 (STY) | 51 | 0.879 | 0.853 | 0.861 | 0.879 | 0.847 |  |  |  |
| P26927 (STY) | 15 | 0.833 | 0.786 | 0.757 | 0.833 | 0.557 |  |  |  |
| P27361 (STY) | 487 | 0.955 | 0.954 | 0.955 | 0.955 | 0.964 |  |  |  |
| P27791 (STY) | 143 | 0.909 | 0.903 | 0.904 | 0.909 | 0.912 |  |  |  |
| P28482 (STY) | 599 | 0.963 | 0.963 | 0.964 | 0.963 | 0.979 |  |  |  |
| P28867 (STY) | 29 | 0.88 | 0.858 | 0.871 | 0.88 | 0.822 |  |  |  |
| P29597 (STY) | 17 | 0.783 | 0.749 | 0.725 | 0.783 | 0.444 |  |  |  |
| P31749 (STY) | 411 | 0.956 | 0.955 | 0.956 | 0.956 | 0.971 |  |  |  |
| P31750 (STY) | 101 | 0.937 | 0.936 | 0.937 | 0.937 | 0.96 |  |  |  |
| P31751 (STY) | 79 | 0.875 | 0.862 | 0.873 | 0.875 | 0.876 |  |  |  |
| P32562 (STY) | 18 | 0.771 | 0.721 | 0.689 | 0.771 | 0.677 |  |  |  |
| P32577 (STY) | 22 | 0.796 | 0.746 | 0.703 | 0.796 | 0.495 |  |  |  |
| P33674 (STY) | 18 | 0.917 | 0.904 | 0.897 | 0.917 | 0.978 |  |  |  |
| P33981 (STY) | 66 | 0.823 | 0.778 | 0.76 | 0.823 | 0.701 |  |  |  |
| P34947 (STY) | 23 | 0.805 | 0.761 | 0.726 | 0.805 | 0.674 |  |  |  |
| P35465 (STY) | 25 | 0.86 | 0.839 | 0.825 | 0.86 | 0.796 |  |  |  |
| P35626 (STY) | 27 | 0.858 | 0.827 | 0.819 | 0.858 | 0.764 |  |  |  |
| P36887 (STY) | 20 | 0.858 | 0.841 | 0.845 | 0.858 | 0.825 |  |  |  |
| P37173 (STY) | 16 | 0.813 | 0.776 | 0.75 | 0.813 | 0.525 |  |  |  |
| P38110 (STY) | 19 | 0.955 | 0.955 | 0.962 | 0.955 | 0.978 |  |  |  |
| P38111 (STY) | 21 | 0.953 | 0.951 | 0.959 | 0.953 | 0.987 |  |  |  |
| P39688 (STY) | 65 | 0.833 | 0.794 | 0.768 | 0.833 | 0.723 |  |  |  |
| P39951 (STY) | 79 | 0.956 | 0.957 | 0.96 | 0.956 | 0.978 |  |  |  |
| P41240 (STY) | 28 | 0.881 | 0.837 | 0.82 | 0.881 | 0.604 |  |  |  |
| P41241 (STY) | 22 | 0.835 | 0.785 | 0.75 | 0.835 | 0.647 |  |  |  |
| P41279 (STY) | 25 | 0.88 | 0.852 | 0.842 | 0.88 | 0.711 |  |  |  |
| P41743 (STY) | 47 | 0.837 | 0.814 | 0.813 | 0.837 | 0.753 |  |  |  |
| P42345 (STY) | 142 | 0.885 | 0.873 | 0.882 | 0.885 | 0.827 |  |  |  |
| P42346 (STY) | 17 | 0.824 | 0.777 | 0.746 | 0.824 | 0.678 |  |  |  |
| P42684 (STY) | 33 | 0.823 | 0.808 | 0.81 | 0.823 | 0.658 |  |  |  |
| P43250 (STY) | 16 | 0.794 | 0.759 | 0.74 | 0.794 | 0.519 |  |  |  |
| P43403 (STY) | 39 | 0.863 | 0.833 | 0.854 | 0.863 | 0.778 |  |  |  |
| P43405 (STY) | 85 | 0.894 | 0.882 | 0.89 | 0.894 | 0.86 |  |  |  |
| P45983 (STY) | 229 | 0.964 | 0.964 | 0.965 | 0.964 | 0.971 |  |  |  |
| P45984 (STY) | 82 | 0.955 | 0.955 | 0.959 | 0.955 | 0.968 |  |  |  |
| P45985 (STY) | 17 | 0.92 | 0.899 | 0.884 | 0.92 | 0.835 |  |  |  |
| P46196 (STY) | 17 | 0.883 | 0.867 | 0.864 | 0.883 | 0.828 |  |  |  |
| P47196 (STY) | 49 | 0.952 | 0.952 | 0.955 | 0.952 | 0.98 |  |  |  |
| P47811 (STY) | 90 | 0.928 | 0.925 | 0.928 | 0.928 | 0.893 |  |  |  |
| P48025 (STY) | 38 | 0.873 | 0.848 | 0.862 | 0.873 | 0.803 |  |  |  |
| P48729 (STY) | 170 | 0.885 | 0.87 | 0.877 | 0.885 | 0.867 |  |  |  |
| P48730 (STY) | 105 | 0.871 | 0.858 | 0.864 | 0.871 | 0.851 |  |  |  |
| P48734 (STY) | 36 | 0.945 | 0.945 | 0.949 | 0.945 | 0.983 |  |  |  |
| P49137 (STY) | 101 | 0.926 | 0.921 | 0.926 | 0.926 | 0.915 |  |  |  |
| P49138 (STY) | 36 | 0.903 | 0.882 | 0.872 | 0.903 | 0.87 |  |  |  |
| P49185 (STY) | 42 | 0.92 | 0.92 | 0.927 | 0.92 | 0.953 |  |  |  |
| P49186 (STY) | 16 | 0.908 | 0.892 | 0.897 | 0.908 | 0.95 |  |  |  |
| P49336 (STY) | 18 | 0.945 | 0.932 | 0.928 | 0.945 | 0.889 |  |  |  |
| P49615 (STY) | 105 | 0.959 | 0.959 | 0.962 | 0.959 | 0.95 |  |  |  |
| P49674 (STY) | 60 | 0.847 | 0.819 | 0.818 | 0.847 | 0.781 |  |  |  |
| P49760 (STY) | 20 | 0.817 | 0.784 | 0.763 | 0.817 | 0.77 |  |  |  |
| P49840 (STY) | 110 | 0.938 | 0.937 | 0.94 | 0.938 | 0.959 |  |  |  |
| P49841 (STY) | 433 | 0.925 | 0.923 | 0.924 | 0.925 | 0.952 |  |  |  |
| P50613 (STY) | 60 | 0.867 | 0.85 | 0.858 | 0.867 | 0.786 |  |  |  |
| P50750 (STY) | 51 | 0.885 | 0.874 | 0.875 | 0.885 | 0.818 |  |  |  |
| P51812 (STY) | 72 | 0.891 | 0.881 | 0.888 | 0.891 | 0.861 |  |  |  |
| P51813 (STY) | 22 | 0.811 | 0.771 | 0.744 | 0.811 | 0.742 |  |  |  |
| P51955 (STY) | 35 | 0.81 | 0.765 | 0.737 | 0.81 | 0.637 |  |  |  |
| P52333 (STY) | 23 | 0.834 | 0.782 | 0.742 | 0.834 | 0.634 |  |  |  |
| P52564 (STY) | 15 | 0.922 | 0.914 | 0.922 | 0.922 | 0.886 |  |  |  |
| P53350 (STY) | 340 | 0.869 | 0.849 | 0.86 | 0.869 | 0.812 |  |  |  |
| P53351 (STY) | 24 | 0.82 | 0.776 | 0.749 | 0.82 | 0.611 |  |  |  |
| P53355 (STY) | 31 | 0.823 | 0.786 | 0.781 | 0.823 | 0.659 |  |  |  |
| P53778 (STY) | 41 | 0.947 | 0.947 | 0.954 | 0.947 | 0.987 |  |  |  |
| P53779 (STY) | 32 | 0.912 | 0.912 | 0.915 | 0.912 | 0.913 |  |  |  |
| P54199 (STY) | 18 | 0.751 | 0.689 | 0.639 | 0.751 | 0.436 |  |  |  |
| P54645 (STY) | 30 | 0.878 | 0.86 | 0.871 | 0.878 | 0.88 |  |  |  |
| P54646 (STY) | 47 | 0.869 | 0.85 | 0.864 | 0.869 | 0.841 |  |  |  |
| P57059 (STY) | 20 | 0.892 | 0.858 | 0.829 | 0.892 | 0.87 |  |  |  |
| P63085 (STY) | 267 | 0.966 | 0.966 | 0.967 | 0.966 | 0.977 |  |  |  |
| P63086 (STY) | 90 | 0.959 | 0.959 | 0.961 | 0.959 | 0.927 |  |  |  |
| P67870 (STY) | 20 | 0.917 | 0.911 | 0.923 | 0.917 | 0.98 |  |  |  |
| P67999 (STY) | 21 | 0.937 | 0.93 | 0.941 | 0.937 | 0.913 |  |  |  |
| P68399 (STY) | 16 | 0.94 | 0.937 | 0.942 | 0.94 | 0.95 |  |  |  |
| P68400 (STY) | 746 | 0.922 | 0.921 | 0.922 | 0.922 | 0.958 |  |  |  |
| P68403 (STY) | 25 | 0.82 | 0.784 | 0.76 | 0.82 | 0.776 |  |  |  |
| P68404 (STY) | 22 | 0.881 | 0.848 | 0.829 | 0.881 | 0.841 |  |  |  |
| P70032 (STY) | 15 | 0.811 | 0.753 | 0.707 | 0.811 | 0.664 |  |  |  |
| P70335 (STY) | 27 | 0.932 | 0.924 | 0.939 | 0.932 | 0.802 |  |  |  |
| P70336 (STY) | 27 | 0.87 | 0.841 | 0.83 | 0.87 | 0.751 |  |  |  |
| P70618 (STY) | 21 | 0.874 | 0.871 | 0.891 | 0.874 | 0.915 |  |  |  |
| P78527 (STY) | 136 | 0.914 | 0.907 | 0.912 | 0.914 | 0.887 |  |  |  |
| P97313 (STY) | 17 | 0.844 | 0.802 | 0.772 | 0.844 | 0.851 |  |  |  |
| P97377 (STY) | 72 | 0.926 | 0.925 | 0.93 | 0.926 | 0.951 |  |  |  |
| P97633 (STY) | 15 | 0.8 | 0.748 | 0.707 | 0.8 | 0.57 |  |  |  |
| Q00526 (STY) | 16 | 0.918 | 0.906 | 0.904 | 0.918 | 0.95 |  |  |  |
| Q00534 (STY) | 41 | 0.935 | 0.936 | 0.941 | 0.935 | 0.98 |  |  |  |
| Q00535 (STY) | 246 | 0.969 | 0.969 | 0.97 | 0.969 | 0.972 |  |  |  |
| Q01279 (STY) | 26 | 0.833 | 0.799 | 0.774 | 0.833 | 0.758 |  |  |  |
| Q01314 (STY) | 20 | 0.917 | 0.915 | 0.927 | 0.917 | 0.96 |  |  |  |
| Q02111 (STY) | 17 | 0.843 | 0.799 | 0.773 | 0.843 | 0.603 |  |  |  |
| Q02156 (STY) | 113 | 0.878 | 0.863 | 0.866 | 0.878 | 0.808 |  |  |  |
| Q02399 (STY) | 16 | 0.95 | 0.947 | 0.96 | 0.95 | 0.944 |  |  |  |
| Q02956 (STY) | 34 | 0.864 | 0.837 | 0.833 | 0.864 | 0.832 |  |  |  |
| Q03114 (STY) | 73 | 0.959 | 0.959 | 0.962 | 0.959 | 0.965 |  |  |  |
| Q04759 (STY) | 61 | 0.864 | 0.842 | 0.842 | 0.864 | 0.793 |  |  |  |
| Q05397 (STY) | 29 | 0.827 | 0.791 | 0.772 | 0.827 | 0.765 |  |  |  |
| Q05513 (STY) | 121 | 0.844 | 0.811 | 0.814 | 0.844 | 0.751 |  |  |  |
| Q05655 (STY) | 177 | 0.866 | 0.847 | 0.852 | 0.866 | 0.82 |  |  |  |
| Q06187 (STY) | 26 | 0.847 | 0.813 | 0.8 | 0.847 | 0.709 |  |  |  |
| Q06226 (STY) | 15 | 0.9 | 0.875 | 0.862 | 0.9 | 0.83 |  |  |  |
| Q07014 (STY) | 18 | 0.825 | 0.78 | 0.754 | 0.825 | 0.783 |  |  |  |
| Q07832 (STY) | 49 | 0.844 | 0.798 | 0.782 | 0.844 | 0.787 |  |  |  |
| Q08881 (STY) | 15 | 0.8 | 0.741 | 0.693 | 0.8 | 0.461 |  |  |  |
| Q09137 (STY) | 35 | 0.867 | 0.845 | 0.857 | 0.867 | 0.849 |  |  |  |
| Q13043 (STY) | 34 | 0.819 | 0.78 | 0.751 | 0.819 | 0.628 |  |  |  |
| Q13131 (STY) | 203 | 0.896 | 0.884 | 0.889 | 0.896 | 0.883 |  |  |  |
| Q13153 (STY) | 91 | 0.867 | 0.856 | 0.862 | 0.867 | 0.856 |  |  |  |
| Q13164 (STY) | 32 | 0.886 | 0.868 | 0.871 | 0.886 | 0.914 |  |  |  |
| Q13177 (STY) | 50 | 0.833 | 0.796 | 0.773 | 0.833 | 0.746 |  |  |  |
| Q13188 (STY) | 28 | 0.816 | 0.792 | 0.778 | 0.816 | 0.66 |  |  |  |
| Q13237 (STY) | 29 | 0.919 | 0.912 | 0.932 | 0.919 | 0.957 |  |  |  |
| Q13315 (STY) | 321 | 0.957 | 0.957 | 0.958 | 0.957 | 0.976 |  |  |  |
| Q13464 (STY) | 86 | 0.89 | 0.874 | 0.871 | 0.89 | 0.902 |  |  |  |
| Q13535 (STY) | 124 | 0.952 | 0.952 | 0.953 | 0.952 | 0.966 |  |  |  |
| Q13554 (STY) | 22 | 0.909 | 0.898 | 0.91 | 0.909 | 0.829 |  |  |  |
| Q13557 (STY) | 26 | 0.878 | 0.861 | 0.876 | 0.878 | 0.819 |  |  |  |
| Q13627 (STY) | 50 | 0.903 | 0.903 | 0.909 | 0.903 | 0.931 |  |  |  |
| Q13882 (STY) | 26 | 0.821 | 0.778 | 0.757 | 0.821 | 0.521 |  |  |  |
| Q13976 (STY) | 84 | 0.915 | 0.909 | 0.917 | 0.915 | 0.918 |  |  |  |
| Q14012 (STY) | 42 | 0.924 | 0.917 | 0.924 | 0.924 | 0.873 |  |  |  |
| Q14164 (STY) | 48 | 0.823 | 0.79 | 0.789 | 0.823 | 0.734 |  |  |  |
| Q14289 (STY) | 20 | 0.817 | 0.765 | 0.725 | 0.817 | 0.645 |  |  |  |
| Q14680 (STY) | 29 | 0.798 | 0.751 | 0.718 | 0.798 | 0.642 |  |  |  |
| Q15046 (STY) | 38 | 0.882 | 0.864 | 0.86 | 0.882 | 0.891 |  |  |  |
| Q15118 (STY) | 31 | 0.897 | 0.885 | 0.882 | 0.897 | 0.924 |  |  |  |
| Q15139 (STY) | 90 | 0.926 | 0.917 | 0.927 | 0.926 | 0.917 |  |  |  |
| Q15418 (STY) | 109 | 0.925 | 0.921 | 0.924 | 0.925 | 0.933 |  |  |  |
| Q15759 (STY) | 51 | 0.938 | 0.937 | 0.94 | 0.938 | 0.942 |  |  |  |
| Q15831 (STY) | 37 | 0.883 | 0.851 | 0.862 | 0.883 | 0.738 |  |  |  |
| Q15835 (STY) | 19 | 0.823 | 0.807 | 0.809 | 0.823 | 0.753 |  |  |  |
| Q16512 (STY) | 16 | 0.836 | 0.804 | 0.786 | 0.836 | 0.912 |  |  |  |
| Q16539 (STY) | 301 | 0.936 | 0.935 | 0.935 | 0.936 | 0.95 |  |  |  |
| Q16566 (STY) | 24 | 0.91 | 0.889 | 0.893 | 0.91 | 0.858 |  |  |  |
| Q16620 (STY) | 17 | 0.834 | 0.777 | 0.737 | 0.834 | 0.783 |  |  |  |
| Q2MHE4 (STY) | 40 | 0.636 | 0.622 | 0.66 | 0.636 | 0.729 |  |  |  |
| Q2TA25 (STY) | 16 | 0.814 | 0.75 | 0.697 | 0.814 | 0.481 |  |  |  |
| Q39011 (STY) | 36 | 0.545 | 0.523 | 0.537 | 0.545 | 0.469 |  |  |  |
| Q3SYZ2 (STY) | 16 | 0.827 | 0.789 | 0.762 | 0.827 | 0.694 |  |  |  |
| Q5EG47 (STY) | 36 | 0.884 | 0.872 | 0.884 | 0.884 | 0.864 |  |  |  |
| Q5RCH1 (STY) | 17 | 0.98 | 0.982 | 0.988 | 0.98 | 0.988 |  |  |  |
| Q5S007 (STY) | 94 | 0.847 | 0.808 | 0.819 | 0.847 | 0.696 |  |  |  |
| Q60670 (STY) | 15 | 0.9 | 0.879 | 0.881 | 0.9 | 0.952 |  |  |  |
| Q60680 (STY) | 18 | 0.833 | 0.8 | 0.786 | 0.833 | 0.706 |  |  |  |
| Q60737 (STY) | 68 | 0.892 | 0.883 | 0.884 | 0.892 | 0.915 |  |  |  |
| Q60806 (STY) | 16 | 0.834 | 0.782 | 0.742 | 0.834 | 0.619 |  |  |  |
| Q60823 (STY) | 21 | 0.889 | 0.879 | 0.886 | 0.889 | 0.935 |  |  |  |
| Q61036 (STY) | 17 | 0.884 | 0.836 | 0.798 | 0.884 | 0.648 |  |  |  |
| Q61831 (STY) | 20 | 0.925 | 0.922 | 0.934 | 0.925 | 0.965 |  |  |  |
| Q62101 (STY) | 19 | 0.93 | 0.918 | 0.919 | 0.93 | 0.903 |  |  |  |
| Q62120 (STY) | 48 | 0.792 | 0.75 | 0.724 | 0.792 | 0.524 |  |  |  |
| Q62388 (STY) | 49 | 0.932 | 0.933 | 0.94 | 0.932 | 0.921 |  |  |  |
| Q62689 (STY) | 22 | 0.842 | 0.798 | 0.774 | 0.842 | 0.712 |  |  |  |
| Q62844 (STY) | 22 | 0.842 | 0.789 | 0.751 | 0.842 | 0.673 |  |  |  |
| Q62868 (STY) | 20 | 0.833 | 0.791 | 0.758 | 0.833 | 0.655 |  |  |  |
| Q63450 (STY) | 18 | 0.9 | 0.88 | 0.873 | 0.9 | 0.872 |  |  |  |
| Q63470 (STY) | 23 | 0.833 | 0.804 | 0.785 | 0.833 | 0.677 |  |  |  |
| Q63531 (STY) | 27 | 0.908 | 0.894 | 0.901 | 0.908 | 0.9 |  |  |  |
| Q63644 (STY) | 17 | 0.845 | 0.821 | 0.811 | 0.845 | 0.779 |  |  |  |
| Q63699 (STY) | 20 | 0.942 | 0.943 | 0.954 | 0.942 | 0.9 |  |  |  |
| Q63844 (STY) | 107 | 0.952 | 0.952 | 0.953 | 0.952 | 0.939 |  |  |  |
| Q64303 (STY) | 15 | 0.844 | 0.797 | 0.762 | 0.844 | 0.53 |  |  |  |
| Q64702 (STY) | 16 | 0.867 | 0.817 | 0.78 | 0.867 | 0.75 |  |  |  |
| Q7KZI7 (STY) | 31 | 0.877 | 0.856 | 0.853 | 0.877 | 0.706 |  |  |  |
| Q8BSK8 (STY) | 29 | 0.919 | 0.91 | 0.924 | 0.919 | 0.895 |  |  |  |
| Q8C050 (STY) | 19 | 0.886 | 0.872 | 0.87 | 0.886 | 0.896 |  |  |  |
| Q8CIN4 (STY) | 17 | 0.844 | 0.796 | 0.763 | 0.844 | 0.637 |  |  |  |
| Q8IW41 (STY) | 18 | 0.882 | 0.865 | 0.863 | 0.882 | 0.85 |  |  |  |
| Q8IXL6 (STY) | 196 | 0.893 | 0.883 | 0.889 | 0.893 | 0.855 |  |  |  |
| Q8N5S9 (STY) | 15 | 0.9 | 0.869 | 0.849 | 0.9 | 0.771 |  |  |  |
| Q91Y86 (STY) | 77 | 0.933 | 0.933 | 0.936 | 0.933 | 0.928 |  |  |  |
| Q91YS8 (STY) | 16 | 0.886 | 0.853 | 0.837 | 0.886 | 0.862 |  |  |  |
| Q92630 (STY) | 37 | 0.946 | 0.943 | 0.95 | 0.946 | 0.924 |  |  |  |
| Q94F62 (STY) | 23 | 0.681 | 0.627 | 0.586 | 0.681 | 0.377 |  |  |  |
| Q96GD4 (STY) | 191 | 0.912 | 0.907 | 0.909 | 0.912 | 0.912 |  |  |  |
| Q96QT4 (STY) | 63 | 0.811 | 0.762 | 0.749 | 0.811 | 0.57 |  |  |  |
| Q96SB4 (STY) | 23 | 0.946 | 0.943 | 0.957 | 0.946 | 0.952 |  |  |  |
| Q99683 (STY) | 28 | 0.846 | 0.823 | 0.831 | 0.846 | 0.844 |  |  |  |
| Q99986 (STY) | 18 | 0.889 | 0.859 | 0.848 | 0.889 | 0.678 |  |  |  |
| Q9BXM7 (STY) | 17 | 0.803 | 0.76 | 0.73 | 0.803 | 0.533 |  |  |  |
| Q9BZL6 (STY) | 20 | 0.917 | 0.908 | 0.91 | 0.917 | 0.905 |  |  |  |
| Q9DC28 (STY) | 53 | 0.852 | 0.825 | 0.832 | 0.852 | 0.724 |  |  |  |
| Q9H0K1 (STY) | 17 | 0.912 | 0.889 | 0.882 | 0.912 | 0.85 |  |  |  |
| Q9H2X6 (STY) | 68 | 0.941 | 0.942 | 0.946 | 0.941 | 0.916 |  |  |  |
| Q9H4B4 (STY) | 34 | 0.824 | 0.786 | 0.761 | 0.824 | 0.732 |  |  |  |
| Q9HC98 (STY) | 23 | 0.827 | 0.805 | 0.79 | 0.827 | 0.806 |  |  |  |
| Q9JKK8 (STY) | 15 | 1.0 | 1.0 | 1.0 | 1.0 | 1.0 |  |  |  |
| Q9JLN9 (STY) | 104 | 0.899 | 0.892 | 0.893 | 0.899 | 0.876 |  |  |  |
| Q9NRM7 (STY) | 19 | 0.939 | 0.935 | 0.941 | 0.939 | 0.989 |  |  |  |
| Q9NWZ3 (STY) | 34 | 0.638 | 0.597 | 0.567 | 0.638 | 0.488 |  |  |  |
| Q9NYY3 (STY) | 33 | 0.813 | 0.755 | 0.71 | 0.813 | 0.596 |  |  |  |
| Q9P1W9 (STY) | 18 | 0.888 | 0.873 | 0.882 | 0.888 | 0.706 |  |  |  |
| Q9QZR5 (STY) | 23 | 0.92 | 0.917 | 0.94 | 0.92 | 0.898 |  |  |  |
| Q9R012 (STY) | 24 | 0.813 | 0.755 | 0.706 | 0.813 | 0.628 |  |  |  |
| Q9UBE8 (STY) | 16 | 0.853 | 0.834 | 0.826 | 0.853 | 0.919 |  |  |  |
| Q9UEW8 (STY) | 20 | 0.883 | 0.863 | 0.859 | 0.883 | 0.865 |  |  |  |
| Q9UHD2 (STY) | 88 | 0.843 | 0.808 | 0.815 | 0.843 | 0.697 |  |  |  |
| Q9UM73 (STY) | 15 | 0.778 | 0.727 | 0.69 | 0.778 | 0.686 |  |  |  |
| Q9UQM7 (STY) | 279 | 0.878 | 0.866 | 0.867 | 0.878 | 0.883 |  |  |  |
| Q9WTK7 (STY) | 17 | 0.931 | 0.91 | 0.902 | 0.931 | 0.801 |  |  |  |
| Q9WTU6 (STY) | 30 | 0.939 | 0.937 | 0.944 | 0.939 | 0.956 |  |  |  |
| Q9WUD9 (STY) | 76 | 0.838 | 0.806 | 0.802 | 0.838 | 0.72 |  |  |  |
| Q9WV60 (STY) | 104 | 0.901 | 0.897 | 0.9 | 0.901 | 0.906 |  |  |  |
| Q9WVC6 (STY) | 32 | 0.933 | 0.928 | 0.941 | 0.933 | 0.976 |  |  |  |
| Q9Y478 (STY) | 57 | 0.889 | 0.879 | 0.879 | 0.889 | 0.876 |  |  |  |
| Q9Z2A0 (STY) | 35 | 0.924 | 0.906 | 0.895 | 0.924 | 0.906 |  |  |  |
| A6QLB8 (ST) | 16 | 0.775 | 0.758 | 0.777 | 0.775 | 0.85 |  |  |  |
| G3N1T2 (ST) | 24 | 0.869 | 0.846 | 0.851 | 0.869 | 0.775 |  |  |  |
| O00141 (ST) | 76 | 0.954 | 0.953 | 0.955 | 0.954 | 0.967 |  |  |  |
| O00311 (ST) | 48 | 0.844 | 0.815 | 0.825 | 0.844 | 0.738 |  |  |  |
| O00418 (ST) | 16 | 0.843 | 0.824 | 0.826 | 0.843 | 0.797 |  |  |  |
| O14757 (ST) | 201 | 0.894 | 0.886 | 0.886 | 0.894 | 0.854 |  |  |  |
| O14920 (ST) | 96 | 0.826 | 0.789 | 0.797 | 0.826 | 0.724 |  |  |  |
| O14965 (ST) | 144 | 0.895 | 0.887 | 0.889 | 0.895 | 0.849 |  |  |  |
| O15111 (ST) | 56 | 0.822 | 0.777 | 0.756 | 0.822 | 0.665 |  |  |  |
| O15264 (ST) | 38 | 0.935 | 0.929 | 0.926 | 0.935 | 0.909 |  |  |  |
| O15530 (ST) | 76 | 0.908 | 0.898 | 0.901 | 0.908 | 0.906 |  |  |  |
| O43293 (ST) | 24 | 0.876 | 0.85 | 0.836 | 0.876 | 0.788 |  |  |  |
| O43318 (ST) | 43 | 0.872 | 0.848 | 0.846 | 0.872 | 0.796 |  |  |  |
| O43683 (ST) | 27 | 0.673 | 0.631 | 0.615 | 0.673 | 0.537 |  |  |  |
| O55099 (ST) | 18 | 0.86 | 0.858 | 0.884 | 0.86 | 0.822 |  |  |  |
| O55173 (ST) | 31 | 0.945 | 0.931 | 0.936 | 0.945 | 0.958 |  |  |  |
| O70126 (ST) | 34 | 0.887 | 0.877 | 0.894 | 0.887 | 0.901 |  |  |  |
| O70405 (ST) | 17 | 0.795 | 0.738 | 0.692 | 0.795 | 0.569 |  |  |  |
| O75116 (ST) | 48 | 0.882 | 0.86 | 0.858 | 0.882 | 0.803 |  |  |  |
| O75385 (ST) | 80 | 0.838 | 0.805 | 0.795 | 0.838 | 0.706 |  |  |  |
| O75582 (ST) | 33 | 0.894 | 0.883 | 0.879 | 0.894 | 0.877 |  |  |  |
| O88351 (ST) | 39 | 0.85 | 0.812 | 0.801 | 0.85 | 0.644 |  |  |  |
| O88643 (ST) | 22 | 0.84 | 0.798 | 0.763 | 0.84 | 0.677 |  |  |  |
| O95747 (ST) | 15 | 0.9 | 0.889 | 0.884 | 0.9 | 0.846 |  |  |  |
| O95835 (ST) | 17 | 0.931 | 0.926 | 0.926 | 0.931 | 0.761 |  |  |  |
| O96013 (ST) | 21 | 0.856 | 0.826 | 0.811 | 0.856 | 0.843 |  |  |  |
| O96017 (ST) | 74 | 0.872 | 0.851 | 0.861 | 0.872 | 0.839 |  |  |  |
| P00516 (ST) | 54 | 0.92 | 0.908 | 0.923 | 0.92 | 0.855 |  |  |  |
| P00517 (ST) | 216 | 0.92 | 0.917 | 0.916 | 0.92 | 0.943 |  |  |  |
| P00546 (ST) | 93 | 0.836 | 0.814 | 0.823 | 0.836 | 0.765 |  |  |  |
| P04049 (ST) | 20 | 0.85 | 0.815 | 0.797 | 0.85 | 0.745 |  |  |  |
| P04409 (ST) | 59 | 0.87 | 0.853 | 0.856 | 0.87 | 0.897 |  |  |  |
| P04551 (ST) | 38 | 0.965 | 0.966 | 0.97 | 0.965 | 0.997 |  |  |  |
| P05129 (ST) | 56 | 0.863 | 0.839 | 0.857 | 0.863 | 0.812 |  |  |  |
| P05132 (ST) | 193 | 0.938 | 0.935 | 0.937 | 0.938 | 0.942 |  |  |  |
| P05696 (ST) | 187 | 0.878 | 0.865 | 0.867 | 0.878 | 0.863 |  |  |  |
| P05771 (ST) | 101 | 0.851 | 0.832 | 0.834 | 0.851 | 0.769 |  |  |  |
| P06493 (ST) | 760 | 0.962 | 0.962 | 0.964 | 0.962 | 0.975 |  |  |  |
| P08413 (ST) | 64 | 0.828 | 0.794 | 0.778 | 0.828 | 0.748 |  |  |  |
| P09215 (ST) | 23 | 0.848 | 0.817 | 0.807 | 0.848 | 0.739 |  |  |  |
| P09216 (ST) | 15 | 0.778 | 0.737 | 0.72 | 0.778 | 0.648 |  |  |  |
| P09217 (ST) | 22 | 0.819 | 0.768 | 0.732 | 0.819 | 0.752 |  |  |  |
| P0C605 (ST) | 26 | 0.93 | 0.927 | 0.944 | 0.93 | 0.915 |  |  |  |
| P11275 (ST) | 104 | 0.875 | 0.864 | 0.865 | 0.875 | 0.856 |  |  |  |
| P11309 (ST) | 64 | 0.867 | 0.853 | 0.856 | 0.867 | 0.783 |  |  |  |
| P11440 (ST) | 135 | 0.968 | 0.968 | 0.969 | 0.968 | 0.966 |  |  |  |
| P11798 (ST) | 73 | 0.884 | 0.866 | 0.879 | 0.884 | 0.812 |  |  |  |
| P11802 (ST) | 77 | 0.946 | 0.949 | 0.958 | 0.946 | 0.964 |  |  |  |
| P13234 (ST) | 17 | 0.843 | 0.821 | 0.821 | 0.843 | 0.843 |  |  |  |
| P16054 (ST) | 28 | 0.803 | 0.77 | 0.752 | 0.803 | 0.613 |  |  |  |
| P17157 (ST) | 20 | 0.892 | 0.886 | 0.899 | 0.892 | 0.97 |  |  |  |
| P17252 (ST) | 718 | 0.862 | 0.86 | 0.86 | 0.862 | 0.908 |  |  |  |
| P17612 (ST) | 929 | 0.915 | 0.914 | 0.916 | 0.915 | 0.959 |  |  |  |
| P18265 (ST) | 20 | 0.875 | 0.853 | 0.849 | 0.875 | 0.86 |  |  |  |
| P18266 (ST) | 62 | 0.914 | 0.903 | 0.914 | 0.914 | 0.893 |  |  |  |
| P18653 (ST) | 36 | 0.926 | 0.921 | 0.93 | 0.926 | 0.907 |  |  |  |
| P18654 (ST) | 25 | 0.88 | 0.875 | 0.888 | 0.88 | 0.862 |  |  |  |
| P19139 (ST) | 75 | 0.922 | 0.916 | 0.923 | 0.922 | 0.909 |  |  |  |
| P19525 (ST) | 21 | 0.795 | 0.759 | 0.735 | 0.795 | 0.571 |  |  |  |
| P19784 (ST) | 21 | 0.898 | 0.88 | 0.872 | 0.898 | 0.914 |  |  |  |
| P20444 (ST) | 103 | 0.874 | 0.853 | 0.868 | 0.874 | 0.843 |  |  |  |
| P21708 (ST) | 67 | 0.948 | 0.948 | 0.953 | 0.948 | 0.959 |  |  |  |
| P23443 (ST) | 59 | 0.921 | 0.915 | 0.921 | 0.921 | 0.932 |  |  |  |
| P24723 (ST) | 18 | 0.825 | 0.78 | 0.748 | 0.825 | 0.644 |  |  |  |
| P24941 (ST) | 465 | 0.98 | 0.979 | 0.98 | 0.98 | 0.982 |  |  |  |
| P25098 (ST) | 118 | 0.857 | 0.839 | 0.84 | 0.857 | 0.841 |  |  |  |
| P26927 (ST) | 15 | 0.844 | 0.805 | 0.772 | 0.844 | 0.55 |  |  |  |
| P27361 (ST) | 484 | 0.963 | 0.962 | 0.963 | 0.963 | 0.975 |  |  |  |
| P27791 (ST) | 143 | 0.923 | 0.917 | 0.921 | 0.923 | 0.924 |  |  |  |
| P28482 (ST) | 597 | 0.968 | 0.968 | 0.968 | 0.968 | 0.98 |  |  |  |
| P28867 (ST) | 29 | 0.845 | 0.821 | 0.815 | 0.845 | 0.843 |  |  |  |
| P31749 (ST) | 411 | 0.946 | 0.945 | 0.945 | 0.946 | 0.974 |  |  |  |
| P31750 (ST) | 101 | 0.955 | 0.954 | 0.957 | 0.955 | 0.966 |  |  |  |
| P31751 (ST) | 79 | 0.903 | 0.894 | 0.902 | 0.903 | 0.907 |  |  |  |
| P32562 (ST) | 18 | 0.76 | 0.721 | 0.695 | 0.76 | 0.735 |  |  |  |
| P33674 (ST) | 18 | 0.955 | 0.953 | 0.959 | 0.955 | 0.989 |  |  |  |
| P33981 (ST) | 65 | 0.826 | 0.775 | 0.752 | 0.826 | 0.669 |  |  |  |
| P34947 (ST) | 23 | 0.813 | 0.784 | 0.773 | 0.813 | 0.686 |  |  |  |
| P35465 (ST) | 25 | 0.893 | 0.878 | 0.874 | 0.893 | 0.821 |  |  |  |
| P35626 (ST) | 27 | 0.846 | 0.817 | 0.81 | 0.846 | 0.753 |  |  |  |
| P36887 (ST) | 20 | 0.842 | 0.811 | 0.792 | 0.842 | 0.91 |  |  |  |
| P38110 (ST) | 19 | 0.982 | 0.983 | 0.988 | 0.982 | 0.989 |  |  |  |
| P38111 (ST) | 21 | 0.976 | 0.978 | 0.984 | 0.976 | 0.995 |  |  |  |
| P39951 (ST) | 79 | 0.947 | 0.947 | 0.949 | 0.947 | 0.983 |  |  |  |
| P41279 (ST) | 25 | 0.853 | 0.817 | 0.8 | 0.853 | 0.745 |  |  |  |
| P41743 (ST) | 47 | 0.837 | 0.805 | 0.814 | 0.837 | 0.759 |  |  |  |
| P42345 (ST) | 142 | 0.872 | 0.858 | 0.86 | 0.872 | 0.842 |  |  |  |
| P42346 (ST) | 17 | 0.832 | 0.793 | 0.772 | 0.832 | 0.7 |  |  |  |
| P43250 (ST) | 16 | 0.827 | 0.787 | 0.753 | 0.827 | 0.625 |  |  |  |
| P45983 (ST) | 229 | 0.974 | 0.974 | 0.975 | 0.974 | 0.977 |  |  |  |
| P45984 (ST) | 82 | 0.955 | 0.954 | 0.956 | 0.955 | 0.971 |  |  |  |
| P46196 (ST) | 17 | 0.795 | 0.783 | 0.785 | 0.795 | 0.797 |  |  |  |
| P47196 (ST) | 49 | 0.946 | 0.946 | 0.951 | 0.946 | 0.98 |  |  |  |
| P47811 (ST) | 89 | 0.914 | 0.91 | 0.914 | 0.914 | 0.927 |  |  |  |
| P48729 (ST) | 169 | 0.895 | 0.882 | 0.894 | 0.895 | 0.881 |  |  |  |
| P48730 (ST) | 105 | 0.879 | 0.862 | 0.882 | 0.879 | 0.848 |  |  |  |
| P48734 (ST) | 36 | 0.968 | 0.968 | 0.973 | 0.968 | 0.987 |  |  |  |
| P49137 (ST) | 101 | 0.926 | 0.921 | 0.929 | 0.926 | 0.91 |  |  |  |
| P49138 (ST) | 36 | 0.907 | 0.896 | 0.915 | 0.907 | 0.894 |  |  |  |
| P49185 (ST) | 42 | 0.917 | 0.914 | 0.921 | 0.917 | 0.957 |  |  |  |
| P49186 (ST) | 16 | 0.938 | 0.928 | 0.929 | 0.938 | 0.912 |  |  |  |
| P49336 (ST) | 18 | 0.899 | 0.892 | 0.9 | 0.899 | 0.867 |  |  |  |
| P49615 (ST) | 105 | 0.959 | 0.959 | 0.961 | 0.959 | 0.948 |  |  |  |
| P49674 (ST) | 60 | 0.856 | 0.833 | 0.839 | 0.856 | 0.856 |  |  |  |
| P49760 (ST) | 20 | 0.782 | 0.742 | 0.715 | 0.782 | 0.876 |  |  |  |
| P49840 (ST) | 109 | 0.959 | 0.958 | 0.959 | 0.959 | 0.963 |  |  |  |
| P49841 (ST) | 432 | 0.931 | 0.929 | 0.93 | 0.931 | 0.963 |  |  |  |
| P50613 (ST) | 59 | 0.864 | 0.849 | 0.851 | 0.864 | 0.751 |  |  |  |
| P50750 (ST) | 51 | 0.905 | 0.893 | 0.891 | 0.905 | 0.853 |  |  |  |
| P51812 (ST) | 72 | 0.914 | 0.904 | 0.919 | 0.914 | 0.882 |  |  |  |
| P51955 (ST) | 35 | 0.771 | 0.735 | 0.704 | 0.771 | 0.572 |  |  |  |
| P53350 (ST) | 340 | 0.851 | 0.835 | 0.844 | 0.851 | 0.836 |  |  |  |
| P53351 (ST) | 24 | 0.786 | 0.741 | 0.701 | 0.786 | 0.474 |  |  |  |
| P53355 (ST) | 31 | 0.844 | 0.819 | 0.804 | 0.844 | 0.695 |  |  |  |
| P53778 (ST) | 41 | 0.931 | 0.928 | 0.935 | 0.931 | 0.961 |  |  |  |
| P53779 (ST) | 32 | 0.917 | 0.913 | 0.923 | 0.917 | 0.894 |  |  |  |
| P54199 (ST) | 18 | 0.762 | 0.695 | 0.647 | 0.762 | 0.504 |  |  |  |
| P54645 (ST) | 30 | 0.861 | 0.852 | 0.862 | 0.861 | 0.882 |  |  |  |
| P54646 (ST) | 47 | 0.876 | 0.854 | 0.866 | 0.876 | 0.837 |  |  |  |
| P57059 (ST) | 20 | 0.883 | 0.861 | 0.851 | 0.883 | 0.795 |  |  |  |
| P63085 (ST) | 265 | 0.97 | 0.97 | 0.971 | 0.97 | 0.981 |  |  |  |
| P63086 (ST) | 89 | 0.949 | 0.95 | 0.951 | 0.949 | 0.95 |  |  |  |
| P67870 (ST) | 20 | 0.908 | 0.9 | 0.904 | 0.908 | 0.895 |  |  |  |
| P67999 (ST) | 21 | 0.937 | 0.928 | 0.945 | 0.937 | 0.92 |  |  |  |
| P68399 (ST) | 16 | 0.94 | 0.937 | 0.942 | 0.94 | 0.969 |  |  |  |
| P68400 (ST) | 742 | 0.921 | 0.919 | 0.921 | 0.921 | 0.961 |  |  |  |
| P68403 (ST) | 25 | 0.807 | 0.774 | 0.752 | 0.807 | 0.691 |  |  |  |
| P68404 (ST) | 22 | 0.858 | 0.825 | 0.808 | 0.858 | 0.847 |  |  |  |
| P70032 (ST) | 15 | 0.811 | 0.757 | 0.712 | 0.811 | 0.529 |  |  |  |
| P70335 (ST) | 27 | 0.845 | 0.826 | 0.819 | 0.845 | 0.738 |  |  |  |
| P70336 (ST) | 27 | 0.865 | 0.828 | 0.816 | 0.865 | 0.742 |  |  |  |
| P70618 (ST) | 21 | 0.906 | 0.901 | 0.915 | 0.906 | 0.954 |  |  |  |
| P78527 (ST) | 136 | 0.911 | 0.904 | 0.909 | 0.911 | 0.899 |  |  |  |
| P97313 (ST) | 17 | 0.833 | 0.782 | 0.747 | 0.833 | 0.794 |  |  |  |
| P97377 (ST) | 72 | 0.935 | 0.936 | 0.939 | 0.935 | 0.956 |  |  |  |
| P97633 (ST) | 15 | 0.8 | 0.751 | 0.713 | 0.8 | 0.648 |  |  |  |
| Q00526 (ST) | 16 | 0.917 | 0.916 | 0.928 | 0.917 | 0.944 |  |  |  |
| Q00534 (ST) | 41 | 0.942 | 0.944 | 0.955 | 0.942 | 0.967 |  |  |  |
| Q00535 (ST) | 246 | 0.97 | 0.97 | 0.971 | 0.97 | 0.969 |  |  |  |
| Q01314 (ST) | 20 | 0.925 | 0.918 | 0.923 | 0.925 | 0.975 |  |  |  |
| Q02111 (ST) | 17 | 0.854 | 0.805 | 0.767 | 0.854 | 0.591 |  |  |  |
| Q02156 (ST) | 113 | 0.875 | 0.86 | 0.866 | 0.875 | 0.809 |  |  |  |
| Q02399 (ST) | 16 | 0.896 | 0.892 | 0.913 | 0.896 | 0.931 |  |  |  |
| Q02956 (ST) | 34 | 0.868 | 0.843 | 0.86 | 0.868 | 0.8 |  |  |  |
| Q03114 (ST) | 73 | 0.97 | 0.971 | 0.976 | 0.97 | 0.979 |  |  |  |
| Q04759 (ST) | 57 | 0.903 | 0.892 | 0.899 | 0.903 | 0.817 |  |  |  |
| Q05513 (ST) | 121 | 0.842 | 0.806 | 0.816 | 0.842 | 0.713 |  |  |  |
| Q05655 (ST) | 175 | 0.867 | 0.849 | 0.859 | 0.867 | 0.834 |  |  |  |
| Q06226 (ST) | 15 | 0.889 | 0.852 | 0.826 | 0.889 | 0.777 |  |  |  |
| Q07832 (ST) | 49 | 0.847 | 0.808 | 0.79 | 0.847 | 0.744 |  |  |  |
| Q09137 (ST) | 35 | 0.876 | 0.857 | 0.883 | 0.876 | 0.884 |  |  |  |
| Q13043 (ST) | 34 | 0.844 | 0.811 | 0.797 | 0.844 | 0.754 |  |  |  |
| Q13131 (ST) | 203 | 0.904 | 0.896 | 0.898 | 0.904 | 0.896 |  |  |  |
| Q13153 (ST) | 91 | 0.861 | 0.85 | 0.849 | 0.861 | 0.855 |  |  |  |
| Q13164 (ST) | 32 | 0.87 | 0.855 | 0.87 | 0.87 | 0.894 |  |  |  |
| Q13177 (ST) | 50 | 0.843 | 0.816 | 0.821 | 0.843 | 0.711 |  |  |  |
| Q13188 (ST) | 28 | 0.834 | 0.8 | 0.779 | 0.834 | 0.67 |  |  |  |
| Q13237 (ST) | 29 | 0.886 | 0.876 | 0.89 | 0.886 | 0.943 |  |  |  |
| Q13315 (ST) | 321 | 0.968 | 0.968 | 0.968 | 0.968 | 0.978 |  |  |  |
| Q13464 (ST) | 86 | 0.888 | 0.879 | 0.89 | 0.888 | 0.881 |  |  |  |
| Q13535 (ST) | 124 | 0.965 | 0.965 | 0.967 | 0.965 | 0.965 |  |  |  |
| Q13554 (ST) | 22 | 0.857 | 0.844 | 0.843 | 0.857 | 0.821 |  |  |  |
| Q13557 (ST) | 26 | 0.891 | 0.873 | 0.876 | 0.891 | 0.774 |  |  |  |
| Q13627 (ST) | 49 | 0.928 | 0.929 | 0.935 | 0.928 | 0.949 |  |  |  |
| Q13976 (ST) | 84 | 0.941 | 0.934 | 0.944 | 0.941 | 0.937 |  |  |  |
| Q14012 (ST) | 42 | 0.904 | 0.899 | 0.906 | 0.904 | 0.877 |  |  |  |
| Q14164 (ST) | 48 | 0.84 | 0.815 | 0.818 | 0.84 | 0.788 |  |  |  |
| Q14680 (ST) | 26 | 0.815 | 0.758 | 0.716 | 0.815 | 0.506 |  |  |  |
| Q15118 (ST) | 31 | 0.898 | 0.886 | 0.898 | 0.898 | 0.932 |  |  |  |
| Q15139 (ST) | 90 | 0.93 | 0.923 | 0.933 | 0.93 | 0.905 |  |  |  |
| Q15418 (ST) | 109 | 0.919 | 0.915 | 0.919 | 0.919 | 0.914 |  |  |  |
| Q15759 (ST) | 51 | 0.935 | 0.931 | 0.94 | 0.935 | 0.945 |  |  |  |
| Q15831 (ST) | 37 | 0.87 | 0.845 | 0.861 | 0.87 | 0.694 |  |  |  |
| Q15835 (ST) | 19 | 0.843 | 0.802 | 0.775 | 0.843 | 0.79 |  |  |  |
| Q16512 (ST) | 16 | 0.814 | 0.791 | 0.782 | 0.814 | 0.825 |  |  |  |
| Q16539 (ST) | 300 | 0.935 | 0.934 | 0.934 | 0.935 | 0.959 |  |  |  |
| Q16566 (ST) | 24 | 0.881 | 0.857 | 0.848 | 0.881 | 0.844 |  |  |  |
| Q2MHE4 (ST) | 38 | 0.624 | 0.599 | 0.62 | 0.624 | 0.772 |  |  |  |
| Q2TA25 (ST) | 16 | 0.791 | 0.735 | 0.694 | 0.791 | 0.425 |  |  |  |
| Q39011 (ST) | 36 | 0.568 | 0.539 | 0.544 | 0.568 | 0.534 |  |  |  |
| Q3SYZ2 (ST) | 16 | 0.762 | 0.722 | 0.689 | 0.762 | 0.606 |  |  |  |
| Q5EG47 (ST) | 36 | 0.912 | 0.906 | 0.91 | 0.912 | 0.912 |  |  |  |
| Q5RCH1 (ST) | 17 | 0.97 | 0.973 | 0.983 | 0.97 | 0.989 |  |  |  |
| Q5S007 (ST) | 91 | 0.846 | 0.809 | 0.808 | 0.846 | 0.696 |  |  |  |
| Q60670 (ST) | 15 | 0.9 | 0.875 | 0.862 | 0.9 | 0.907 |  |  |  |
| Q60680 (ST) | 18 | 0.835 | 0.8 | 0.777 | 0.835 | 0.672 |  |  |  |
| Q60737 (ST) | 68 | 0.88 | 0.871 | 0.876 | 0.88 | 0.902 |  |  |  |
| Q60806 (ST) | 16 | 0.803 | 0.744 | 0.696 | 0.803 | 0.506 |  |  |  |
| Q60823 (ST) | 21 | 0.976 | 0.974 | 0.98 | 0.976 | 0.976 |  |  |  |
| Q61036 (ST) | 17 | 0.885 | 0.849 | 0.824 | 0.885 | 0.642 |  |  |  |
| Q61831 (ST) | 20 | 0.917 | 0.913 | 0.916 | 0.917 | 0.96 |  |  |  |
| Q62101 (ST) | 19 | 0.938 | 0.928 | 0.937 | 0.938 | 0.838 |  |  |  |
| Q62388 (ST) | 49 | 0.959 | 0.96 | 0.963 | 0.959 | 0.944 |  |  |  |
| Q62868 (ST) | 20 | 0.85 | 0.799 | 0.764 | 0.85 | 0.535 |  |  |  |
| Q63450 (ST) | 18 | 0.899 | 0.879 | 0.871 | 0.899 | 0.822 |  |  |  |
| Q63470 (ST) | 19 | 0.859 | 0.836 | 0.82 | 0.859 | 0.834 |  |  |  |
| Q63531 (ST) | 27 | 0.913 | 0.903 | 0.917 | 0.913 | 0.908 |  |  |  |
| Q63644 (ST) | 17 | 0.882 | 0.856 | 0.84 | 0.882 | 0.815 |  |  |  |
| Q63699 (ST) | 20 | 0.942 | 0.942 | 0.953 | 0.942 | 0.925 |  |  |  |
| Q63844 (ST) | 106 | 0.958 | 0.957 | 0.958 | 0.958 | 0.952 |  |  |  |
| Q64303 (ST) | 15 | 0.811 | 0.78 | 0.764 | 0.811 | 0.491 |  |  |  |
| Q64702 (ST) | 16 | 0.874 | 0.84 | 0.817 | 0.874 | 0.881 |  |  |  |
| Q7KZI7 (ST) | 31 | 0.871 | 0.856 | 0.86 | 0.871 | 0.707 |  |  |  |
| Q8BSK8 (ST) | 29 | 0.919 | 0.911 | 0.924 | 0.919 | 0.859 |  |  |  |
| Q8C050 (ST) | 19 | 0.902 | 0.887 | 0.89 | 0.902 | 0.843 |  |  |  |
| Q8CIN4 (ST) | 17 | 0.823 | 0.794 | 0.776 | 0.823 | 0.636 |  |  |  |
| Q8IW41 (ST) | 18 | 0.909 | 0.885 | 0.885 | 0.909 | 0.828 |  |  |  |
| Q8IXL6 (ST) | 196 | 0.896 | 0.89 | 0.89 | 0.896 | 0.878 |  |  |  |
| Q8N5S9 (ST) | 15 | 0.9 | 0.872 | 0.861 | 0.9 | 0.938 |  |  |  |
| Q91Y86 (ST) | 76 | 0.958 | 0.959 | 0.962 | 0.958 | 0.961 |  |  |  |
| Q91YS8 (ST) | 16 | 0.909 | 0.883 | 0.869 | 0.909 | 0.85 |  |  |  |
| Q92630 (ST) | 37 | 0.91 | 0.908 | 0.914 | 0.91 | 0.91 |  |  |  |
| Q94F62 (ST) | 23 | 0.738 | 0.691 | 0.684 | 0.738 | 0.542 |  |  |  |
| Q96GD4 (ST) | 190 | 0.918 | 0.914 | 0.916 | 0.918 | 0.914 |  |  |  |
| Q96QT4 (ST) | 63 | 0.749 | 0.72 | 0.704 | 0.749 | 0.523 |  |  |  |
| Q96SB4 (ST) | 23 | 0.922 | 0.911 | 0.912 | 0.922 | 0.944 |  |  |  |
| Q99683 (ST) | 26 | 0.835 | 0.799 | 0.78 | 0.835 | 0.723 |  |  |  |
| Q99986 (ST) | 18 | 0.899 | 0.865 | 0.849 | 0.899 | 0.744 |  |  |  |
| Q9BXM7 (ST) | 17 | 0.862 | 0.818 | 0.792 | 0.862 | 0.588 |  |  |  |
| Q9BZL6 (ST) | 20 | 0.933 | 0.926 | 0.925 | 0.933 | 0.945 |  |  |  |
| Q9DC28 (ST) | 53 | 0.819 | 0.784 | 0.796 | 0.819 | 0.75 |  |  |  |
| Q9H0K1 (ST) | 17 | 0.912 | 0.893 | 0.886 | 0.912 | 0.835 |  |  |  |
| Q9H2X6 (ST) | 67 | 0.925 | 0.924 | 0.929 | 0.925 | 0.926 |  |  |  |
| Q9H4B4 (ST) | 34 | 0.83 | 0.793 | 0.765 | 0.83 | 0.79 |  |  |  |
| Q9HC98 (ST) | 22 | 0.894 | 0.88 | 0.886 | 0.894 | 0.915 |  |  |  |
| Q9JKK8 (ST) | 15 | 0.933 | 0.931 | 0.943 | 0.933 | 0.966 |  |  |  |
| Q9JLN9 (ST) | 104 | 0.907 | 0.901 | 0.902 | 0.907 | 0.897 |  |  |  |
| Q9NRM7 (ST) | 19 | 0.964 | 0.954 | 0.951 | 0.964 | 0.995 |  |  |  |
| Q9NWZ3 (ST) | 34 | 0.651 | 0.618 | 0.609 | 0.651 | 0.557 |  |  |  |
| Q9NYY3 (ST) | 33 | 0.818 | 0.761 | 0.715 | 0.818 | 0.654 |  |  |  |
| Q9P1W9 (ST) | 18 | 0.898 | 0.878 | 0.871 | 0.898 | 0.7 |  |  |  |
| Q9QZR5 (ST) | 22 | 0.88 | 0.873 | 0.892 | 0.88 | 0.93 |  |  |  |
| Q9R012 (ST) | 24 | 0.813 | 0.764 | 0.727 | 0.813 | 0.533 |  |  |  |
| Q9UBE8 (ST) | 16 | 0.866 | 0.847 | 0.845 | 0.866 | 0.938 |  |  |  |
| Q9UEW8 (ST) | 20 | 0.875 | 0.846 | 0.832 | 0.875 | 0.855 |  |  |  |
| Q9UHD2 (ST) | 88 | 0.856 | 0.821 | 0.832 | 0.856 | 0.737 |  |  |  |
| Q9UQM7 (ST) | 279 | 0.873 | 0.863 | 0.865 | 0.873 | 0.884 |  |  |  |
| Q9WTK7 (ST) | 17 | 0.921 | 0.907 | 0.903 | 0.921 | 0.762 |  |  |  |
| Q9WTU6 (ST) | 30 | 0.9 | 0.894 | 0.908 | 0.9 | 0.938 |  |  |  |
| Q9WV60 (ST) | 103 | 0.909 | 0.905 | 0.906 | 0.909 | 0.893 |  |  |  |
| Q9WVC6 (ST) | 32 | 0.948 | 0.942 | 0.954 | 0.948 | 0.981 |  |  |  |
| Q9Y478 (ST) | 57 | 0.918 | 0.905 | 0.906 | 0.918 | 0.891 |  |  |  |
| Q9Z2A0 (ST) | 35 | 0.929 | 0.912 | 0.905 | 0.929 | 0.951 |  |  |  |
| A9UF07 (Y) | 18 | 0.78 | 0.736 | 0.7 | 0.78 | 0.683 |  |  |  |
| O60674 (Y) | 59 | 0.815 | 0.773 | 0.773 | 0.815 | 0.706 |  |  |  |
| P00519 (Y) | 225 | 0.766 | 0.758 | 0.764 | 0.766 | 0.804 |  |  |  |
| P00520 (Y) | 82 | 0.802 | 0.779 | 0.784 | 0.802 | 0.735 |  |  |  |
| P00523 (Y) | 47 | 0.867 | 0.849 | 0.872 | 0.867 | 0.844 |  |  |  |
| P00533 (Y) | 116 | 0.796 | 0.772 | 0.782 | 0.796 | 0.736 |  |  |  |
| P04626 (Y) | 17 | 0.834 | 0.765 | 0.711 | 0.834 | 0.691 |  |  |  |
| P04629 (Y) | 15 | 0.78 | 0.75 | 0.767 | 0.78 | 0.733 |  |  |  |
| P05480 (Y) | 157 | 0.813 | 0.802 | 0.805 | 0.813 | 0.841 |  |  |  |
| P06213 (Y) | 71 | 0.806 | 0.77 | 0.772 | 0.806 | 0.763 |  |  |  |
| P06239 (Y) | 134 | 0.8 | 0.792 | 0.797 | 0.8 | 0.814 |  |  |  |
| P06240 (Y) | 34 | 0.881 | 0.859 | 0.873 | 0.881 | 0.861 |  |  |  |
| P06241 (Y) | 206 | 0.786 | 0.778 | 0.78 | 0.786 | 0.833 |  |  |  |
| P07947 (Y) | 25 | 0.773 | 0.736 | 0.707 | 0.773 | 0.593 |  |  |  |
| P07948 (Y) | 143 | 0.749 | 0.732 | 0.744 | 0.749 | 0.73 |  |  |  |
| P07949 (Y) | 27 | 0.814 | 0.772 | 0.745 | 0.814 | 0.682 |  |  |  |
| P08069 (Y) | 28 | 0.786 | 0.756 | 0.748 | 0.786 | 0.612 |  |  |  |
| P08103 (Y) | 15 | 0.8 | 0.751 | 0.71 | 0.8 | 0.709 |  |  |  |
| P08581 (Y) | 36 | 0.799 | 0.774 | 0.779 | 0.799 | 0.839 |  |  |  |
| P08631 (Y) | 36 | 0.81 | 0.796 | 0.811 | 0.81 | 0.713 |  |  |  |
| P09619 (Y) | 34 | 0.834 | 0.807 | 0.795 | 0.834 | 0.74 |  |  |  |
| P09769 (Y) | 19 | 0.8 | 0.754 | 0.718 | 0.8 | 0.724 |  |  |  |
| P11362 (Y) | 46 | 0.777 | 0.722 | 0.693 | 0.777 | 0.577 |  |  |  |
| P12931 (Y) | 652 | 0.762 | 0.761 | 0.765 | 0.762 | 0.838 |  |  |  |
| P15127 (Y) | 27 | 0.83 | 0.779 | 0.759 | 0.83 | 0.733 |  |  |  |
| P15208 (Y) | 26 | 0.842 | 0.821 | 0.827 | 0.842 | 0.793 |  |  |  |
| P16092 (Y) | 18 | 0.757 | 0.708 | 0.677 | 0.757 | 0.611 |  |  |  |
| P16591 (Y) | 21 | 0.801 | 0.759 | 0.729 | 0.801 | 0.553 |  |  |  |
| P23458 (Y) | 17 | 0.755 | 0.724 | 0.698 | 0.755 | 0.585 |  |  |  |
| P25911 (Y) | 51 | 0.831 | 0.815 | 0.827 | 0.831 | 0.856 |  |  |  |
| P29597 (Y) | 17 | 0.744 | 0.691 | 0.65 | 0.744 | 0.504 |  |  |  |
| P32577 (Y) | 22 | 0.823 | 0.769 | 0.736 | 0.823 | 0.72 |  |  |  |
| P39688 (Y) | 65 | 0.815 | 0.788 | 0.783 | 0.815 | 0.764 |  |  |  |
| P41240 (Y) | 28 | 0.85 | 0.827 | 0.839 | 0.85 | 0.662 |  |  |  |
| P41241 (Y) | 22 | 0.816 | 0.776 | 0.751 | 0.816 | 0.635 |  |  |  |
| P42684 (Y) | 33 | 0.851 | 0.835 | 0.831 | 0.851 | 0.739 |  |  |  |
| P43403 (Y) | 39 | 0.77 | 0.744 | 0.767 | 0.77 | 0.715 |  |  |  |
| P43405 (Y) | 82 | 0.858 | 0.848 | 0.859 | 0.858 | 0.882 |  |  |  |
| P48025 (Y) | 36 | 0.846 | 0.814 | 0.809 | 0.846 | 0.841 |  |  |  |
| P51813 (Y) | 22 | 0.857 | 0.814 | 0.791 | 0.857 | 0.752 |  |  |  |
| P52333 (Y) | 23 | 0.805 | 0.76 | 0.724 | 0.805 | 0.558 |  |  |  |
| Q01279 (Y) | 26 | 0.834 | 0.784 | 0.745 | 0.834 | 0.719 |  |  |  |
| Q05397 (Y) | 29 | 0.868 | 0.828 | 0.814 | 0.868 | 0.765 |  |  |  |
| Q06187 (Y) | 22 | 0.847 | 0.805 | 0.78 | 0.847 | 0.63 |  |  |  |
| Q07014 (Y) | 18 | 0.788 | 0.746 | 0.714 | 0.788 | 0.706 |  |  |  |
| Q08881 (Y) | 15 | 0.8 | 0.741 | 0.693 | 0.8 | 0.58 |  |  |  |
| Q13882 (Y) | 26 | 0.808 | 0.767 | 0.739 | 0.808 | 0.523 |  |  |  |
| Q14289 (Y) | 18 | 0.825 | 0.772 | 0.732 | 0.825 | 0.622 |  |  |  |
| Q15046 (Y) | 38 | 0.908 | 0.895 | 0.895 | 0.908 | 0.938 |  |  |  |
| Q16620 (Y) | 17 | 0.789 | 0.726 | 0.675 | 0.789 | 0.821 |  |  |  |
| Q62120 (Y) | 48 | 0.716 | 0.676 | 0.654 | 0.716 | 0.66 |  |  |  |
| Q62689 (Y) | 22 | 0.773 | 0.731 | 0.713 | 0.773 | 0.618 |  |  |  |
| Q62844 (Y) | 22 | 0.849 | 0.812 | 0.797 | 0.849 | 0.677 |  |  |  |
| Q9WUD9 (Y) | 75 | 0.818 | 0.782 | 0.782 | 0.818 | 0.721 |  |  |  |

*Note*: SHAP, the SHapley Additive exPlanations.
