# Supplementary material for: KinasePhos 3.0: Redesign and Expansion of the Prediction on Kinase-specific Phosphorylation Sites
Source: Genomics Proteomics Bioinformatics. 2022 Jul 1;21(1):228–41. doi: 10.1016/j.gpb.2022.06.004 (PMC10373160; doi:10.1016/j.gpb.2022.06.004)
Supplement: Supplementary Figure S1 — The evolutionary tree for all the collected kinases The obtained evolutionary tree showed that homologous proteins or proteins with consistent domains clustered tightly in smaller branches. [file mmc1.pdf]

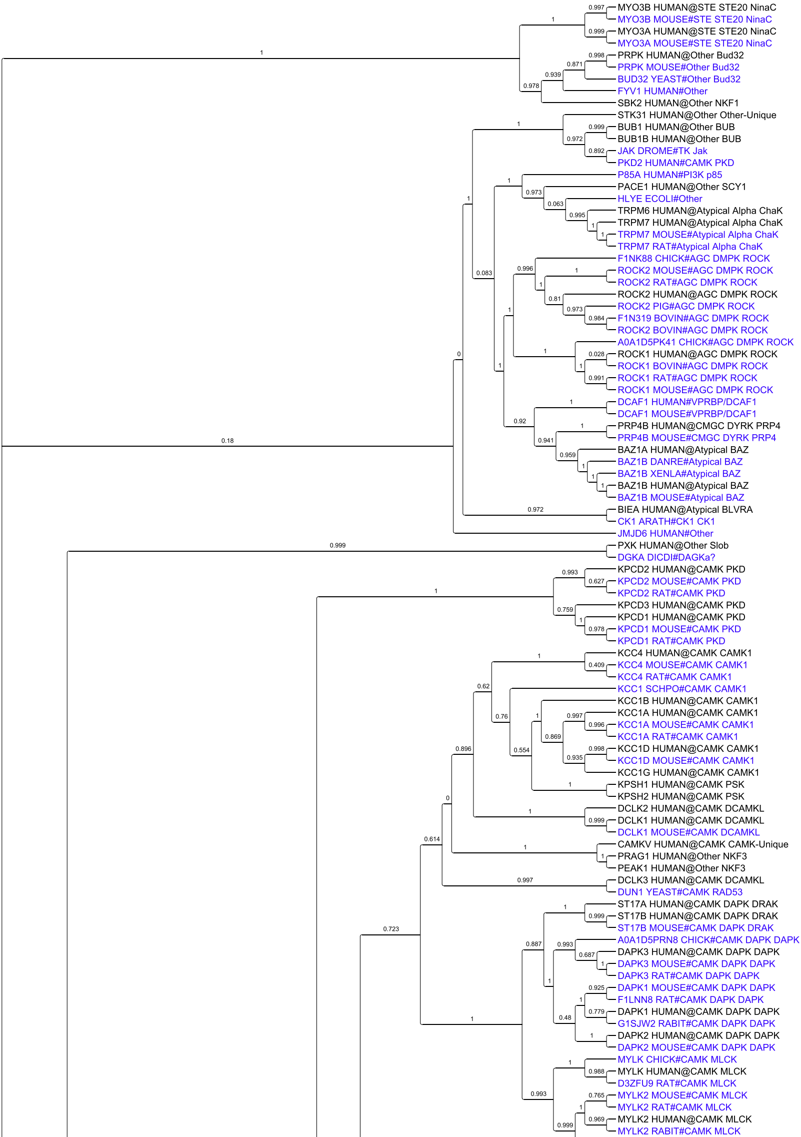
MYO3B HUMAN@STE STE20 NinaC
MYO3B MOUSE#STE STE20 NinaC
MYO3A HUMAN@STE STE20 NinaC
MYO3A MOUSE#STE STE20 NinaC
PRPK HUMAN@Other Bud32
PRPK MOUSE#Other Bud32
BUD32 YEAST#Other Bud32
FYV1 HUMAN#Other
SBK2 HUMAN@Other NKF1
STK31 HUMAN@Other Other-Unique
BUB1 HUMAN@Other BUB
BUB1B HUMAN@Other BUB
JAK DROME#TK Jak
PKD2 HUMAN#CAMK PKD
P85A HUMAN#PI3K p85
PACE1 HUMAN@Other SCY1
HLYE ECOLI#Other
TRPM6 HUMAN@Atypical Alpha ChaK
TRPM7 HUMAN@Atypical Alpha ChaK
TRPM7 MOUSE#Atypical Alpha ChaK
TRPM7 RAT#Atypical Alpha ChaK
F1NK88 CHICK#AGC DMPK ROCK
ROCK2 MOUSE#AGC DMPK ROCK
ROCK2 RAT#AGC DMPK ROCK
ROCK2 HUMAN@AGC DMPK ROCK
ROCK2 PIG#AGC DMPK ROCK
F1N319 BOVIN#AGC DMPK ROCK
ROCK2 BOVIN#AGC DMPK ROCK
A0A1D5PK41 CHICK#AGC DMPK ROCK
ROCK1 HUMAN@AGC DMPK ROCK
ROCK1 BOVIN#AGC DMPK ROCK
ROCK1 RAT#AGC DMPK ROCK
ROCK1 MOUSE#AGC DMPK ROCK
DCAF1 HUMAN#VPRBP/DCAF1
DCAF1 MOUSE#VPRBP/DCAF1
PRP4B HUMAN@CMGC DYRK PRP4
PRP4B MOUSE#CMGC DYRK PRP4
BAZ1A HUMAN@Atypical BAZ
BAZ1B DANRE#Atypical BAZ
BAZ1B XENLA#Atypical BAZ
BAZ1B HUMAN@Atypical BAZ
BAZ1B MOUSE#Atypical BAZ
BIEA HUMAN@Atypical BLVRA
CK1 ARATH#CK1 CK1
JMJD6 HUMAN#Other
PXK HUMAN@Other Slob
DGKA DICDI#DAGKa?
KPCD2 HUMAN@CAMK PKD
KPCD2 MOUSE#CAMK PKD
KPCD2 RAT#CAMK PKD
KPCD3 HUMAN@CAMK PKD
KPCD1 HUMAN@CAMK PKD
KPCD1 MOUSE#CAMK PKD
KPCD1 RAT#CAMK PKD
KCC4 HUMAN@CAMK CAMK1
KCC4 MOUSE#CAMK CAMK1
KCC4 RAT#CAMK CAMK1
KCC1 SCHPO#CAMK CAMK1
KCC1B HUMAN@CAMK CAMK1
KCC1A HUMAN@CAMK CAMK1
KCC1A MOUSE#CAMK CAMK1
KCC1A RAT#CAMK CAMK1
KCC1D HUMAN@CAMK CAMK1
KCC1D MOUSE#CAMK CAMK1
KCC1G HUMAN@CAMK CAMK1
KPSH1 HUMAN@CAMK PSK
KPSH2 HUMAN@CAMK PSK
DCLK2 HUMAN@CAMK DCAMKL
DCLK1 HUMAN@CAMK DCAMKL
DCLK1 MOUSE#CAMK DCAMKL
CAMKV HUMAN@CAMK CAMK-Unique
PRAG1 HUMAN@Other NKF3
PEAK1 HUMAN@Other NKF3
DCLK3 HUMAN@CAMK DCAMKL
DUN1 YEAST#CAMK RAD53
ST17A HUMAN@CAMK DAPK DRAK
ST17B HUMAN@CAMK DAPK DRAK
ST17B MOUSE#CAMK DAPK DRAK
A0A1D5PRN8 CHICK#CAMK DAPK DAPK
DAPK3 HUMAN@CAMK DAPK DAPK
DAPK3 MOUSE#CAMK DAPK DAPK
DAPK3 RAT#CAMK DAPK DAPK
DAPK1 MOUSE#CAMK DAPK DAPK
F1LNN8 RAT#CAMK DAPK DAPK
DAPK1 HUMAN@CAMK DAPK DAPK
G1SJW2 RABIT#CAMK DAPK DAPK
DAPK2 HUMAN@CAMK DAPK DAPK
DAPK2 MOUSE#CAMK DAPK DAPK
MYLK CHICK#CAMK MLCK
MYLK HUMAN@CAMK MLCK
D3ZFU9 RAT#CAMK MLCK
MYLK2 MOUSE#CAMK MLCK
MYLK2 RAT#CAMK MLCK
MYLK2 HUMAN@CAMK MLCK
MYLK2 RABIT#CAMK MLCK

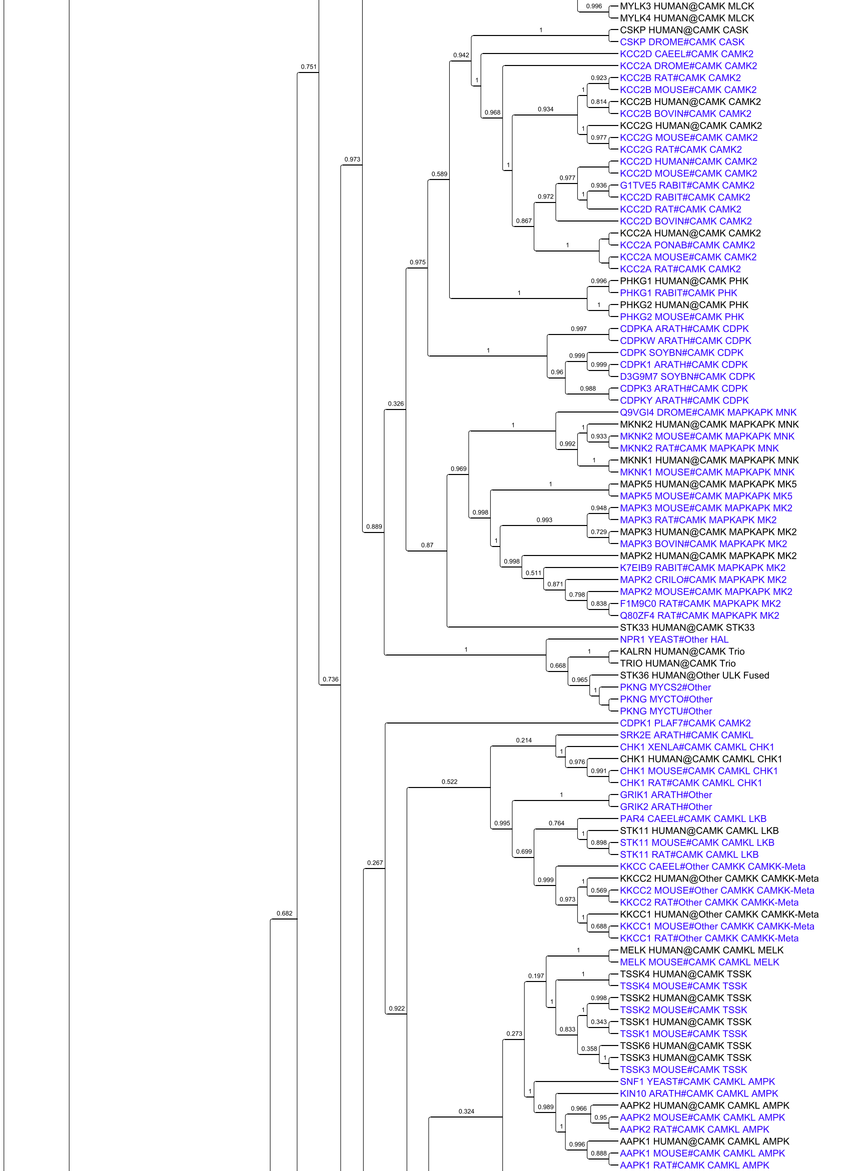
MYLK3 HUMAN@CAMK MLCK
MYLK4 HUMAN@CAMK MLCK
CSKP HUMAN@CAMK CASK
CSKP DROME#CAMK CASK
KCC2D CAEEL#CAMK CAMK2
KCC2A DROME#CAMK CAMK2
KCC2B RAT#CAMK CAMK2
KCC2B MOUSE#CAMK CAMK2
KCC2B HUMAN@CAMK CAMK2
KCC2B BOVIN#CAMK CAMK2
KCC2G HUMAN@CAMK CAMK2
KCC2G MOUSE#CAMK CAMK2
KCC2G RAT#CAMK CAMK2
KCC2D HUMAN#CAMK CAMK2
KCC2D MOUSE#CAMK CAMK2
G1TVE5 RABIT#CAMK CAMK2
KCC2D RABIT#CAMK CAMK2
KCC2D RAT#CAMK CAMK2
KCC2D BOVIN#CAMK CAMK2
KCC2A HUMAN@CAMK CAMK2
KCC2A PONAB#CAMK CAMK2
KCC2A MOUSE#CAMK CAMK2
KCC2A RAT#CAMK CAMK2
PHKG1 HUMAN@CAMK PHK
PHKG1 RABIT#CAMK PHK
PHKG2 HUMAN@CAMK PHK
PHKG2 MOUSE#CAMK PHK
CDPKA ARATH#CAMK CDPK
CDPKW ARATH#CAMK CDPK
CDPK SOYBN#CAMK CDPK
CDPK1 ARATH#CAMK CDPK
D3G9M7 SOYBN#CAMK CDPK
CDPK3 ARATH#CAMK CDPK
CDPKY ARATH#CAMK CDPK
Q9VGI4 DROME#CAMK MAPKAPK MNK
MKNK2 HUMAN@CAMK MAPKAPK MNK
MKNK2 MOUSE#CAMK MAPKAPK MNK
MKNK2 RAT#CAMK MAPKAPK MNK
MKNK1 HUMAN@CAMK MAPKAPK MNK
MKNK1 MOUSE#CAMK MAPKAPK MNK
MAPK5 HUMAN@CAMK MAPKAPK MK5
MAPK5 MOUSE#CAMK MAPKAPK MK5
MAPK3 MOUSE#CAMK MAPKAPK MK2
MAPK3 RAT#CAMK MAPKAPK MK2
MAPK3 HUMAN@CAMK MAPKAPK MK2
MAPK3 BOVIN#CAMK MAPKAPK MK2
MAPK2 HUMAN@CAMK MAPKAPK MK2
K7EIB9 RABIT#CAMK MAPKAPK MK2
MAPK2 CRILO#CAMK MAPKAPK MK2
MAPK2 MOUSE#CAMK MAPKAPK MK2
F1M9C0 RAT#CAMK MAPKAPK MK2
Q80ZF4 RAT#CAMK MAPKAPK MK2
STK33 HUMAN@CAMK STK33
NPR1 YEAST#Other HAL
KALRN HUMAN@CAMK Trio
TRIO HUMAN@CAMK Trio
STK36 HUMAN@Other ULK Fused
PKNG MYCS2#Other
PKNG MYCTO#Other
PKNG MYCTU#Other
CDPK1 PLAF7#CAMK CAMK2
SRK2E ARATH#CAMK CAMKL
CHK1 XENLA#CAMK CAMKL CHK1
CHK1 HUMAN@CAMK CAMKL CHK1
CHK1 MOUSE#CAMK CAMKL CHK1
CHK1 RAT#CAMK CAMKL CHK1
GRIK1 ARATH#Other
GRIK2 ARATH#Other
PAR4 CAEEL#CAMK CAMKL LKB
STK11 HUMAN@CAMK CAMKL LKB
STK11 MOUSE#CAMK CAMKL LKB
STK11 RAT#CAMK CAMKL LKB
KKCC CAEEL#Other CAMKK CAMKK-Meta
KKCC2 HUMAN@Other CAMKK CAMKK-Meta
KKCC2 MOUSE#Other CAMKK CAMKK-Meta
KKCC2 RAT#Other CAMKK CAMKK-Meta
KKCC1 HUMAN@Other CAMKK CAMKK-Meta
KKCC1 MOUSE#Other CAMKK CAMKK-Meta
KKCC1 RAT#Other CAMKK CAMKK-Meta
MELK HUMAN@CAMK CAMKL MELK
MELK MOUSE#CAMK CAMKL MELK
TSSK4 HUMAN@CAMK TSSK
TSSK4 MOUSE#CAMK TSSK
TSSK2 HUMAN@CAMK TSSK
TSSK2 MOUSE#CAMK TSSK
TSSK1 HUMAN@CAMK TSSK
TSSK1 MOUSE#CAMK TSSK
TSSK6 HUMAN@CAMK TSSK
TSSK3 HUMAN@CAMK TSSK
TSSK3 MOUSE#CAMK TSSK
SNF1 YEAST#CAMK CAMKL AMPK
KIN10 ARATH#CAMK CAMKL AMPK
AAPK2 HUMAN@CAMK CAMKL AMPK
AAPK2 MOUSE#CAMK CAMKL AMPK
AAPK2 RAT#CAMK CAMKL AMPK
AAPK1 HUMAN@CAMK CAMKL AMPK
AAPK1 MOUSE#CAMK CAMKL AMPK
AAPK1 RAT#CAMK CAMKL AMPK

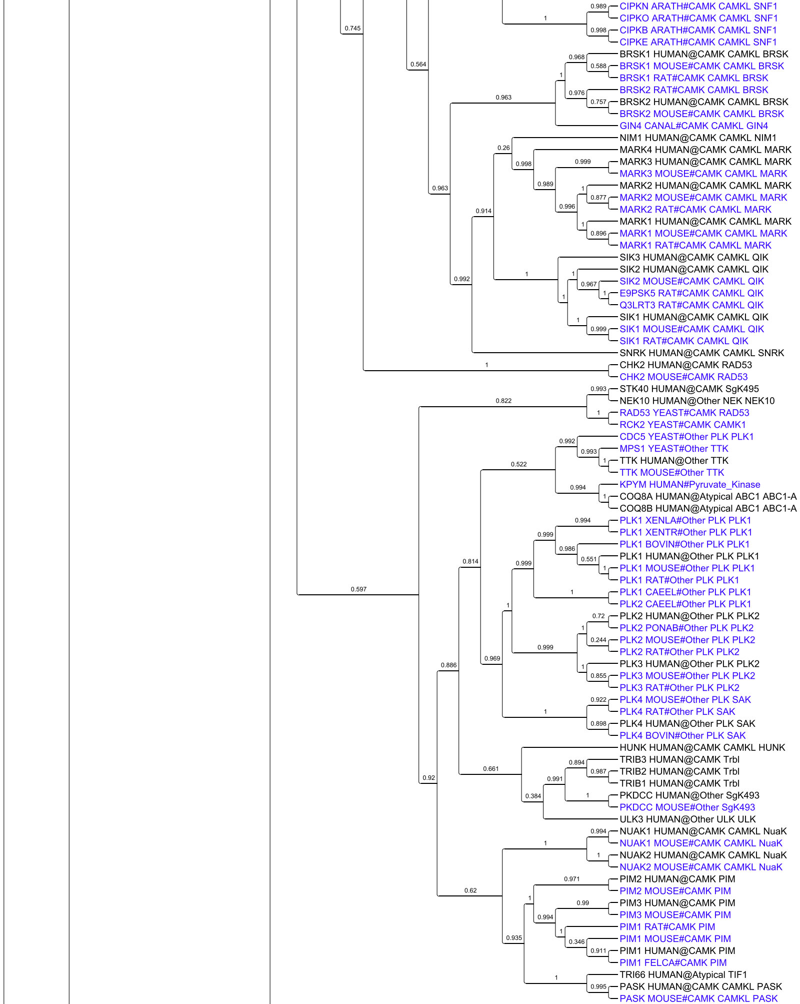
CIPKN ARATH#CAMK CAMKL SNF1
CIPKO ARATH#CAMK CAMKL SNF1
CIPKB ARATH#CAMK CAMKL SNF1
CIPKE ARATH#CAMK CAMKL SNF1
BRSK1 HUMAN@CAMK CAMKL BRSK
BRSK1 MOUSE#CAMK CAMKL BRSK
BRSK1 RAT#CAMK CAMKL BRSK
BRSK2 RAT#CAMK CAMKL BRSK
BRSK2 HUMAN@CAMK CAMKL BRSK
BRSK2 MOUSE#CAMK CAMKL BRSK
GIN4 CANAL#CAMK CAMKL GIN4
NIM1 HUMAN@CAMK CAMKL NIM1
MARK4 HUMAN@CAMK CAMKL MARK
MARK3 HUMAN@CAMK CAMKL MARK
MARK3 MOUSE#CAMK CAMKL MARK
MARK2 HUMAN@CAMK CAMKL MARK
MARK2 MOUSE#CAMK CAMKL MARK
MARK2 RAT#CAMK CAMKL MARK
MARK1 HUMAN@CAMK CAMKL MARK
MARK1 MOUSE#CAMK CAMKL MARK
MARK1 RAT#CAMK CAMKL MARK
SIK3 HUMAN@CAMK CAMKL QIK
SIK2 HUMAN@CAMK CAMKL QIK
SIK2 MOUSE#CAMK CAMKL QIK
E9PSK5 RAT#CAMK CAMKL QIK
Q3LRT3 RAT#CAMK CAMKL QIK
SIK1 HUMAN@CAMK CAMKL QIK
SIK1 MOUSE#CAMK CAMKL QIK
SIK1 RAT#CAMK CAMKL QIK
SNRK HUMAN@CAMK CAMKL SNRK
CHK2 HUMAN@CAMK RAD53
CHK2 MOUSE#CAMK RAD53
STK40 HUMAN@CAMK SgK495
NEK10 HUMAN@Other NEK NEK10
RAD53 YEAST#CAMK RAD53
RCK2 YEAST#CAMK CAMK1
CDC5 YEAST#Other PLK PLK1
MPS1 YEAST#Other TTK
TTK HUMAN@Other TTK
TTK MOUSE#Other TTK
KPYM HUMAN#Pyruvate_Kinase
COQ8A HUMAN@Atypical ABC1 ABC1-A
COQ8B HUMAN@Atypical ABC1 ABC1-A
PLK1 XENLA#Other PLK PLK1
PLK1 XENTR#Other PLK PLK1
PLK1 BOVIN#Other PLK PLK1
PLK1 HUMAN@Other PLK PLK1
PLK1 MOUSE#Other PLK PLK1
PLK1 RAT#Other PLK PLK1
PLK1 CAEEL#Other PLK PLK1
PLK2 CAEEL#Other PLK PLK1
PLK2 HUMAN@Other PLK PLK2
PLK2 PONAB#Other PLK PLK2
PLK2 MOUSE#Other PLK PLK2
PLK2 RAT#Other PLK PLK2
PLK3 HUMAN@Other PLK PLK2
PLK3 MOUSE#Other PLK PLK2
PLK3 RAT#Other PLK PLK2
PLK4 MOUSE#Other PLK SAK
PLK4 RAT#Other PLK SAK
PLK4 HUMAN@Other PLK SAK
PLK4 BOVIN#Other PLK SAK
HUNK HUMAN@CAMK CAMKL HUNK
TRIB3 HUMAN@CAMK Trbl
TRIB2 HUMAN@CAMK Trbl
TRIB1 HUMAN@CAMK Trbl
PKDCC HUMAN@Other SgK493
PKDCC MOUSE#Other SgK493
ULK3 HUMAN@Other ULK ULK
NUAK1 HUMAN@CAMK CAMKL NuaK
NUAK1 MOUSE#CAMK CAMKL NuaK
NUAK2 HUMAN@CAMK CAMKL NuaK
NUAK2 MOUSE#CAMK CAMKL NuaK
PIM2 HUMAN@CAMK PIM
PIM2 MOUSE#CAMK PIM
PIM3 HUMAN@CAMK PIM
PIM3 MOUSE#CAMK PIM
PIM1 RAT#CAMK PIM
PIM1 MOUSE#CAMK PIM
PIM1 HUMAN@CAMK PIM
PIM1 FELCA#CAMK PIM
TRI66 HUMAN@Atypical TIF1
PASK HUMAN@CAMK CAMKL PASK
PASK MOUSE#CAMK CAMKL PASK

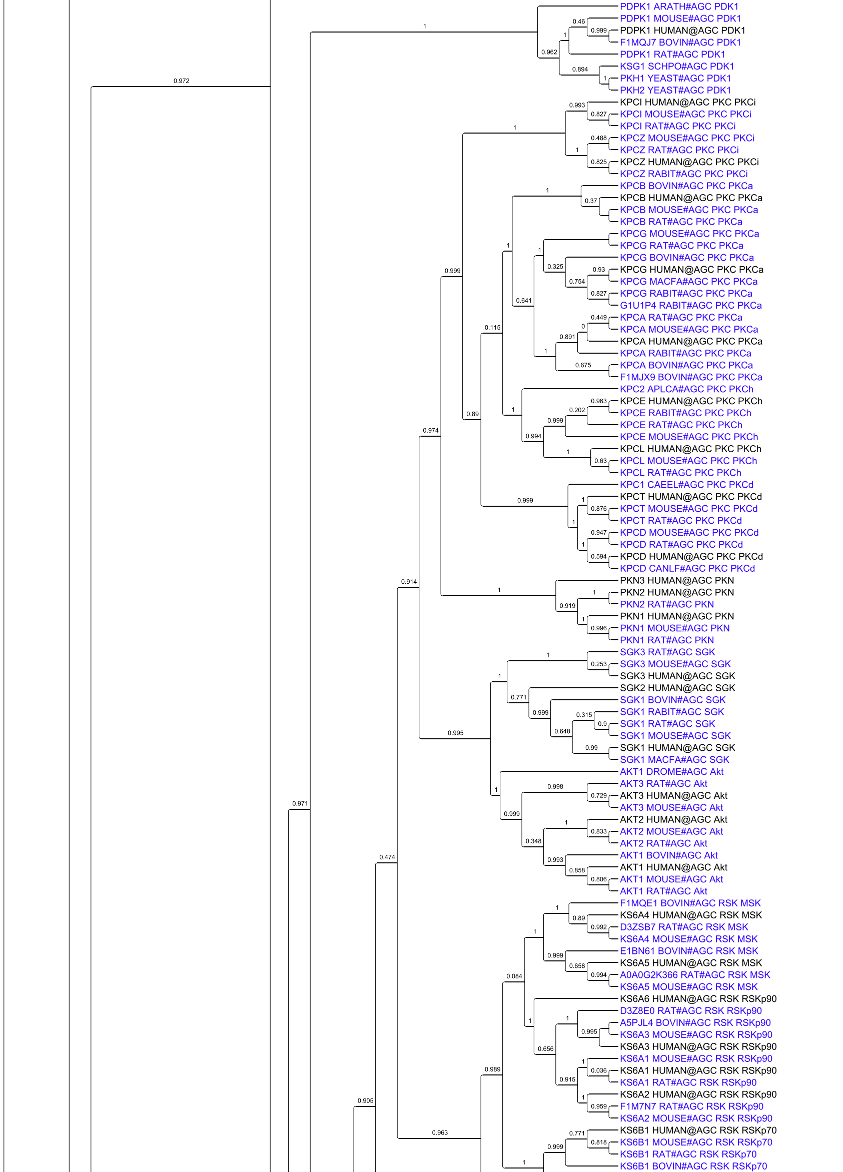

PDPK1 ARATH#AGC PDK1
PDPK1 MOUSE#AGC PDK1
PDPK1 HUMAN@AGC PDK1
F1MQJ7 BOVIN#AGC PDK1
PDPK1 RAT#AGC PDK1
KSG1 SCHPO#AGC PDK1
PKH1 YEAST#AGC PDK1
PKH2 YEAST#AGC PDK1
KPCI HUMAN@AGC PKC PKCi
KPCI MOUSE#AGC PKC PKCi
KPCI RAT#AGC PKC PKCi
KPCZ MOUSE#AGC PKC PKCi
KPCZ RAT#AGC PKC PKCi
KPCZ HUMAN@AGC PKC PKCi
KPCZ RABIT#AGC PKC PKCi
KPCB BOVIN#AGC PKC PKCa
KPCB HUMAN@AGC PKC PKCa
KPCB MOUSE#AGC PKC PKCa
KPCB RAT#AGC PKC PKCa
KPCG MOUSE#AGC PKC PKCa
KPCG RAT#AGC PKC PKCa
KPCG BOVIN#AGC PKC PKCa
KPCG HUMAN@AGC PKC PKCa
KPCG MACFA#AGC PKC PKCa
KPCG RABIT#AGC PKC PKCa
G1U1P4 RABIT#AGC PKC PKCa
KPCA RAT#AGC PKC PKCa
KPCA MOUSE#AGC PKC PKCa
KPCA HUMAN@AGC PKC PKCa
KPCA RABIT#AGC PKC PKCa
KPCA BOVIN#AGC PKC PKCa
F1MJX9 BOVIN#AGC PKC PKCa
KPC2 APLCA#AGC PKC PKCh
KPCE HUMAN@AGC PKC PKCh
KPCE RABIT#AGC PKC PKCh
KPCE RAT#AGC PKC PKCh
KPCE MOUSE#AGC PKC PKCh
KPCL HUMAN@AGC PKC PKCh
KPCL MOUSE#AGC PKC PKCh
KPCL RAT#AGC PKC PKCh
KPC1 CAEEL#AGC PKC PKCd
KPCT HUMAN@AGC PKC PKCd
KPCT MOUSE#AGC PKC PKCd
KPCT RAT#AGC PKC PKCd
KPCD MOUSE#AGC PKC PKCd
KPCD RAT#AGC PKC PKCd
KPCD HUMAN@AGC PKC PKCd
KPCD CANLF#AGC PKC PKCd
PKN3 HUMAN@AGC PKN
PKN2 HUMAN@AGC PKN
PKN2 RAT#AGC PKN
PKN1 HUMAN@AGC PKN
PKN1 MOUSE#AGC PKN
PKN1 RAT#AGC PKN
SGK3 RAT#AGC SGK
SGK3 MOUSE#AGC SGK
SGK3 HUMAN@AGC SGK
SGK2 HUMAN@AGC SGK
SGK1 BOVIN#AGC SGK
SGK1 RABIT#AGC SGK
SGK1 RAT#AGC SGK
SGK1 MOUSE#AGC SGK
SGK1 HUMAN@AGC SGK
SGK1 MACFA#AGC SGK
AKT1 DROME#AGC Akt
AKT3 RAT#AGC Akt
AKT3 HUMAN@AGC Akt
AKT3 MOUSE#AGC Akt
AKT2 HUMAN@AGC Akt
AKT2 MOUSE#AGC Akt
AKT2 RAT#AGC Akt
AKT1 BOVIN#AGC Akt
AKT1 HUMAN@AGC Akt
AKT1 MOUSE#AGC Akt
AKT1 RAT#AGC Akt
F1MQE1 BOVIN#AGC RSK MSK
KS6A4 HUMAN@AGC RSK MSK
D3ZSB7 RAT#AGC RSK MSK
KS6A4 MOUSE#AGC RSK MSK
E1BN61 BOVIN#AGC RSK MSK
KS6A5 HUMAN@AGC RSK MSK
A0A0G2K366 RAT#AGC RSK MSK
KS6A5 MOUSE#AGC RSK MSK
KS6A6 HUMAN@AGC RSK RSKp90
D3Z8E0 RAT#AGC RSK RSKp90
A5PJL4 BOVIN#AGC RSK RSKp90
KS6A3 MOUSE#AGC RSK RSKp90
KS6A3 HUMAN@AGC RSK RSKp90
KS6A1 MOUSE#AGC RSK RSKp90
KS6A1 HUMAN@AGC RSK RSKp90
KS6A1 RAT#AGC RSK RSKp90
KS6A2 HUMAN@AGC RSK RSKp90
F1M7N7 RAT#AGC RSK RSKp90
KS6A2 MOUSE#AGC RSK RSKp90
KS6B1 HUMAN@AGC RSK RSKp70
KS6B1 MOUSE#AGC RSK RSKp70
KS6B1 RAT#AGC RSK RSKp70
KS6B1 BOVIN#AGC RSK RSKp70

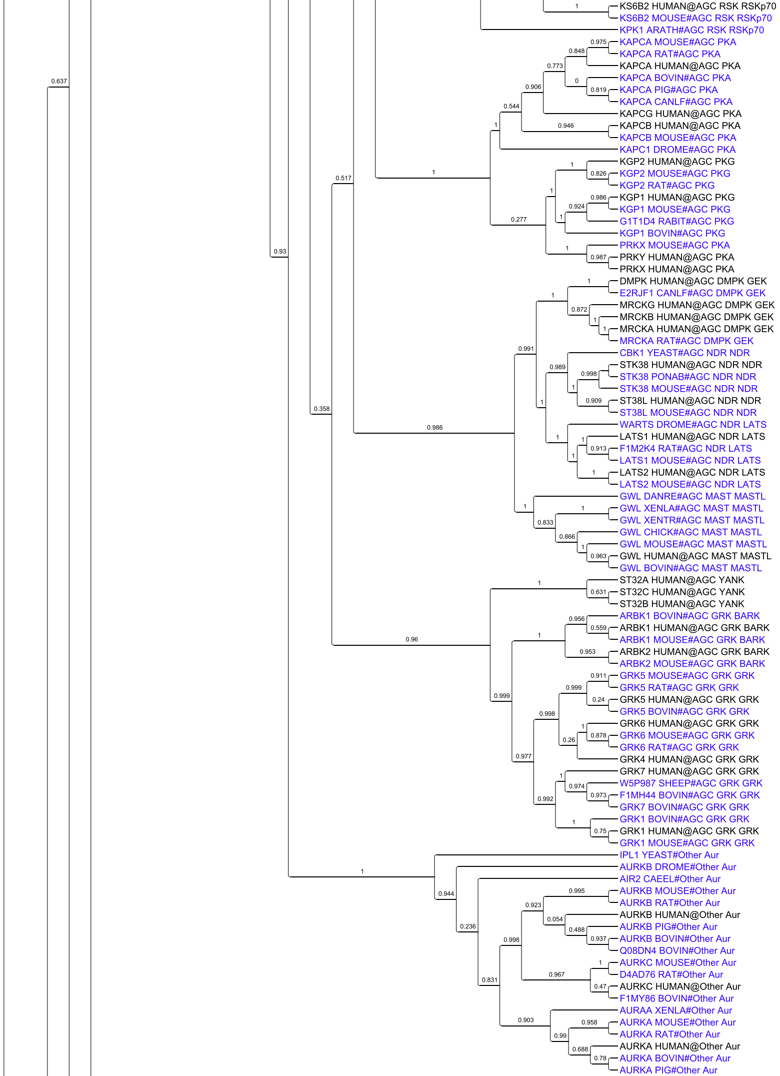
KS6B2 HUMAN@AGC RSK RSKp70
KS6B2 MOUSE#AGC RSK RSKp70
KPK1 ARATH#AGC RSK RSKp70
KAPCA MOUSE#AGC PKA
KAPCA RAT#AGC PKA
KAPCA HUMAN@AGC PKA
KAPCA BOVIN#AGC PKA
KAPCA PIG#AGC PKA
KAPCA CANLF#AGC PKA
KAPCG HUMAN@AGC PKA
KAPCB HUMAN@AGC PKA
KAPCB MOUSE#AGC PKA
KAPC1 DROME#AGC PKA
KGP2 HUMAN@AGC PKG
KGP2 MOUSE#AGC PKG
KGP2 RAT#AGC PKG
KGP1 HUMAN@AGC PKG
KGP1 MOUSE#AGC PKG
G1T1D4 RABIT#AGC PKG
KGP1 BOVIN#AGC PKG
PRKX MOUSE#AGC PKA
PRKY HUMAN@AGC PKA
PRKX HUMAN@AGC PKA
DMPK HUMAN@AGC DMPK GEK
E2RJF1 CANLF#AGC DMPK GEK
MRCKG HUMAN@AGC DMPK GEK
MRCKB HUMAN@AGC DMPK GEK
MRCKA HUMAN@AGC DMPK GEK
MRCKA RAT#AGC DMPK GEK
CBK1 YEAST#AGC NDR NDR
STK38 HUMAN@AGC NDR NDR
STK38 PONAB#AGC NDR NDR
STK38 MOUSE#AGC NDR NDR
ST38L HUMAN@AGC NDR NDR
ST38L MOUSE#AGC NDR NDR
WARTS DROME#AGC NDR LATS
LATS1 HUMAN@AGC NDR LATS
F1M2K4 RAT#AGC NDR LATS
LATS1 MOUSE#AGC NDR LATS
LATS2 HUMAN@AGC NDR LATS
LATS2 MOUSE#AGC NDR LATS
GWL DANRE#AGC MAST MASTL
GWL XENLA#AGC MAST MASTL
GWL XENTR#AGC MAST MASTL
GWL CHICK#AGC MAST MASTL
GWL MOUSE#AGC MAST MASTL
GWL HUMAN@AGC MAST MASTL
GWL BOVIN#AGC MAST MASTL
ST32A HUMAN@AGC YANK
ST32C HUMAN@AGC YANK
ST32B HUMAN@AGC YANK
ARBK1 BOVIN#AGC GRK BARK
ARBK1 HUMAN@AGC GRK BARK
ARBK1 MOUSE#AGC GRK BARK
ARBK2 HUMAN@AGC GRK BARK
ARBK2 MOUSE#AGC GRK BARK
GRK5 MOUSE#AGC GRK GRK
GRK5 RAT#AGC GRK GRK
GRK5 HUMAN@AGC GRK GRK
GRK5 BOVIN#AGC GRK GRK
GRK6 HUMAN@AGC GRK GRK
GRK6 MOUSE#AGC GRK GRK
GRK6 RAT#AGC GRK GRK
GRK4 HUMAN@AGC GRK GRK
GRK7 HUMAN@AGC GRK GRK
W5P987 SHEEP#AGC GRK GRK
F1MH44 BOVIN#AGC GRK GRK
GRK7 BOVIN#AGC GRK GRK
GRK1 BOVIN#AGC GRK GRK
GRK1 HUMAN@AGC GRK GRK
GRK1 MOUSE#AGC GRK GRK
IPL1 YEAST#Other Aur
AURKB DROME#Other Aur
AIR2 CAEEL#Other Aur
AURKB MOUSE#Other Aur
AURKB RAT#Other Aur
AURKB HUMAN@Other Aur
AURKB PIG#Other Aur
AURKB BOVIN#Other Aur
Q08DN4 BOVIN#Other Aur
AURKC MOUSE#Other Aur
D4AD76 RAT#Other Aur
AURKC HUMAN@Other Aur
F1MY86 BOVIN#Other Aur
AURAA XENLA#Other Aur
AURKA MOUSE#Other Aur
AURKA RAT#Other Aur
AURKA HUMAN@Other Aur
AURKA BOVIN#Other Aur
AURKA PIG#Other Aur

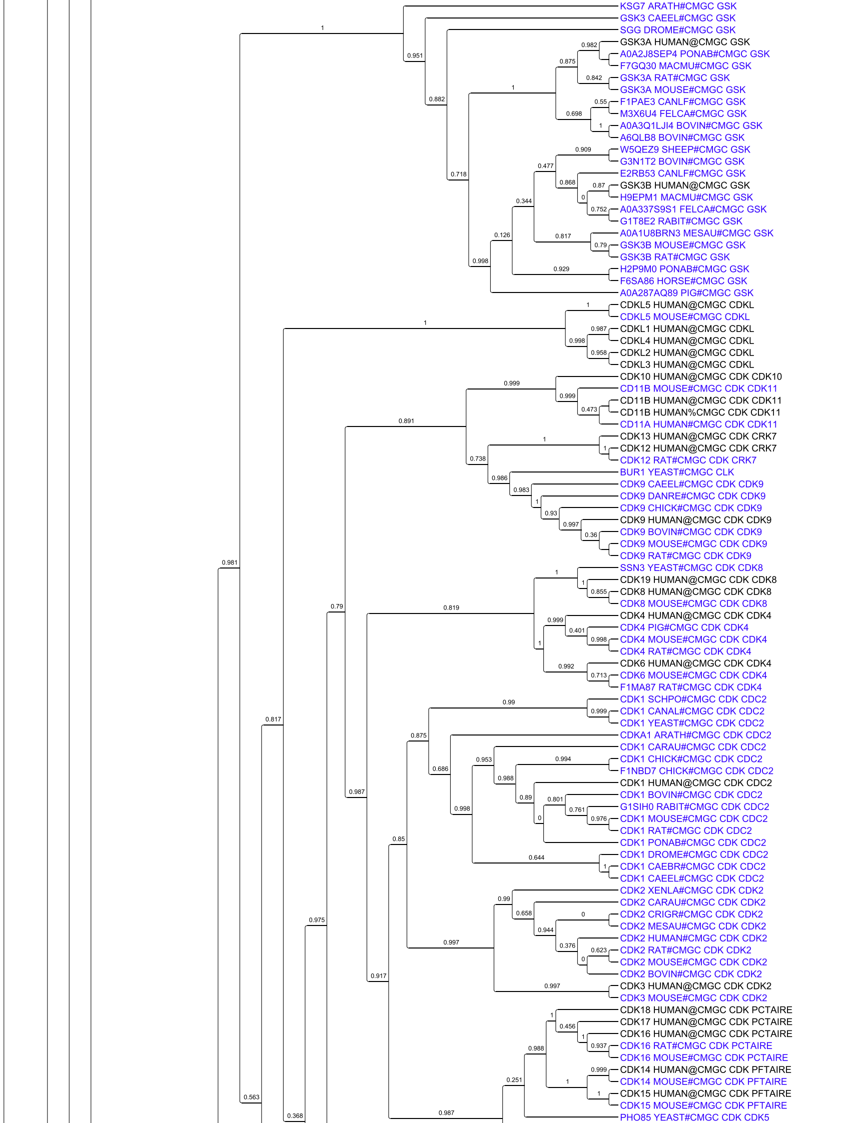
KSG7 ARATH#CMGC GSK
GSK3 CAEEL#CMGC GSK
SGG DROME#CMGC GSK
GSK3A HUMAN@CMGC GSK
A0A2J8SEP4 PONAB#CMGC GSK
F7GQ30 MACMU#CMGC GSK
GSK3A RAT#CMGC GSK
GSK3A MOUSE#CMGC GSK
F1PAE3 CANLF#CMGC GSK
M3X6U4 FELCA#CMGC GSK
A0A3Q1LJI4 BOVIN#CMGC GSK
A6QLB8 BOVIN#CMGC GSK
W5QEZ9 SHEEP#CMGC GSK
G3N1T2 BOVIN#CMGC GSK
E2RB53 CANLF#CMGC GSK
GSK3B HUMAN@CMGC GSK
H9EPM1 MACMU#CMGC GSK
A0A337S9S1 FELCA#CMGC GSK
G1T8E2 RABIT#CMGC GSK
A0A1U8BRN3 MESAU#CMGC GSK
GSK3B MOUSE#CMGC GSK
GSK3B RAT#CMGC GSK
H2P9M0 PONAB#CMGC GSK
F6SA86 HORSE#CMGC GSK
A0A287AQ89 PIG#CMGC GSK
CDKL5 HUMAN@CMGC CDKL
CDKL5 MOUSE#CMGC CDKL
CDKL1 HUMAN@CMGC CDKL
CDKL4 HUMAN@CMGC CDKL
CDKL2 HUMAN@CMGC CDKL
CDKL3 HUMAN@CMGC CDKL
CDK10 HUMAN@CMGC CDK CDK10
CD11B MOUSE#CMGC CDK CDK11
CD11B HUMAN@CMGC CDK CDK11
CD11B HUMAN%CMGC CDK CDK11
CD11A HUMAN#CMGC CDK CDK11
CDK13 HUMAN@CMGC CDK CRK7
CDK12 HUMAN@CMGC CDK CRK7
CDK12 RAT#CMGC CDK CRK7
BUR1 YEAST#CMGC CLK
CDK9 CAEEL#CMGC CDK CDK9
CDK9 DANRE#CMGC CDK CDK9
CDK9 CHICK#CMGC CDK CDK9
CDK9 HUMAN@CMGC CDK CDK9
CDK9 BOVIN#CMGC CDK CDK9
CDK9 MOUSE#CMGC CDK CDK9
CDK9 RAT#CMGC CDK CDK9
SSN3 YEAST#CMGC CDK CDK8
CDK19 HUMAN@CMGC CDK CDK8
CDK8 HUMAN@CMGC CDK CDK8
CDK8 MOUSE#CMGC CDK CDK8
CDK4 HUMAN@CMGC CDK CDK4
CDK4 PIG#CMGC CDK CDK4
CDK4 MOUSE#CMGC CDK CDK4
CDK4 RAT#CMGC CDK CDK4
CDK6 HUMAN@CMGC CDK CDK4
CDK6 MOUSE#CMGC CDK CDK4
F1MA87 RAT#CMGC CDK CDK4
CDK1 SCHPO#CMGC CDK CDC2
CDK1 CANAL#CMGC CDK CDC2
CDK1 YEAST#CMGC CDK CDC2
CDKA1 ARATH#CMGC CDK CDC2
CDK1 CARAU#CMGC CDK CDC2
CDK1 CHICK#CMGC CDK CDC2
F1NBD7 CHICK#CMGC CDK CDC2
CDK1 HUMAN@CMGC CDK CDC2
CDK1 BOVIN#CMGC CDK CDC2
G1SIH0 RABIT#CMGC CDK CDC2
CDK1 MOUSE#CMGC CDK CDC2
CDK1 RAT#CMGC CDK CDC2
CDK1 PONAB#CMGC CDK CDC2
CDK1 DROME#CMGC CDK CDC2
CDK1 CAEBR#CMGC CDK CDC2
CDK1 CAEEL#CMGC CDK CDC2
CDK2 XENLA#CMGC CDK CDK2
CDK2 CARAU#CMGC CDK CDK2
CDK2 CRIGR#CMGC CDK CDK2
CDK2 MESAU#CMGC CDK CDK2
CDK2 HUMAN#CMGC CDK CDK2
CDK2 RAT#CMGC CDK CDK2
CDK2 MOUSE#CMGC CDK CDK2
CDK2 BOVIN#CMGC CDK CDK2
CDK3 HUMAN@CMGC CDK CDK2
CDK3 MOUSE#CMGC CDK CDK2
CDK18 HUMAN@CMGC CDK PCTAIRE
CDK17 HUMAN@CMGC CDK PCTAIRE
CDK16 HUMAN@CMGC CDK PCTAIRE
CDK16 RAT#CMGC CDK PCTAIRE
CDK16 MOUSE#CMGC CDK PCTAIRE
CDK14 HUMAN@CMGC CDK PFTAIRE
CDK14 MOUSE#CMGC CDK PFTAIRE
CDK15 HUMAN@CMGC CDK PFTAIRE
CDK15 MOUSE#CMGC CDK PFTAIRE
PHO85 YEAST#CMGC CDK CDK5

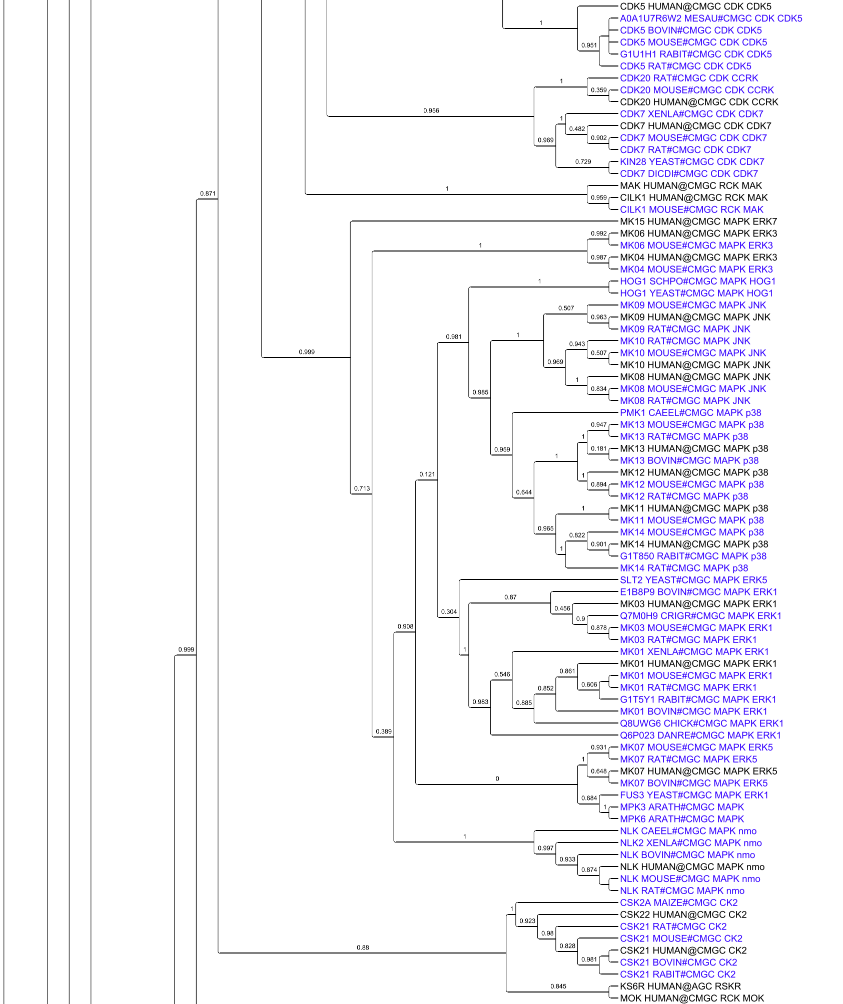
CDK5 HUMAN@CMGC CDK CDK5
A0A1U7R6W2 MESAU#CMGC CDK CDK5
CDK5 BOVIN#CMGC CDK CDK5
CDK5 MOUSE#CMGC CDK CDK5
G1U1H1 RABIT#CMGC CDK CDK5
CDK5 RAT#CMGC CDK CDK5
CDK20 RAT#CMGC CDK CCRK
CDK20 MOUSE#CMGC CDK CCRK
CDK20 HUMAN@CMGC CDK CCRK
CDK7 XENLA#CMGC CDK CDK7
CDK7 HUMAN@CMGC CDK CDK7
CDK7 MOUSE#CMGC CDK CDK7
CDK7 RAT#CMGC CDK CDK7
KIN28 YEAST#CMGC CDK CDK7
CDK7 DICDI#CMGC CDK CDK7
MAK HUMAN@CMGC RCK MAK
CILK1 HUMAN@CMGC RCK MAK
CILK1 MOUSE#CMGC RCK MAK
MK15 HUMAN@CMGC MAPK ERK7
MK06 HUMAN@CMGC MAPK ERK3
MK06 MOUSE#CMGC MAPK ERK3
MK04 HUMAN@CMGC MAPK ERK3
MK04 MOUSE#CMGC MAPK ERK3
HOG1 SCHPO#CMGC MAPK HOG1
HOG1 YEAST#CMGC MAPK HOG1
MK09 MOUSE#CMGC MAPK JNK
MK09 HUMAN@CMGC MAPK JNK
MK09 RAT#CMGC MAPK JNK
MK10 RAT#CMGC MAPK JNK
MK10 MOUSE#CMGC MAPK JNK
MK10 HUMAN@CMGC MAPK JNK
MK08 HUMAN@CMGC MAPK JNK
MK08 MOUSE#CMGC MAPK JNK
MK08 RAT#CMGC MAPK JNK
PMK1 CAEEL#CMGC MAPK p38
MK13 MOUSE#CMGC MAPK p38
MK13 RAT#CMGC MAPK p38
MK13 HUMAN@CMGC MAPK p38
MK13 BOVIN#CMGC MAPK p38
MK12 HUMAN@CMGC MAPK p38
MK12 MOUSE#CMGC MAPK p38
MK12 RAT#CMGC MAPK p38
MK11 HUMAN@CMGC MAPK p38
MK11 MOUSE#CMGC MAPK p38
MK14 MOUSE#CMGC MAPK p38
MK14 HUMAN@CMGC MAPK p38
G1T850 RABIT#CMGC MAPK p38
MK14 RAT#CMGC MAPK p38
SLT2 YEAST#CMGC MAPK ERK5
E1B8P9 BOVIN#CMGC MAPK ERK1
MK03 HUMAN@CMGC MAPK ERK1
Q7M0H9 CRIGR#CMGC MAPK ERK1
MK03 MOUSE#CMGC MAPK ERK1
MK03 RAT#CMGC MAPK ERK1
MK01 XENLA#CMGC MAPK ERK1
MK01 HUMAN@CMGC MAPK ERK1
MK01 MOUSE#CMGC MAPK ERK1
MK01 RAT#CMGC MAPK ERK1
G1T5Y1 RABIT#CMGC MAPK ERK1
MK01 BOVIN#CMGC MAPK ERK1
Q8UWG6 CHICK#CMGC MAPK ERK1
Q6P023 DANRE#CMGC MAPK ERK1
MK07 MOUSE#CMGC MAPK ERK5
MK07 RAT#CMGC MAPK ERK5
MK07 HUMAN@CMGC MAPK ERK5
MK07 BOVIN#CMGC MAPK ERK5
FUS3 YEAST#CMGC MAPK ERK1
MPK3 ARATH#CMGC MAPK
MPK6 ARATH#CMGC MAPK
NLK CAEEL#CMGC MAPK nmo
NLK2 XENLA#CMGC MAPK nmo
NLK BOVIN#CMGC MAPK nmo
NLK HUMAN@CMGC MAPK nmo
NLK MOUSE#CMGC MAPK nmo
NLK RAT#CMGC MAPK nmo
CSK2A MAIZE#CMGC CK2
CSK22 HUMAN@CMGC CK2
CSK21 RAT#CMGC CK2
CSK21 MOUSE#CMGC CK2
CSK21 HUMAN@CMGC CK2
CSK21 BOVIN#CMGC CK2
CSK21 RABIT#CMGC CK2
KS6R HUMAN@AGC RSKR
MOK HUMAN@CMGC RCK MOK

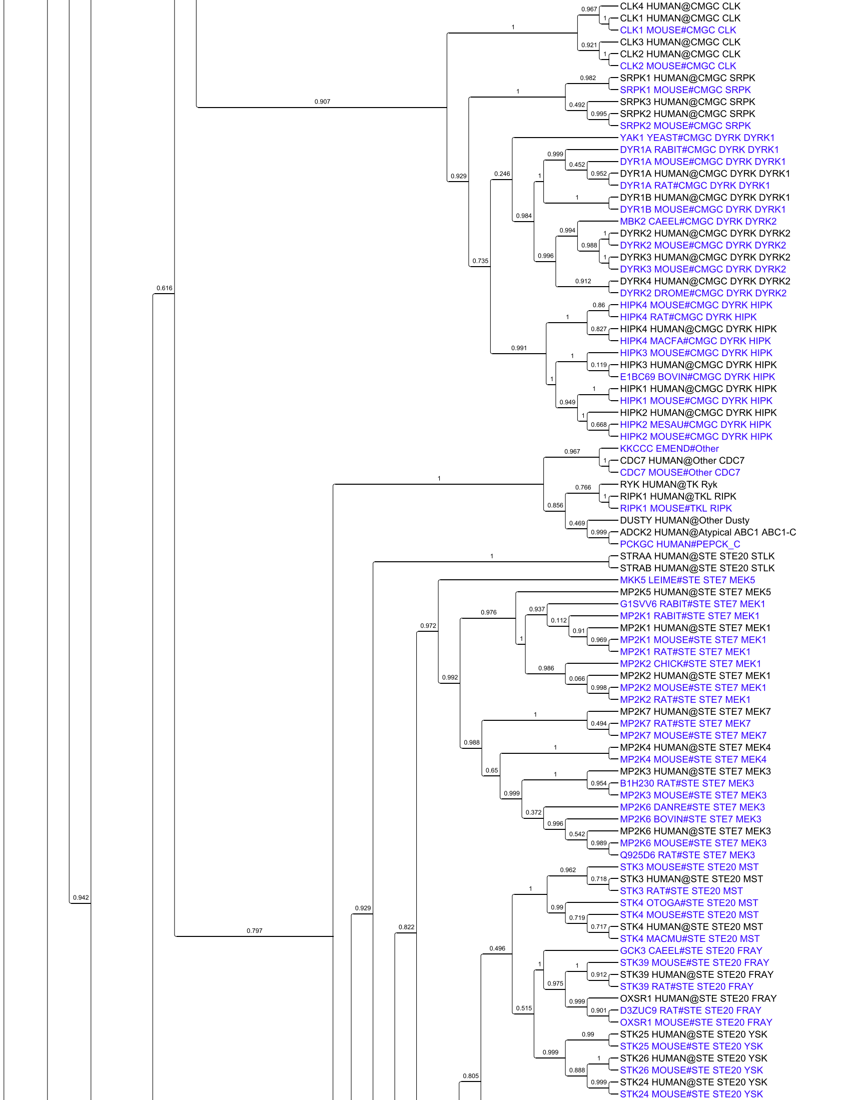
CLK4 HUMAN@CMGC CLK
CLK1 HUMAN@CMGC CLK
CLK1 MOUSE#CMGC CLK
CLK3 HUMAN@CMGC CLK
CLK2 HUMAN@CMGC CLK
CLK2 MOUSE#CMGC CLK
SRPK1 HUMAN@CMGC SRPK
SRPK1 MOUSE#CMGC SRPK
SRPK3 HUMAN@CMGC SRPK
SRPK2 HUMAN@CMGC SRPK
SRPK2 MOUSE#CMGC SRPK
YAK1 YEAST#CMGC DYRK DYRK1
DYR1A RABIT#CMGC DYRK DYRK1
DYR1A MOUSE#CMGC DYRK DYRK1
DYR1A HUMAN@CMGC DYRK DYRK1
DYR1A RAT#CMGC DYRK DYRK1
DYR1B HUMAN@CMGC DYRK DYRK1
DYR1B MOUSE#CMGC DYRK DYRK1
MBK2 CAEEL#CMGC DYRK DYRK2
DYRK2 HUMAN@CMGC DYRK DYRK2
DYRK2 MOUSE#CMGC DYRK DYRK2
DYRK3 HUMAN@CMGC DYRK DYRK2
DYRK3 MOUSE#CMGC DYRK DYRK2
DYRK4 HUMAN@CMGC DYRK DYRK2
DYRK2 DROME#CMGC DYRK DYRK2
HIPK4 MOUSE#CMGC DYRK HIPK
HIPK4 RAT#CMGC DYRK HIPK
HIPK4 HUMAN@CMGC DYRK HIPK
HIPK4 MACFA#CMGC DYRK HIPK
HIPK3 MOUSE#CMGC DYRK HIPK
HIPK3 HUMAN@CMGC DYRK HIPK
E1BC69 BOVIN#CMGC DYRK HIPK
HIPK1 HUMAN@CMGC DYRK HIPK
HIPK1 MOUSE#CMGC DYRK HIPK
HIPK2 HUMAN@CMGC DYRK HIPK
HIPK2 MESAU#CMGC DYRK HIPK
HIPK2 MOUSE#CMGC DYRK HIPK
KKCCC EMEND#Other
CDC7 HUMAN@Other CDC7
CDC7 MOUSE#Other CDC7
RYK HUMAN@TK Ryk
RIPK1 HUMAN@TKL RIPK
RIPK1 MOUSE#TKL RIPK
DUSTY HUMAN@Other Dusty
ADCK2 HUMAN@Atypical ABC1 ABC1-C
PCKGC HUMAN#PEPCK_C
STRAA HUMAN@STE STE20 STLK
STRAB HUMAN@STE STE20 STLK
MKK5 LEIME#STE STE7 MEK5
MP2K5 HUMAN@STE STE7 MEK5
G1SVV6 RABIT#STE STE7 MEK1
MP2K1 RABIT#STE STE7 MEK1
MP2K1 HUMAN@STE STE7 MEK1
MP2K1 MOUSE#STE STE7 MEK1
MP2K1 RAT#STE STE7 MEK1
MP2K2 CHICK#STE STE7 MEK1
MP2K2 HUMAN@STE STE7 MEK1
MP2K2 MOUSE#STE STE7 MEK1
MP2K2 RAT#STE STE7 MEK1
MP2K7 HUMAN@STE STE7 MEK7
MP2K7 RAT#STE STE7 MEK7
MP2K7 MOUSE#STE STE7 MEK7
MP2K4 HUMAN@STE STE7 MEK4
MP2K4 MOUSE#STE STE7 MEK4
MP2K3 HUMAN@STE STE7 MEK3
B1H230 RAT#STE STE7 MEK3
MP2K3 MOUSE#STE STE7 MEK3
MP2K6 DANRE#STE STE7 MEK3
MP2K6 BOVIN#STE STE7 MEK3
MP2K6 HUMAN@STE STE7 MEK3
MP2K6 MOUSE#STE STE7 MEK3
Q925D6 RAT#STE STE7 MEK3
STK3 MOUSE#STE STE20 MST
STK3 HUMAN@STE STE20 MST
STK3 RAT#STE STE20 MST
STK4 OTOGA#STE STE20 MST
STK4 MOUSE#STE STE20 MST
STK4 HUMAN@STE STE20 MST
STK4 MACMU#STE STE20 MST
GCK3 CAEEL#STE STE20 FRAY
STK39 MOUSE#STE STE20 FRAY
STK39 HUMAN@STE STE20 FRAY
STK39 RAT#STE STE20 FRAY
OXSR1 HUMAN@STE STE20 FRAY
D3ZUC9 RAT#STE STE20 FRAY
OXSR1 MOUSE#STE STE20 FRAY
STK25 HUMAN@STE STE20 YSK
STK25 MOUSE#STE STE20 YSK
STK26 HUMAN@STE STE20 YSK
STK26 MOUSE#STE STE20 YSK
STK24 HUMAN@STE STE20 YSK
STK24 MOUSE#STE STE20 YSK

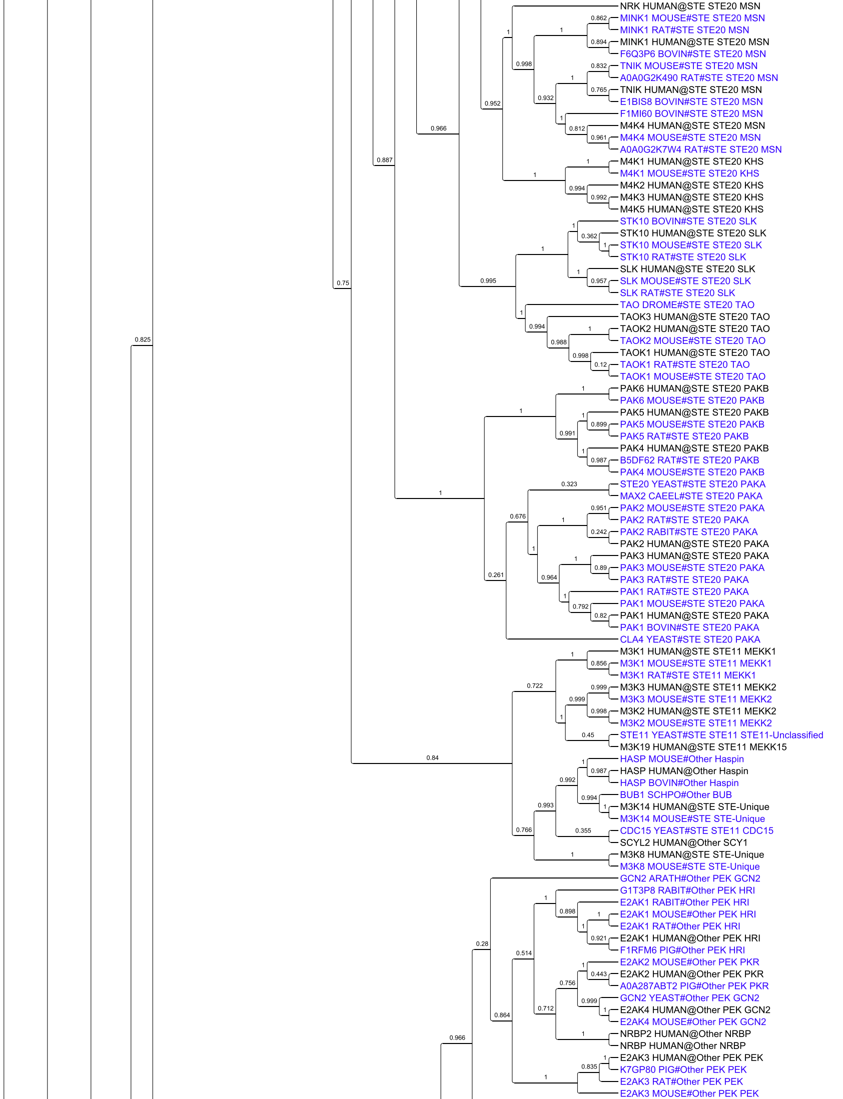
NRK HUMAN@STE STE20 MSN
MINK1 MOUSE#STE STE20 MSN
MINK1 RAT#STE STE20 MSN
MINK1 HUMAN@STE STE20 MSN
F6Q3P6 BOVIN#STE STE20 MSN
TNIK MOUSE#STE STE20 MSN
A0A0G2K490 RAT#STE STE20 MSN
TNIK HUMAN@STE STE20 MSN
E1BIS8 BOVIN#STE STE20 MSN
F1MI60 BOVIN#STE STE20 MSN
M4K4 HUMAN@STE STE20 MSN
M4K4 MOUSE#STE STE20 MSN
A0A0G2K7W4 RAT#STE STE20 MSN
M4K1 HUMAN@STE STE20 KHS
M4K1 MOUSE#STE STE20 KHS
M4K2 HUMAN@STE STE20 KHS
M4K3 HUMAN@STE STE20 KHS
M4K5 HUMAN@STE STE20 KHS
STK10 BOVIN#STE STE20 SLK
STK10 HUMAN@STE STE20 SLK
STK10 MOUSE#STE STE20 SLK
STK10 RAT#STE STE20 SLK
SLK HUMAN@STE STE20 SLK
SLK MOUSE#STE STE20 SLK
SLK RAT#STE STE20 SLK
TAO DROME#STE STE20 TAO
TAOK3 HUMAN@STE STE20 TAO
TAOK2 HUMAN@STE STE20 TAO
TAOK2 MOUSE#STE STE20 TAO
TAOK1 HUMAN@STE STE20 TAO
TAOK1 RAT#STE STE20 TAO
TAOK1 MOUSE#STE STE20 TAO
PAK6 HUMAN@STE STE20 PAKB
PAK6 MOUSE#STE STE20 PAKB
PAK5 HUMAN@STE STE20 PAKB
PAK5 MOUSE#STE STE20 PAKB
PAK5 RAT#STE STE20 PAKB
PAK4 HUMAN@STE STE20 PAKB
B5DF62 RAT#STE STE20 PAKB
PAK4 MOUSE#STE STE20 PAKB
STE20 YEAST#STE STE20 PAKA
MAX2 CAEEL#STE STE20 PAKA
PAK2 MOUSE#STE STE20 PAKA
PAK2 RAT#STE STE20 PAKA
PAK2 RABIT#STE STE20 PAKA
PAK2 HUMAN@STE STE20 PAKA
PAK3 HUMAN@STE STE20 PAKA
PAK3 MOUSE#STE STE20 PAKA
PAK3 RAT#STE STE20 PAKA
PAK1 RAT#STE STE20 PAKA
PAK1 MOUSE#STE STE20 PAKA
PAK1 HUMAN@STE STE20 PAKA
PAK1 BOVIN#STE STE20 PAKA
CLA4 YEAST#STE STE20 PAKA
M3K1 HUMAN@STE STE11 MEKK1
M3K1 MOUSE#STE STE11 MEKK1
M3K1 RAT#STE STE11 MEKK1
M3K3 HUMAN@STE STE11 MEKK2
M3K3 MOUSE#STE STE11 MEKK2
M3K2 HUMAN@STE STE11 MEKK2
M3K2 MOUSE#STE STE11 MEKK2
STE11 YEAST#STE STE11 STE11-Unclassified
M3K19 HUMAN@STE STE11 MEKK15
HASP MOUSE#Other Haspin
HASP HUMAN@Other Haspin
HASP BOVIN#Other Haspin
BUB1 SCHPO#Other BUB
M3K14 HUMAN@STE STE-Unique
M3K14 MOUSE#STE STE-Unique
CDC15 YEAST#STE STE11 CDC15
SCYL2 HUMAN@Other SCY1
M3K8 HUMAN@STE STE-Unique
M3K8 MOUSE#STE STE-Unique
GCN2 ARATH#Other PEK GCN2
G1T3P8 RABIT#Other PEK HRI
E2AK1 RABIT#Other PEK HRI
E2AK1 MOUSE#Other PEK HRI
E2AK1 RAT#Other PEK HRI
E2AK1 HUMAN@Other PEK HRI
F1RFM6 PIG#Other PEK HRI
E2AK2 MOUSE#Other PEK PKR
E2AK2 HUMAN@Other PEK PKR
A0A287ABT2 PIG#Other PEK PKR
GCN2 YEAST#Other PEK GCN2
E2AK4 HUMAN@Other PEK GCN2
E2AK4 MOUSE#Other PEK GCN2
NRBP2 HUMAN@Other NRBP
NRBP HUMAN@Other NRBP
E2AK3 HUMAN@Other PEK PEK
K7GP80 PIG#Other PEK PEK
E2AK3 RAT#Other PEK PEK
E2AK3 MOUSE#Other PEK PEK

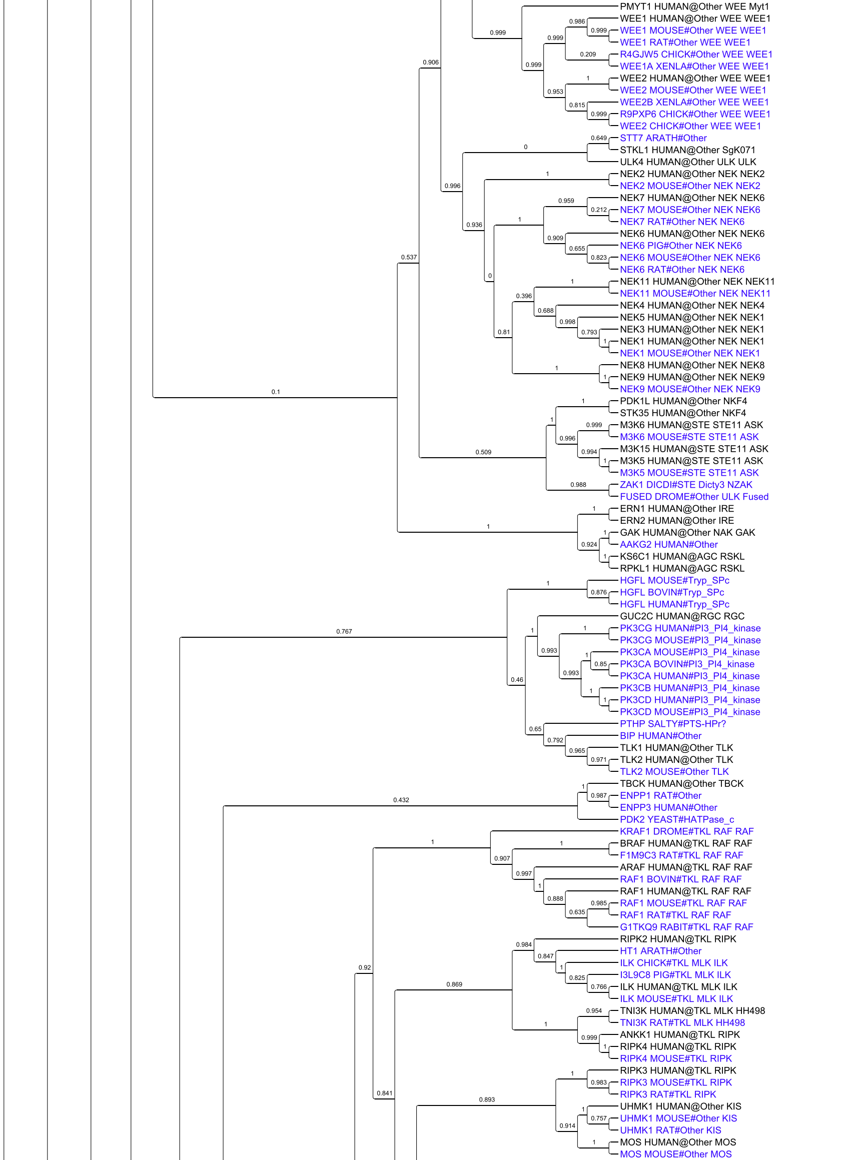
PMYT1 HUMAN@Other WEE Myt1
WEE1 HUMAN@Other WEE WEE1
WEE1 MOUSE#Other WEE WEE1
WEE1 RAT#Other WEE WEE1
R4GJW5 CHICK#Other WEE WEE1
WEE1A XENLA#Other WEE WEE1
WEE2 HUMAN@Other WEE WEE1
WEE2 MOUSE#Other WEE WEE1
WEE2B XENLA#Other WEE WEE1
R9PXP6 CHICK#Other WEE WEE1
WEE2 CHICK#Other WEE WEE1
STT7 ARATH#Other
STKL1 HUMAN@Other SgK071
ULK4 HUMAN@Other ULK ULK
NEK2 HUMAN@Other NEK NEK2
NEK2 MOUSE#Other NEK NEK2
NEK7 HUMAN@Other NEK NEK6
NEK7 MOUSE#Other NEK NEK6
NEK7 RAT#Other NEK NEK6
NEK6 HUMAN@Other NEK NEK6
NEK6 PIG#Other NEK NEK6
NEK6 MOUSE#Other NEK NEK6
NEK6 RAT#Other NEK NEK6
NEK11 HUMAN@Other NEK NEK11
NEK11 MOUSE#Other NEK NEK11
NEK4 HUMAN@Other NEK NEK4
NEK5 HUMAN@Other NEK NEK1
NEK3 HUMAN@Other NEK NEK1
NEK1 HUMAN@Other NEK NEK1
NEK1 MOUSE#Other NEK NEK1
NEK8 HUMAN@Other NEK NEK8
NEK9 HUMAN@Other NEK NEK9
NEK9 MOUSE#Other NEK NEK9
PDK1L HUMAN@Other NKF4
STK35 HUMAN@Other NKF4
M3K6 HUMAN@STE STE11 ASK
M3K6 MOUSE#STE STE11 ASK
M3K15 HUMAN@STE STE11 ASK
M3K5 HUMAN@STE STE11 ASK
M3K5 MOUSE#STE STE11 ASK
ZAK1 DICDI#STE Dicty3 NZAK
FUSED DROME#Other ULK Fused
ERN1 HUMAN@Other IRE
ERN2 HUMAN@Other IRE
GAK HUMAN@Other NAK GAK
AAKG2 HUMAN#Other
KS6C1 HUMAN@AGC RSKL
RPKL1 HUMAN@AGC RSKL
HGFL MOUSE#Tryp_SPc
HGFL BOVIN#Tryp_SPc
HGFL HUMAN#Tryp_SPc
GUC2C HUMAN@RGC RGC
PK3CG HUMAN#PI3_PI4_kinase
PK3CG MOUSE#PI3_PI4_kinase
PK3CA MOUSE#PI3_PI4_kinase
PK3CA BOVIN#PI3_PI4_kinase
PK3CA HUMAN#PI3_PI4_kinase
PK3CB HUMAN#PI3_PI4_kinase
PK3CD HUMAN#PI3_PI4_kinase
PK3CD MOUSE#PI3_PI4_kinase
PTHP SALTY#PTS-HPr?
BIP HUMAN#Other
TLK1 HUMAN@Other TLK
TLK2 HUMAN@Other TLK
TLK2 MOUSE#Other TLK
TBCK HUMAN@Other TBCK
ENPP1 RAT#Other
ENPP3 HUMAN#Other
PDK2 YEAST#HATPase_c
KRAF1 DROME#TKL RAF RAF
BRAF HUMAN@TKL RAF RAF
F1M9C3 RAT#TKL RAF RAF
ARAF HUMAN@TKL RAF RAF
RAF1 BOVIN#TKL RAF RAF
RAF1 HUMAN@TKL RAF RAF
RAF1 MOUSE#TKL RAF RAF
RAF1 RAT#TKL RAF RAF
G1TKQ9 RABIT#TKL RAF RAF
RIPK2 HUMAN@TKL RIPK
HT1 ARATH#Other
ILK CHICK#TKL MLK ILK
I3L9C8 PIG#TKL MLK ILK
ILK HUMAN@TKL MLK ILK
ILK MOUSE#TKL MLK ILK
TNI3K HUMAN@TKL MLK HH498
TNI3K RAT#TKL MLK HH498
ANKK1 HUMAN@TKL RIPK
RIPK4 HUMAN@TKL RIPK
RIPK4 MOUSE#TKL RIPK
RIPK3 HUMAN@TKL RIPK
RIPK3 MOUSE#TKL RIPK
RIPK3 RAT#TKL RIPK
UHMK1 HUMAN@Other KIS
UHMK1 MOUSE#Other KIS
UHMK1 RAT#Other KIS
MOS HUMAN@Other MOS
MOS MOUSE#Other MOS

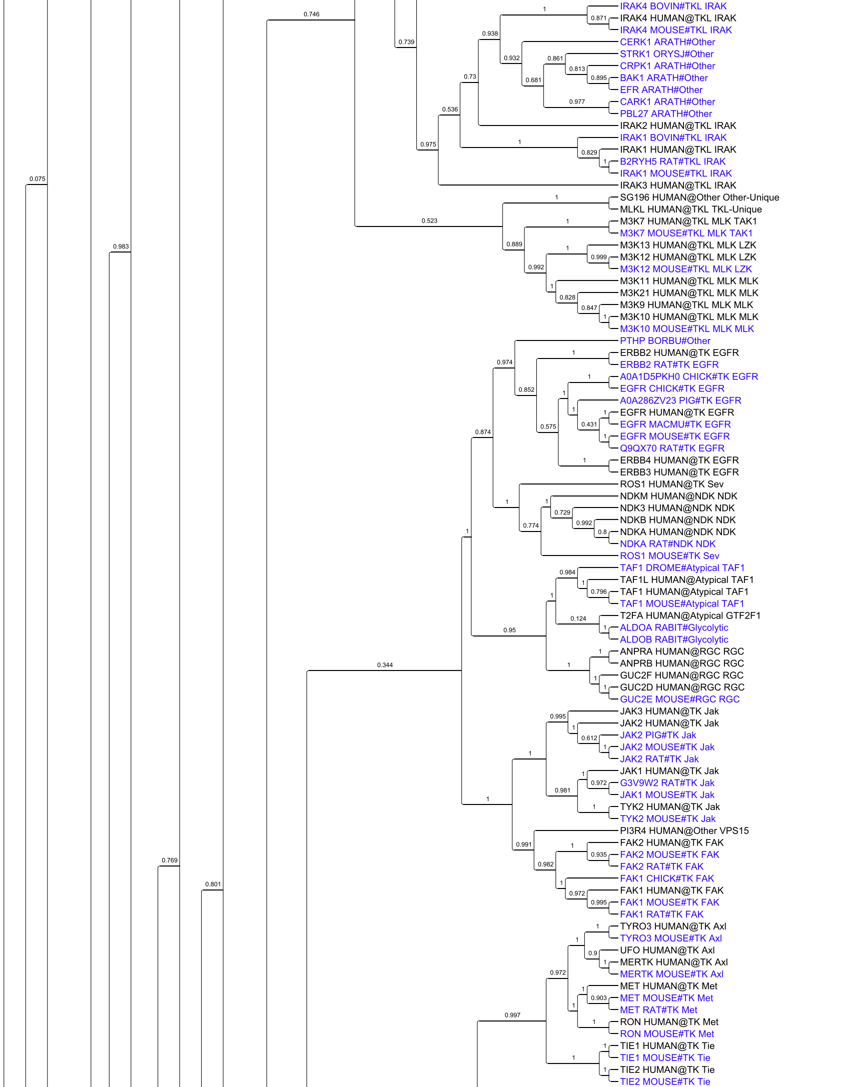
IRAK4 BOVIN#TKL IRAK
IRAK4 HUMAN@TKL IRAK
IRAK4 MOUSE#TKL IRAK
CERK1 ARATH#Other
STRK1 ORYSJ#Other
CRPK1 ARATH#Other
BAK1 ARATH#Other
EFR ARATH#Other
CARK1 ARATH#Other
PBL27 ARATH#Other
IRAK2 HUMAN@TKL IRAK
IRAK1 BOVIN#TKL IRAK
IRAK1 HUMAN@TKL IRAK
B2RYH5 RAT#TKL IRAK
IRAK1 MOUSE#TKL IRAK
IRAK3 HUMAN@TKL IRAK
SG196 HUMAN@Other Other-Unique
MLKL HUMAN@TKL TKL-Unique
M3K7 HUMAN@TKL MLK TAK1
M3K7 MOUSE#TKL MLK TAK1
M3K13 HUMAN@TKL MLK LZK
M3K12 HUMAN@TKL MLK LZK
M3K12 MOUSE#TKL MLK LZK
M3K11 HUMAN@TKL MLK MLK
M3K21 HUMAN@TKL MLK MLK
M3K9 HUMAN@TKL MLK MLK
M3K10 HUMAN@TKL MLK MLK
M3K10 MOUSE#TKL MLK MLK
PTHP BORBU#Other
ERBB2 HUMAN@TK EGFR
ERBB2 RAT#TK EGFR
A0A1D5PKH0 CHICK#TK EGFR
EGFR CHICK#TK EGFR
A0A286ZV23 PIG#TK EGFR
EGFR HUMAN@TK EGFR
EGFR MACMU#TK EGFR
EGFR MOUSE#TK EGFR
Q9QX70 RAT#TK EGFR
ERBB4 HUMAN@TK EGFR
ERBB3 HUMAN@TK EGFR
ROS1 HUMAN@TK Sev
NDKM HUMAN@NDK NDK
NDK3 HUMAN@NDK NDK
NDKB HUMAN@NDK NDK
NDKA HUMAN@NDK NDK
NDKA RAT#NDK NDK
ROS1 MOUSE#TK Sev
TAF1 DROME#Atypical TAF1
TAF1L HUMAN@Atypical TAF1
TAF1 HUMAN@Atypical TAF1
TAF1 MOUSE#Atypical TAF1
T2FA HUMAN@Atypical GTF2F1
ALDOA RABIT#Glycolytic
ALDOB RABIT#Glycolytic
ANPRA HUMAN@RGC RGC
ANPRB HUMAN@RGC RGC
GUC2F HUMAN@RGC RGC
GUC2D HUMAN@RGC RGC
GUC2E MOUSE#RGC RGC
JAK3 HUMAN@TK Jak
JAK2 HUMAN@TK Jak
JAK2 PIG#TK Jak
JAK2 MOUSE#TK Jak
JAK2 RAT#TK Jak
JAK1 HUMAN@TK Jak
G3V9W2 RAT#TK Jak
JAK1 MOUSE#TK Jak
TYK2 HUMAN@TK Jak
TYK2 MOUSE#TK Jak
PI3R4 HUMAN@Other VPS15
FAK2 HUMAN@TK FAK
FAK2 MOUSE#TK FAK
FAK2 RAT#TK FAK
FAK1 CHICK#TK FAK
FAK1 HUMAN@TK FAK
FAK1 MOUSE#TK FAK
FAK1 RAT#TK FAK
TYRO3 HUMAN@TK Axl
TYRO3 MOUSE#TK Axl
UFO HUMAN@TK Axl
MERTK HUMAN@TK Axl
MERTK MOUSE#TK Axl
MET HUMAN@TK Met
MET MOUSE#TK Met
MET RAT#TK Met
RON HUMAN@TK Met
RON MOUSE#TK Met
TIE1 HUMAN@TK Tie
TIE1 MOUSE#TK Tie
TIE2 HUMAN@TK Tie
TIE2 MOUSE#TK Tie

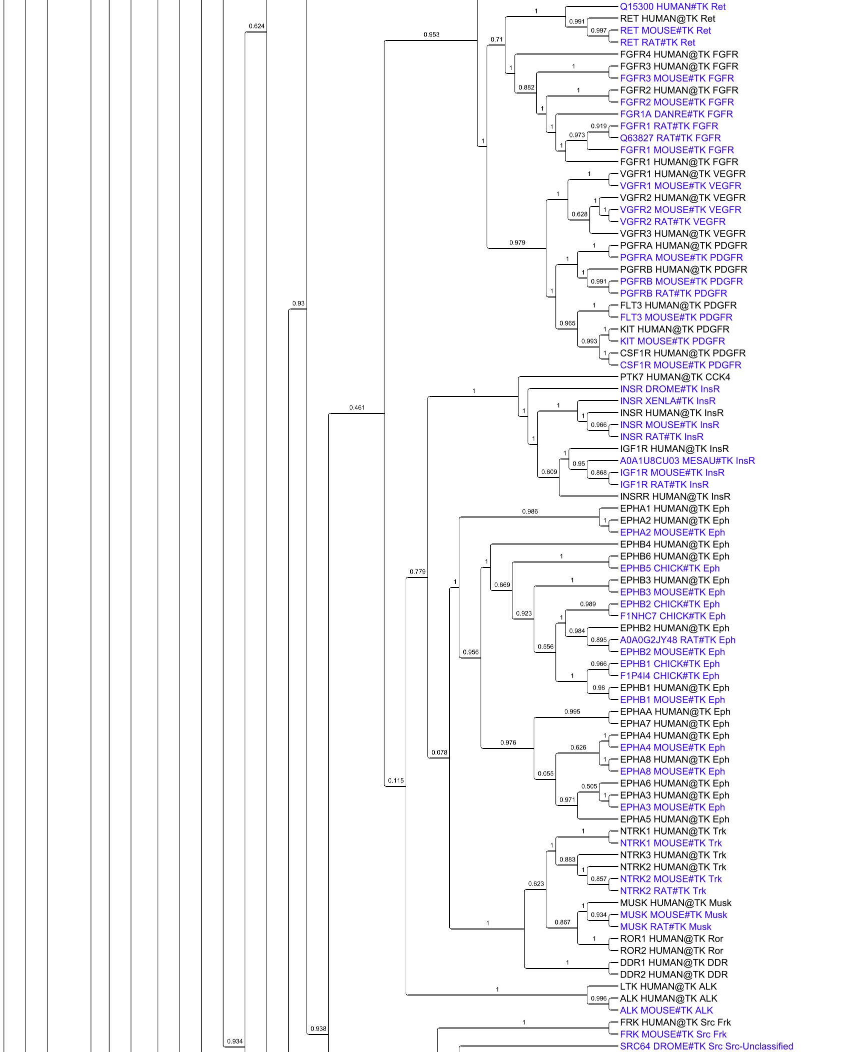
Q15300 HUMAN#TK Ret
RET HUMAN@TK Ret
RET MOUSE#TK Ret
RET RAT#TK Ret
FGFR4 HUMAN@TK FGFR
FGFR3 HUMAN@TK FGFR
FGFR3 MOUSE#TK FGFR
FGFR2 HUMAN@TK FGFR
FGFR2 MOUSE#TK FGFR
FGR1A DANRE#TK FGFR
FGFR1 RAT#TK FGFR
Q63827 RAT#TK FGFR
FGFR1 MOUSE#TK FGFR
FGFR1 HUMAN@TK FGFR
VGFR1 HUMAN@TK VEGFR
VGFR1 MOUSE#TK VEGFR
VGFR2 HUMAN@TK VEGFR
VGFR2 MOUSE#TK VEGFR
VGFR2 RAT#TK VEGFR
VGFR3 HUMAN@TK VEGFR
PGFRA HUMAN@TK PDGFR
PGFRA MOUSE#TK PDGFR
PGFRB HUMAN@TK PDGFR
PGFRB MOUSE#TK PDGFR
PGFRB RAT#TK PDGFR
FLT3 HUMAN@TK PDGFR
FLT3 MOUSE#TK PDGFR
KIT HUMAN@TK PDGFR
KIT MOUSE#TK PDGFR
CSF1R HUMAN@TK PDGFR
CSF1R MOUSE#TK PDGFR
PTK7 HUMAN@TK CCK4
INSR DROME#TK InsR
INSR XENLA#TK InsR
INSR HUMAN@TK InsR
INSR MOUSE#TK InsR
INSR RAT#TK InsR
IGF1R HUMAN@TK InsR
A0A1U8CU03 MESAU#TK InsR
IGF1R MOUSE#TK InsR
IGF1R RAT#TK InsR
INSRR HUMAN@TK InsR
EPHA1 HUMAN@TK Eph
EPHA2 HUMAN@TK Eph
EPHA2 MOUSE#TK Eph
EPHB4 HUMAN@TK Eph
EPHB6 HUMAN@TK Eph
EPHB5 CHICK#TK Eph
EPHB3 HUMAN@TK Eph
EPHB3 MOUSE#TK Eph
EPHB2 CHICK#TK Eph
F1NHC7 CHICK#TK Eph
EPHB2 HUMAN@TK Eph
A0A0G2JY48 RAT#TK Eph
EPHB2 MOUSE#TK Eph
EPHB1 CHICK#TK Eph
F1P4I4 CHICK#TK Eph
EPHB1 HUMAN@TK Eph
EPHB1 MOUSE#TK Eph
EPHAA HUMAN@TK Eph
EPHA7 HUMAN@TK Eph
EPHA4 HUMAN@TK Eph
EPHA4 MOUSE#TK Eph
EPHA8 HUMAN@TK Eph
EPHA8 MOUSE#TK Eph
EPHA6 HUMAN@TK Eph
EPHA3 HUMAN@TK Eph
EPHA3 MOUSE#TK Eph
EPHA5 HUMAN@TK Eph
NTRK1 HUMAN@TK Trk
NTRK1 MOUSE#TK Trk
NTRK3 HUMAN@TK Trk
NTRK2 HUMAN@TK Trk
NTRK2 MOUSE#TK Trk
NTRK2 RAT#TK Trk
MUSK HUMAN@TK Musk
MUSK MOUSE#TK Musk
MUSK RAT#TK Musk
ROR1 HUMAN@TK Ror
ROR2 HUMAN@TK Ror
DDR1 HUMAN@TK DDR
DDR2 HUMAN@TK DDR
LTK HUMAN@TK ALK
ALK HUMAN@TK ALK
ALK MOUSE#TK ALK
FRK HUMAN@TK Src Frk
FRK MOUSE#TK Src Frk
SRC64 DROME#TK Src Src-Unclassified

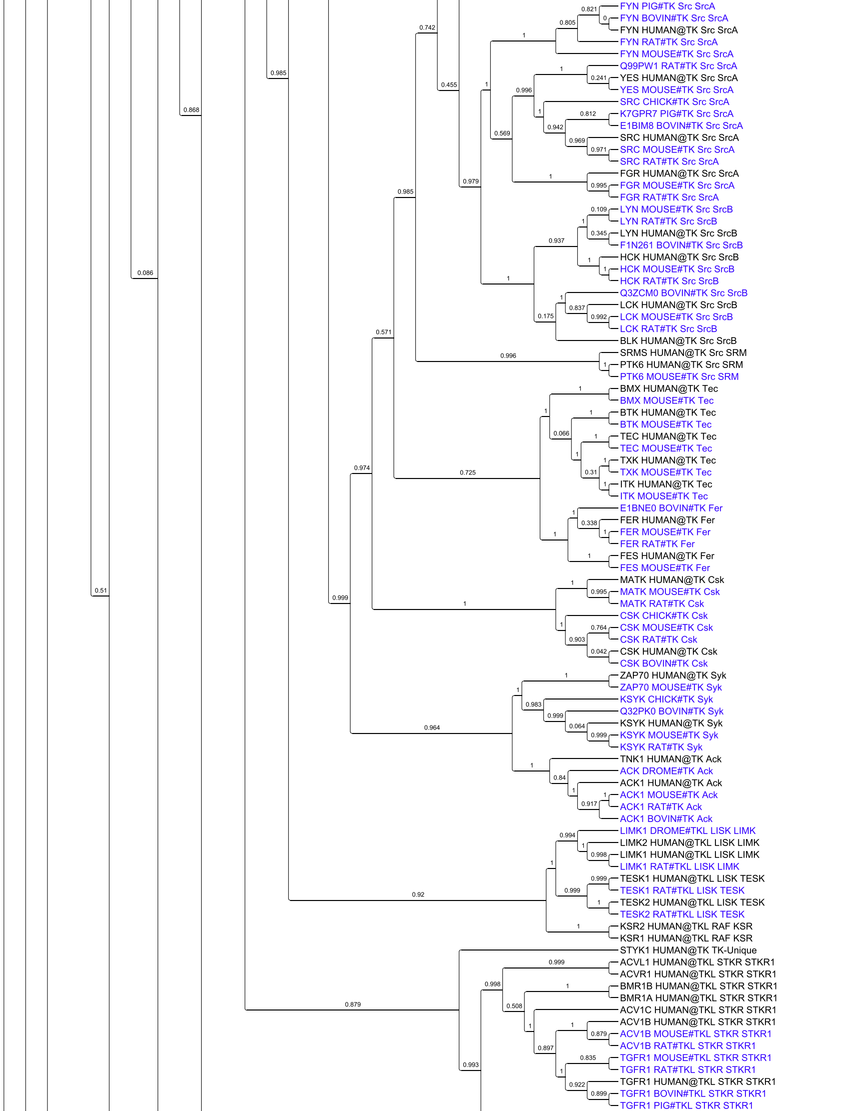
FYN PIG#TK Src SrcA
FYN BOVIN#TK Src SrcA
FYN HUMAN@TK Src SrcA
FYN RAT#TK Src SrcA
FYN MOUSE#TK Src SrcA
Q99PW1 RAT#TK Src SrcA
YES HUMAN@TK Src SrcA
YES MOUSE#TK Src SrcA
SRC CHICK#TK Src SrcA
K7GPR7 PIG#TK Src SrcA
E1BIM8 BOVIN#TK Src SrcA
SRC HUMAN@TK Src SrcA
SRC MOUSE#TK Src SrcA
SRC RAT#TK Src SrcA
FGR HUMAN@TK Src SrcA
FGR MOUSE#TK Src SrcA
FGR RAT#TK Src SrcA
LYN MOUSE#TK Src SrcB
LYN RAT#TK Src SrcB
LYN HUMAN@TK Src SrcB
F1N261 BOVIN#TK Src SrcB
HCK HUMAN@TK Src SrcB
HCK MOUSE#TK Src SrcB
HCK RAT#TK Src SrcB
Q3ZCM0 BOVIN#TK Src SrcB
LCK HUMAN@TK Src SrcB
LCK MOUSE#TK Src SrcB
LCK RAT#TK Src SrcB
BLK HUMAN@TK Src SrcB
SRMS HUMAN@TK Src SRM
PTK6 HUMAN@TK Src SRM
PTK6 MOUSE#TK Src SRM
BMX HUMAN@TK Tec
BMX MOUSE#TK Tec
BTK HUMAN@TK Tec
BTK MOUSE#TK Tec
TEC HUMAN@TK Tec
TEC MOUSE#TK Tec
TXK HUMAN@TK Tec
TXK MOUSE#TK Tec
ITK HUMAN@TK Tec
ITK MOUSE#TK Tec
E1BNE0 BOVIN#TK Fer
FER HUMAN@TK Fer
FER MOUSE#TK Fer
FER RAT#TK Fer
FES HUMAN@TK Fer
FES MOUSE#TK Fer
MATK HUMAN@TK Csk
MATK MOUSE#TK Csk
MATK RAT#TK Csk
CSK CHICK#TK Csk
CSK MOUSE#TK Csk
CSK RAT#TK Csk
CSK HUMAN@TK Csk
CSK BOVIN#TK Csk
ZAP70 HUMAN@TK Syk
ZAP70 MOUSE#TK Syk
KSYK CHICK#TK Syk
Q32PK0 BOVIN#TK Syk
KSYK HUMAN@TK Syk
KSYK MOUSE#TK Syk
KSYK RAT#TK Syk
TNK1 HUMAN@TK Ack
ACK DROME#TK Ack
ACK1 HUMAN@TK Ack
ACK1 MOUSE#TK Ack
ACK1 RAT#TK Ack
ACK1 BOVIN#TK Ack
LIMK1 DROME#TKL LISK LIMK
LIMK2 HUMAN@TKL LISK LIMK
LIMK1 HUMAN@TKL LISK LIMK
LIMK1 RAT#TKL LISK LIMK
TESK1 HUMAN@TKL LISK TESK
TESK1 RAT#TKL LISK TESK
TESK2 HUMAN@TKL LISK TESK
TESK2 RAT#TKL LISK TESK
KSR2 HUMAN@TKL RAF KSR
KSR1 HUMAN@TKL RAF KSR
STYK1 HUMAN@TK TK-Unique
ACVL1 HUMAN@TKL STKR STKR1
ACVR1 HUMAN@TKL STKR STKR1
BMR1B HUMAN@TKL STKR STKR1
BMR1A HUMAN@TKL STKR STKR1
ACV1C HUMAN@TKL STKR STKR1
ACV1B HUMAN@TKL STKR STKR1
ACV1B MOUSE#TKL STKR STKR1
ACV1B RAT#TKL STKR STKR1
TGFR1 MOUSE#TKL STKR STKR1
TGFR1 RAT#TKL STKR STKR1
TGFR1 HUMAN@TKL STKR STKR1
TGFR1 BOVIN#TKL STKR STKR1
TGFR1 PIG#TKL STKR STKR1

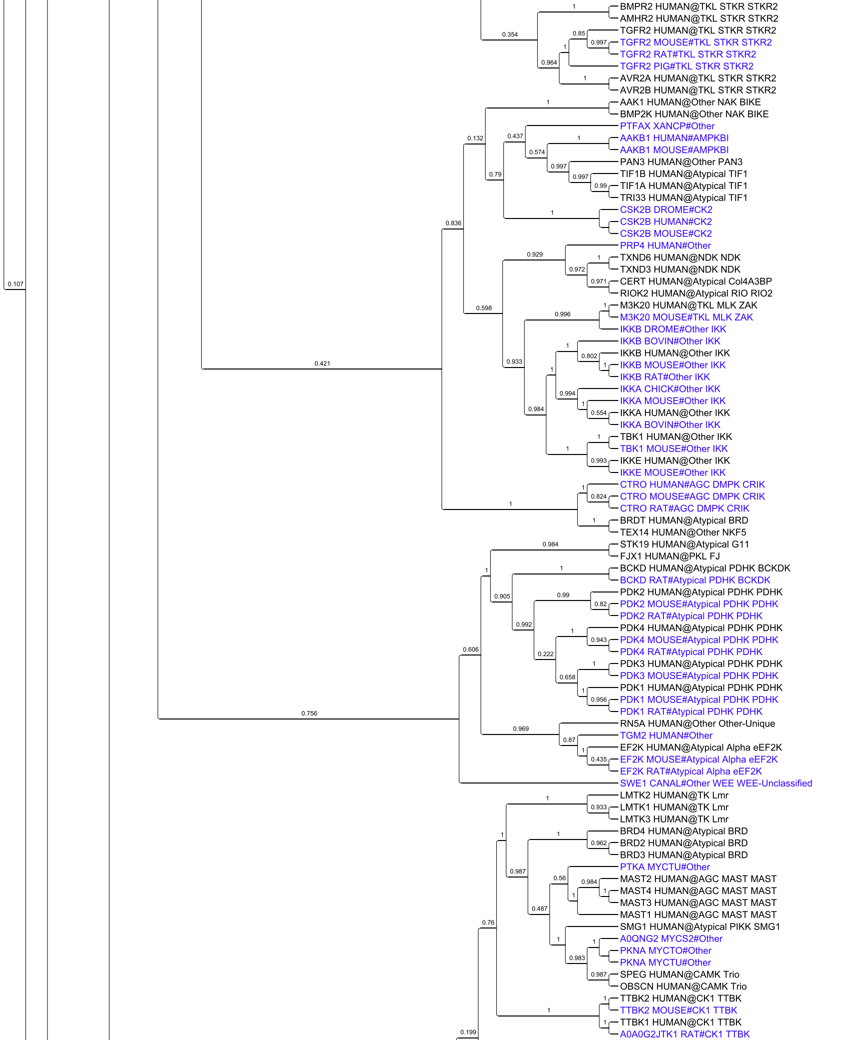

BMPR2 HUMAN@TKL STKR STKR2
AMHR2 HUMAN@TKL STKR STKR2
TGFR2 HUMAN@TKL STKR STKR2
TGFR2 MOUSE#TKL STKR STKR2
TGFR2 RAT#TKL STKR STKR2
TGFR2 PIG#TKL STKR STKR2
AVR2A HUMAN@TKL STKR STKR2
AVR2B HUMAN@TKL STKR STKR2
AAK1 HUMAN@Other NAK BIKE
BMP2K HUMAN@Other NAK BIKE
PTFAX XANCP#Other
AAKB1 HUMAN#AMPKBI
AAKB1 MOUSE#AMPKBI
PAN3 HUMAN@Other PAN3
TIF1B HUMAN@Atypical TIF1
TIF1A HUMAN@Atypical TIF1
TRI33 HUMAN@Atypical TIF1
CSK2B DROME#CK2
CSK2B HUMAN#CK2
CSK2B MOUSE#CK2
PRP4 HUMAN#Other
TXND6 HUMAN@NDK NDK
TXND3 HUMAN@NDK NDK
CERT HUMAN@Atypical Col4A3BP
RIOK2 HUMAN@Atypical RIO RIO2
M3K20 HUMAN@TKL MLK ZAK
M3K20 MOUSE#TKL MLK ZAK
IKKB DROME#Other IKK
IKKB BOVIN#Other IKK
IKKB HUMAN@Other IKK
IKKB MOUSE#Other IKK
IKKB RAT#Other IKK
IKKA CHICK#Other IKK
IKKA MOUSE#Other IKK
IKKA HUMAN@Other IKK
IKKA BOVIN#Other IKK
TBK1 HUMAN@Other IKK
TBK1 MOUSE#Other IKK
IKKE HUMAN@Other IKK
IKKE MOUSE#Other IKK
CTRO HUMAN#AGC DMPK CRIK
CTRO MOUSE#AGC DMPK CRIK
CTRO RAT#AGC DMPK CRIK
BRDT HUMAN@Atypical BRD
TEX14 HUMAN@Other NKF5
STK19 HUMAN@Atypical G11
FJX1 HUMAN@PKL FJ
BCKD HUMAN@Atypical PDHK BCKDK
BCKD RAT#Atypical PDHK BCKDK
PDK2 HUMAN@Atypical PDHK PDHK
PDK2 MOUSE#Atypical PDHK PDHK
PDK2 RAT#Atypical PDHK PDHK
PDK4 HUMAN@Atypical PDHK PDHK
PDK4 MOUSE#Atypical PDHK PDHK
PDK4 RAT#Atypical PDHK PDHK
PDK3 HUMAN@Atypical PDHK PDHK
PDK3 MOUSE#Atypical PDHK PDHK
PDK1 HUMAN@Atypical PDHK PDHK
PDK1 MOUSE#Atypical PDHK PDHK
PDK1 RAT#Atypical PDHK PDHK
RN5A HUMAN@Other Other-Unique
TGM2 HUMAN#Other
EF2K HUMAN@Atypical Alpha eEF2K
EF2K MOUSE#Atypical Alpha eEF2K
EF2K RAT#Atypical Alpha eEF2K
SWE1 CANAL#Other WEE WEE-Unclassified
LMTK2 HUMAN@TK Lmr
LMTK1 HUMAN@TK Lmr
LMTK3 HUMAN@TK Lmr
BRD4 HUMAN@Atypical BRD
BRD2 HUMAN@Atypical BRD
BRD3 HUMAN@Atypical BRD
PTKA MYCTU#Other
MAST2 HUMAN@AGC MAST MAST
MAST4 HUMAN@AGC MAST MAST
MAST3 HUMAN@AGC MAST MAST
MAST1 HUMAN@AGC MAST MAST
SMG1 HUMAN@Atypical PIKK SMG1
A0QNG2 MYCS2#Other
PKNA MYCTO#Other
PKNA MYCTU#Other
SPEG HUMAN@CAMK Trio
OBSCN HUMAN@CAMK Trio
TTBK2 HUMAN@CK1 TTBK
TTBK2 MOUSE#CK1 TTBK
TTBK1 HUMAN@CK1 TTBK
A0A0G2JTK1 RAT#CK1 TTBK

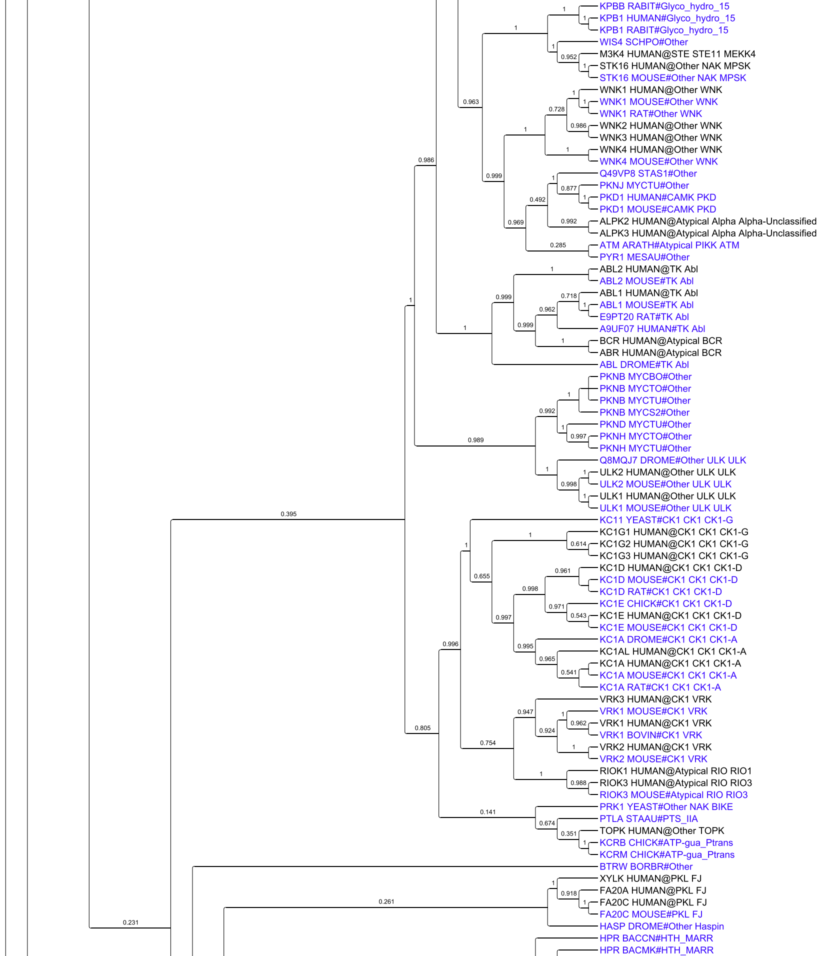
KPBB RABIT#Glyco_hydro_15
KPB1 HUMAN#Glyco_hydro_15
KPB1 RABIT#Glyco_hydro_15
WIS4 SCHPO#Other
M3K4 HUMAN@STE STE11 MEKK4
STK16 HUMAN@Other NAK MPSK
STK16 MOUSE#Other NAK MPSK
WNK1 HUMAN@Other WNK
WNK1 MOUSE#Other WNK
WNK1 RAT#Other WNK
WNK2 HUMAN@Other WNK
WNK3 HUMAN@Other WNK
WNK4 HUMAN@Other WNK
WNK4 MOUSE#Other WNK
Q49VP8 STAS1#Other
PKNJ MYCTU#Other
PKD1 HUMAN#CAMK PKD
PKD1 MOUSE#CAMK PKD
ALPK2 HUMAN@Atypical Alpha Alpha-Unclassified
ALPK3 HUMAN@Atypical Alpha Alpha-Unclassified
ATM ARATH#Atypical PIKK ATM
PYR1 MESAU#Other
ABL2 HUMAN@TK Abl
ABL2 MOUSE#TK Abl
ABL1 HUMAN@TK Abl
ABL1 MOUSE#TK Abl
E9PT20 RAT#TK Abl
A9UF07 HUMAN#TK Abl
BCR HUMAN@Atypical BCR
ABR HUMAN@Atypical BCR
ABL DROME#TK Abl
PKNB MYCBO#Other
PKNB MYCTO#Other
PKNB MYCTU#Other
PKNB MYCS2#Other
PKND MYCTU#Other
PKNH MYCTO#Other
PKNH MYCTU#Other
Q8MQJ7 DROME#Other ULK ULK
ULK2 HUMAN@Other ULK ULK
ULK2 MOUSE#Other ULK ULK
ULK1 HUMAN@Other ULK ULK
ULK1 MOUSE#Other ULK ULK
KC11 YEAST#CK1 CK1 CK1-G
KC1G1 HUMAN@CK1 CK1 CK1-G
KC1G2 HUMAN@CK1 CK1 CK1-G
KC1G3 HUMAN@CK1 CK1 CK1-G
KC1D HUMAN@CK1 CK1 CK1-D
KC1D MOUSE#CK1 CK1 CK1-D
KC1D RAT#CK1 CK1 CK1-D
KC1E CHICK#CK1 CK1 CK1-D
KC1E HUMAN@CK1 CK1 CK1-D
KC1E MOUSE#CK1 CK1 CK1-D
KC1A DROME#CK1 CK1 CK1-A
KC1AL HUMAN@CK1 CK1 CK1-A
KC1A HUMAN@CK1 CK1 CK1-A
KC1A MOUSE#CK1 CK1 CK1-A
KC1A RAT#CK1 CK1 CK1-A
VRK3 HUMAN@CK1 VRK
VRK1 MOUSE#CK1 VRK
VRK1 HUMAN@CK1 VRK
VRK1 BOVIN#CK1 VRK
VRK2 HUMAN@CK1 VRK
VRK2 MOUSE#CK1 VRK
RIOK1 HUMAN@Atypical RIO RIO1
RIOK3 HUMAN@Atypical RIO RIO3
RIOK3 MOUSE#Atypical RIO RIO3
PRK1 YEAST#Other NAK BIKE
PTLA STAAU#PTS_IIA
TOPK HUMAN@Other TOPK
KCRB CHICK#ATP-gua_Ptrans
KCRM CHICK#ATP-gua_Ptrans
BTRW BORBR#Other
XYLK HUMAN@PKL FJ
FA20A HUMAN@PKL FJ
FA20C HUMAN@PKL FJ
FA20C MOUSE#PKL FJ
HASP DROME#Other Haspin
HPR BACCN#HTH_MARR
HPR BACMK#HTH_MARR

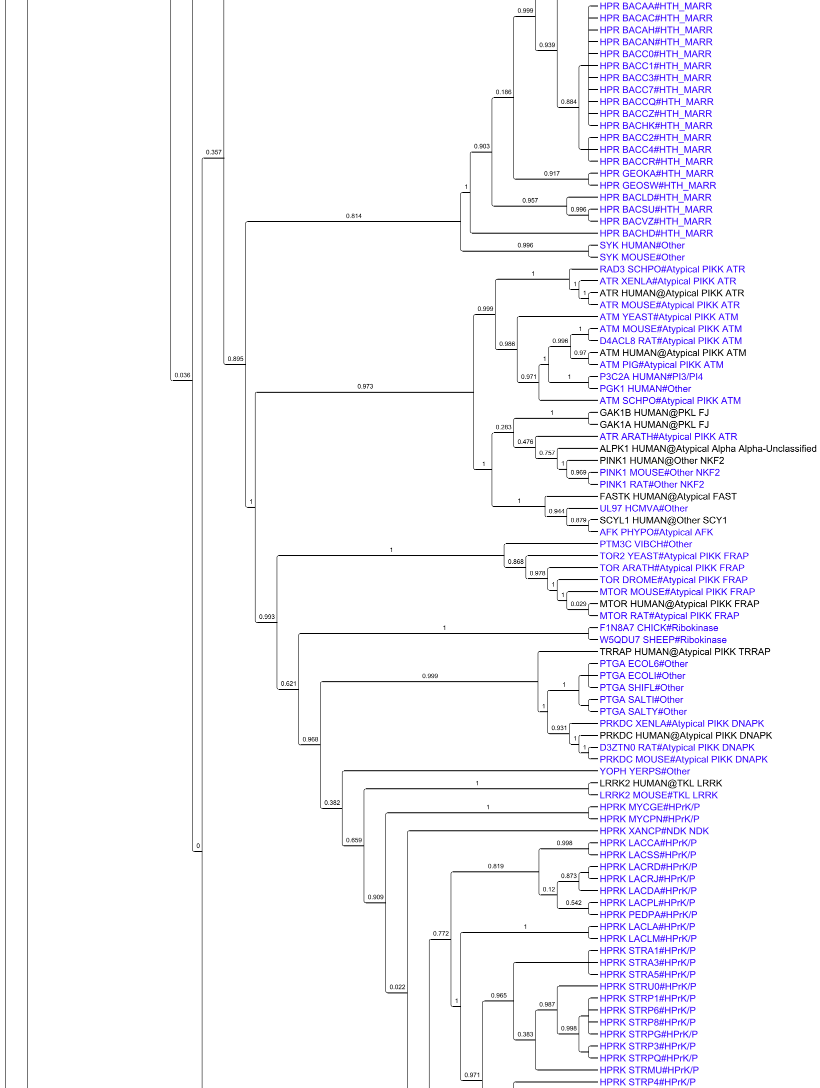
HPR BACAA#HTH_MARR
HPR BACAC#HTH_MARR
HPR BACAH#HTH_MARR
HPR BACAN#HTH_MARR
HPR BACC0#HTH_MARR
HPR BACC1#HTH_MARR
HPR BACC3#HTH_MARR
HPR BACC7#HTH_MARR
HPR BACCQ#HTH_MARR
HPR BACCZ#HTH_MARR
HPR BACHK#HTH_MARR
HPR BACC2#HTH_MARR
HPR BACC4#HTH_MARR
HPR BACCR#HTH_MARR
HPR GEOKA#HTH_MARR
HPR GEOSW#HTH_MARR
HPR BACLD#HTH_MARR
HPR BACSU#HTH_MARR
HPR BACVZ#HTH_MARR
HPR BACHD#HTH_MARR
SYK HUMAN#Other
SYK MOUSE#Other
RAD3 SCHPO#Atypical PIKK ATR
ATR XENLA#Atypical PIKK ATR
ATR HUMAN@Atypical PIKK ATR
ATR MOUSE#Atypical PIKK ATR
ATM YEAST#Atypical PIKK ATM
ATM MOUSE#Atypical PIKK ATM
D4ACL8 RAT#Atypical PIKK ATM
ATM HUMAN@Atypical PIKK ATM
ATM PIG#Atypical PIKK ATM
P3C2A HUMAN#PI3/PI4
PGK1 HUMAN#Other
ATM SCHPO#Atypical PIKK ATM
GAK1B HUMAN@PKL FJ
GAK1A HUMAN@PKL FJ
ATR ARATH#Atypical PIKK ATR
ALPK1 HUMAN@Atypical Alpha Alpha-Unclassified
PINK1 HUMAN@Other NKF2
PINK1 MOUSE#Other NKF2
PINK1 RAT#Other NKF2
FASTK HUMAN@Atypical FAST
UL97 HCMVA#Other
SCYL1 HUMAN@Other SCY1
AFK PHYPO#Atypical AFK
PTM3C VIBCH#Other
TOR2 YEAST#Atypical PIKK FRAP
TOR ARATH#Atypical PIKK FRAP
TOR DROME#Atypical PIKK FRAP
MTOR MOUSE#Atypical PIKK FRAP
MTOR HUMAN@Atypical PIKK FRAP
MTOR RAT#Atypical PIKK FRAP
F1N8A7 CHICK#Ribokinase
W5QDU7 SHEEP#Ribokinase
TRRAP HUMAN@Atypical PIKK TRRAP
PTGA ECOL6#Other
PTGA ECOLI#Other
PTGA SHIFL#Other
PTGA SALTI#Other
PTGA SALTY#Other
PRKDC XENLA#Atypical PIKK DNAPK
PRKDC HUMAN@Atypical PIKK DNAPK
D3ZTN0 RAT#Atypical PIKK DNAPK
PRKDC MOUSE#Atypical PIKK DNAPK
YOPH YERPS#Other
LRRK2 HUMAN@TKL LRRK
LRRK2 MOUSE#TKL LRRK
HPRK MYCGE#HPrK/P
HPRK MYCPN#HPrK/P
HPRK XANCP#NDK NDK
HPRK LACCA#HPrK/P
HPRK LACSS#HPrK/P
HPRK LACRD#HPrK/P
HPRK LACRJ#HPrK/P
HPRK LACDA#HPrK/P
HPRK LACPL#HPrK/P
HPRK PEDPA#HPrK/P
HPRK LACLA#HPrK/P
HPRK LACLM#HPrK/P
HPRK STRA1#HPrK/P
HPRK STRA3#HPrK/P
HPRK STRA5#HPrK/P
HPRK STRU0#HPrK/P
HPRK STRP1#HPrK/P
HPRK STRP6#HPrK/P
HPRK STRP8#HPrK/P
HPRK STRPG#HPrK/P
HPRK STRP3#HPrK/P
HPRK STRPQ#HPrK/P
HPRK STRMU#HPrK/P
HPRK STRP4#HPrK/P

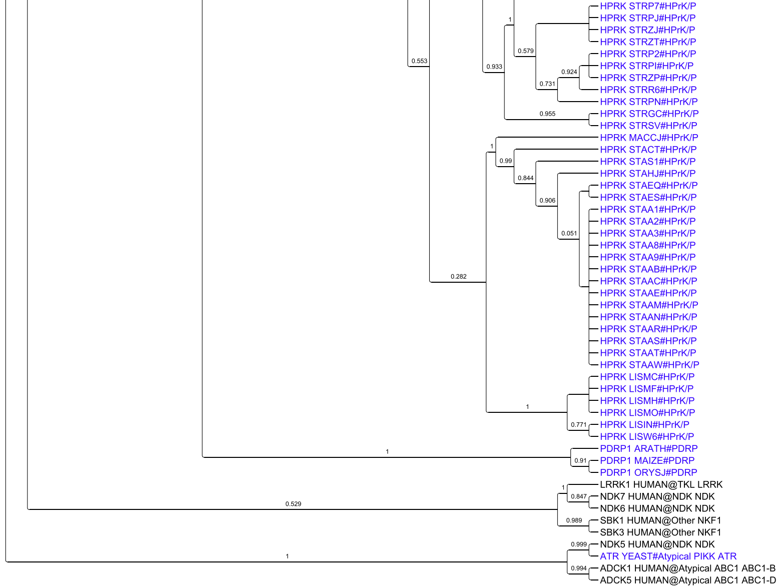
HPRK STRP7#HPrK/P
HPRK STRPJ#HPrK/P
HPRK STRZJ#HPrK/P
HPRK STRZT#HPrK/P
HPRK STRP2#HPrK/P
HPRK STRPI#HPrK/P
HPRK STRZP#HPrK/P
HPRK STRR6#HPrK/P
HPRK STRPN#HPrK/P
HPRK STRGC#HPrK/P
HPRK STRSV#HPrK/P
HPRK MACCJ#HPrK/P
HPRK STACT#HPrK/P
HPRK STAS1#HPrK/P
HPRK STAHJ#HPrK/P
HPRK STAEQ#HPrK/P
HPRK STAES#HPrK/P
HPRK STAA1#HPrK/P
HPRK STAA2#HPrK/P
HPRK STAA3#HPrK/P
HPRK STAA8#HPrK/P
HPRK STAA9#HPrK/P
HPRK STAAB#HPrK/P
HPRK STAAC#HPrK/P
HPRK STAAE#HPrK/P
HPRK STAAM#HPrK/P
HPRK STAAN#HPrK/P
HPRK STAAR#HPrK/P
HPRK STAAS#HPrK/P
HPRK STAAT#HPrK/P
HPRK STAAW#HPrK/P
HPRK LISMC#HPrK/P
HPRK LISMF#HPrK/P
HPRK LISMH#HPrK/P
HPRK LISMO#HPrK/P
HPRK LISIN#HPrK/P
HPRK LISW6#HPrK/P
PDRP1 ARATH#PDRP
PDRP1 MAIZE#PDRP
PDRP1 ORYSJ#PDRP
LRRK1 HUMAN@TKL LRRK
NDK7 HUMAN@NDK NDK
NDK6 HUMAN@NDK NDK
SBK1 HUMAN@Other NKF1
SBK3 HUMAN@Other NKF1
NDK5 HUMAN@NDK NDK
ATR YEAST#Atypical PIKK ATR
ADCK1 HUMAN@Atypical ABC1 ABC1-B
ADCK5 HUMAN@Atypical ABC1 ABC1-D
1
0.553
0.933
0.579
0.924
0.731
0.955
1
0.99
0.844
0.906
0.051
0.282
1
0.771
1
0.91
1
0.847
0.529
0.989
0.999
1
0.994
